# Pattern recognition receptors: function, regulation and therapeutic potential

**DOI:** 10.1038/s41392-025-02264-1

**Published:** 2025-07-11

**Authors:** Ruochan Chen, Ju Zou, Jiawang Chen, Xiao Zhong, Rui Kang, Daolin Tang

**Affiliations:** 1 Hunan Key Laboratory of Viral Hepatitis, Xiangya Hospital, Central South University, Changsha, Hunan China; 2 Department of Infectious Diseases, Xiangya Hospital, Central South University, Changsha, Hunan China; 3 National Clinical Research Center for Geriatric Disorders (Xiangya), Changsha, Hunan China; 4 The Department of Infectious Diseases and Tropical Medicine, the Second Affiliated Hospital of Hainan Medical University, Haikou, Hainan China; 5 Department of Surgery, UT Southwestern Medical Center, Dallas, TX USA

**Keywords:** Innate immunity, Molecular medicine

## Abstract

Pattern recognition receptors (PRRs) are sensors in the immune system, detecting pathogen-associated molecular patterns (PAMPs) and damage-associated molecular patterns (DAMPs). They serve as essential links between the innate and adaptive immune responses, initiating defense mechanisms against pathogens and maintaining immune homeostasis. This review examines the classification, structure, and signaling cascades of key PRR families, including toll-like receptors (TLRs), C-type lectin receptors (CLRs), nucleotide-binding oligomerization domain-like receptors (NLRs), AIM2-like receptors (ALRs), and others. It explores the dual roles of PRRs in immune defense and regulation, particularly through inhibitory PRRs (iPRRs), which prevent immune overactivation. The review also investigates the ligand recognition mechanisms and signaling pathways, highlighting the involvement of PRRs in disease progression and immune modulation. Notable signaling pathways, including NF-κB, MAPK, cGAS-STING, and MYD88-mediated and non-MYD88-mediated cascades, are discussed in the context of immune responses. Mechanisms that fine-tune PRR-mediated responses include transcriptional and fpost-transcriptional regulation, protein degradation, subcellular localization, and the recruitment of amplifiers and inhibitors, along with metabolic and microbial factors. These regulatory strategies ensure immune signaling remains adaptable and precise, preventing excessive inflammation. The review also explores the therapeutic potential of targeting PRRs in treating infectious, inflammatory, autoimmune, and malignant diseases, underscoring their importance in advancing immunological research and precision medicine.

## Introduction

The immune system is a highly intricate network of cells, tissues, and organs that collectively protect the body against harmful pathogens, including bacteria, viruses, fungi, and parasites, as well as abnormal cells such as those that are cancerous. The foundational understanding of the immune system stems from the pioneering work of Ilya Mechnikov and Paul Ehrlich, who jointly received the 1908 Nobel Prize in Physiology or Medicine on the two components of the mammalian immune system as we now comprehend them.^1^ Mechnikov’s discovery of phagocytosis laid the groundwork for our understanding of innate immunity. Meanwhile, Ehrlich’s investigations into immune responses against toxins and his introduction of the “sidechain theory”, wherein immune cells are stimulated to produce antibodies tailored to the invading antigen and specific antigens, significantly advanced the conceptualization of adaptive immunity.^2^ Despite these milestones, the extent of the interconnection between the innate and adaptive immunity highlighted by Mechnikov and Ehrlich remained uncertain, leading most subsequent research in the ensuing decades to concentrate on their distinct mechanisms.

**Fig. 1:**
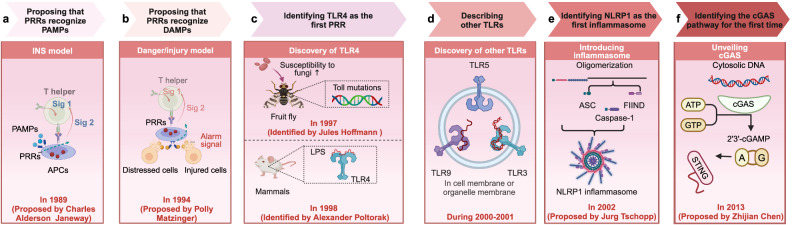
Key discoveries in PRRs. **a** The proposal of the Infectious-Non-Self (INS) model. In 1989, Charles A. Janeway introduced a revolutionary hypothesis within the self-non-self model of immunity. He proposed that the innate immune system possesses a unique capacity to detect microbial infections (named PAMPs) via receptors, which are primarily expressed on APCs and are named PRRs. **b** The proposal of the Danger/injury model. Building on the foundation established by self-non-self models, Polly Matzinger introduced and elaborated the danger/injury model in immunology in 1994. This revolutionary proposal represented a significant shift in immunological thought. It posited that PRRs can be activated not only by foreign antigens but also by endogenous cellular alarm signals (named DAMPs) released by distressed or injured cells. This insight profoundly reshaped our view of the immune system’s responsiveness to cellular distress. **c** The Discovery of TLR4. In 1997, Jules Hoffmann found that mutated “Toll” receptors in fruit flies showed increased vulnerability to fungal infections. This discovery laid the foundation for understanding the role of Toll-like receptors in the innate immune response. In 1998, Alexander Poltorak and his colleagues identified mutations in the *Tlr4* gene while investigating the defects in lipopolysaccharide (LPS) signaling in C3H/HeJ and C57BL/10ScCr mouse strains. This discovery provided definitive evidence for the pivotal role of TLR4 in LPS recognition and unveiled its significance in the innate immune response of mammals. **d** The Discovery of other TLRs. Subsequently, other TLRs including TLR3, TLR5, and TLR 9 have been discovered in succession during 2000-2001. **e** The introduction of the inflammasome. The term “inflammasome” was introduced by Jurg Tschopp and his colleagues in 2002. The first inflammasome to be identified was NACHT, LRR, and PYD domains-containing protein 1 (NLRP1) in 2002, and NLRP3 quickly followed this in 2004. **f** The discovery of cGAS. The cGAS pathway, discovered in 2013 by Professor Zhijian Chen, is capable of recognizing pathogenic double-stranded DNA molecules and serves as one of the pivotal signals in initiating antiviral innate immune defense. This finding obtained the Lasker Award for Basic Medical Research in September 2024. This figure is created by BioRender (https://app.biorender.com)

Two seminal discoveries in the late 20th century revealed the molecular and cellular crosstalk between innate and adaptive immunity: the identification of antigen-presenting cells (APCs) and the conceptualization of pattern recognition receptors (PRRs).^3^ In 1989, Charles A. Janeway introduced a revolutionary hypothesis within the self-non-self model, proposing that the innate immune system has a unique capacity to detect microbial infections via PRRs, primarily expressed on APCs.^4^ These receptors recognize molecular patterns known as pathogen-associated molecular patterns (PAMPs), absent in host cells, enabling precise discrimination between self and non-self to trigger immune responses (Fig. 1a). Building on the foundation established by self-non-self models, Polly Matzinger introduced and elaborated the danger/injury model in immunology in 1994^5–8^ (Fig. 1b). She proposed a model where the immune response is conditioned by the presence of tissue stress, damage, or infection. These factors trigger a secondary signal (signal 2) that activates APCs. These APCs, in turn, transmit a signal to T lymphocytes. Signal 1 alone is insufficient to elicit an immune response and may even induce immune tolerance. However, the combination of Signal 1 and Signal 2 effectively triggers an immune reaction. The core of this model posits that the immune system reacts not only to the presence of PAMPs but also to the danger signals emanating from cellular stress or tissue damage. When cellular damage occurs, it initiates the release of a cascade of intracellular molecules, both proteinaceous and non-proteinaceous, into the extracellular environment. These molecules are then identified by the immune system as damage-associated molecular patterns (DAMPs).

Over the past three decades since the introduction of the danger theory, immunology has made remarkable progress. A wide range of DAMPs and PAMPs typically act on various PRRs. Advances in multi-omics technologies, including single-cell transcriptomics, proteomics, metabolomics, and microbiomics, have led to the identification of an expanding array of immune and non-immune cell subsets. These subsets range from diverse epithelial cells and myeloid cell types, such as tissue-resident macrophages and dendritic cell (DC) subclasses, to lymphoid subpopulations with distinct functions and increasingly complex characteristics. PRRs expressed on these cells not only mediate potentially harmful defense responses to pathogens, which may result in inflammation and autoimmunity, but also perform an essential role in maintaining tissue homeostasis and silently eliminating premalignant or senescent cells.

The identification and profiling of PRRs and their ligands has ushered in a significant paradigm shift in comprehending the immune response to tissue damage and infection. Progressing from preliminary observations to the delineation of distinct PRR types and their pathological significance, this theory has matured into a foundational concept in immunology.^9^ This review explores the mechanistic regulation of PRRs, their role in diseases, and their possibilities as targets for treatment, providing a comprehensive understanding of their fundamental and clinical significance.

## Classification, structure, and recognition mechanism of PRRs

PRRs are host proteins encoded in the germline, existing either as membrane-bound or cytosolic entities. Their primary function is to detect invading pathogens and sterile insults.^10^ Membrane-bound PRRs encompass toll like receptors (TLRs) and C-type lectin receptors (CLRs), whereas cytosolic PRRs include nucleotide-binding oligomerization domain (NOD)-like receptors (NLRs, also known as nucleotide-binding leucine-rich repeats [LRR]), absent in melanoma 2 (AIM2)-like receptors (ALRs), retinoic acid-inducible gene I (RIG-I, previously known as DDX58)-like receptors (RLRs), and cyclic guanosine monophosphate (GMP)-adenosine monophosphate (AMP) synthase (cGAS), among others^10,11^ (Table 1). In plants, PRRs are primarily localized to the plasma membrane and are categorized into receptor-like kinases and receptor-like proteins.^12^ Both membrane-bound and cytosolic PRRs are fundamentally made up of ligand recognition domains, intermediate domains, and effector domains.^13^

**Table 1:** Types of PRRs

Types	Representative receptors
TLRs	TLR1-TLR10 (In human)
CLRs	MINCLE, DNGR1 (or CLEC9A), CLEC8A, CLEC12A, and CLEC7A (or Dectin-1)
NLRs	NLRA, NLRB, NLRC, NLRP, and NLRX1
RLRs	LGP2, MDA5, and RIG-I
Cytoplasmic DNA sensors	cGAS and AIM2
Extracellular soluble PRMs	Pentraxin (CRP, APCS, and PTX3), collectin, and ficolin
iPRRs	CD300a/f, Siglec-2, 3, and 5–11, CEACAM1, LILRB1, LILRB3, TIGIT, PVR, LAIR-1, and SIRL-1

*AIM2* absent in melanoma 2, *APCS* amyloid P component serum, *CRP* C-reactive protein, *CLRs* C-type lectin receptors, *CLEC9A* C-type lectin domain containing 9A, *CytC* cytochrome C, *CEACAM1* carcinoembryonic antigen cell adhesion molecule 1, *cGAS* cyclic GMP–AMP synthase, *DNGR1* dendritic cell natural killer lectin group receptor 1, *iPRRs* inhibitory PRRs, *LGP2* laboratory of genetics and physiology 2, *LILRB1* leukocyte immunoglobulin-like receptor subfamily B member 1, *LAIR-1* leukocyte-associated immunoglobulin-like receptor 1, *MINCLE* macrophage-inducible C-type lectin, *MDA5* melanoma differentiation factor 5, *NLRs* NOD-like receptors, *PTX3* pentraxin 3, *PRRs* pattern recognition receptors, *RLRs* RIG-I-like receptors, *RIG-I* retinoic acid-inducible gene I, *SIRL-1* signal inhibitory receptor on leukocytes 1, *TLRs* toll-like receptors, *TIGIT* T-cell immunoreceptor with Ig and ITIM domains

While tissue damage and cellular death frequently indicate pathological conditions, regulated cell death plays a crucial role in physiological processes and tissue regeneration. Consequently, the immune response necessitates subtle, context-tailored modulation to prevent excessive harm. To achieve this, the immune system must accurately interpret molecular signals from activating patterns, demanding a context-sensitive threshold for its activation. In this context, a distinct group of inhibitory receptors, known as inhibitory PRRs (iPRRs), has been identified^14^ (Table 1). These iPRRs recognize both endogenous and microbial patterns associated with danger, as well as those signaling homeostasis. By detecting PAMPs and DAMPs, iPRRs provide contextual information, facilitate a degree of microbial tolerance, and ensure a balanced response to danger signals.^15^

### TLRs

The first PRRs to be described were the TLRs.^16^ Janeway’s hypothesis gained substantial support in 1996 through research by Hoffmann, Bruno Lemaitre, and their team.^17^ They found that *Drosophila* with mutated “Toll” receptors showed increased vulnerability to fungal infections due to impaired induction of antifungal peptides^18^ (Fig. 1c). Building on this, Janeway and Medzhitov identified a human counterpart to Toll, initially termed hToll and later known as toll-like receptor 4 (TLR4). This discovery unveiled TLR4’s capacity to elicit innate responses, such as the production of pro-inflammatory cytokines and expression of co-stimulatory molecules, via activation of the transcription factor nuclear factor kappa-B (NF-κB). A mutation resulting in impaired function in the mouse homolog of hToll was discovered in mouse strains. These strains exhibited diminished innate immune responses to lipopolysaccharide (LPS), which is a major initiator of sepsis and an element of Gram-negative bacterial outer membranes. This finding, corroborated by Beutler’s group and others, underscored the critical function of TLR4 in the innate immune reactions to LPS in mice.^19^

Following this, research has uncovered 10 TLRs in humans and 12 in mice,^16,20,21^ each capable of recognizing a diverse range of PAMPs. While TLR1 to TLR9 are conserved across both species, murine TLR10 is non-functional due to the insertion of retrovirus-derived DNA. Notably, TLR11, TLR12, and TLR13 are absent from the human genome.^22–27^ TLRs are categorized into two subfamilies: cell surface and endosomal. The cell surface TLRs, encompassing TLR1, TLR2, TLR4, TLR5, TLR6, and the non-functional TLR10 in mice, recognize lipid and protein components. Conversely, the endosomal TLRs, consisting of TLR3, TLR7, TLR8, TLR9, and TLR13 (exclusive to mice), specialize in detecting nucleic acids.^28,29^ The discovery of TLRs fundamentally transformed the prevailing notion that the innate immune system’s recognition of pathogens lacked specificity. This specificity allows the innate immune system to differentiate between self and non-self while also triggering customized immune responses to a variety of microbial dangers.

**Fig. 2:**
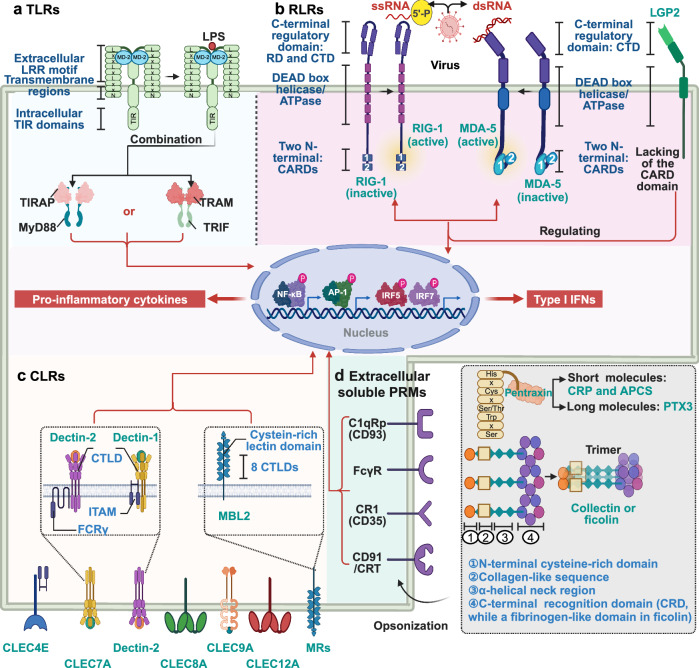
Structure of different PRRS in the cell membrane and the extracellular. **a** The structure of TLRs. TLRs featured an ectodomain adorned with LRRs that mediate signal recognition, transmembrane regions, and the cytoplasmic TIR domains that activate downstream signaling pathways. Upon recognizing PAMPs or DAMPs, TLRs interact with MYD88 or TRIF and trigger downstream signaling cascades, ultimately leading to the release of inflammatory cytokines, IFN-Is, chemokines, and antimicrobial peptides. **b** The structure of RLRs. RLRs family encompasses three members: LGP2, MDA-5, and RIG-I. Structurally, RLRs consist of two N-terminal CARDs, a central DDX/ATPase domain, and a C-terminal regulatory domain. In RIG-I, the C-terminus comprises the RD and the CTD, while MDA-5 lacks the RD, thus being devoid of self-inhibitory capabilities. In contrast to other RLR family members, LGP2 lacks the CARD domain, precluding its ability to recruit homologous molecules for signal transduction. However, researches showed that LGP2 could regulates RIG-I and MDA-5 signaling. **c** The structure of CLRs. CLRs including CLEC4E (MINCLE), CLEC7A (Dectin-1), Dectin-2, CLEC8A, CLEC9A (DNGR1), CLEC12A and MRs. Dectin-1 features an extracellular CTLD and an intracellular tail linked to an ITAM, while Dectin-2 lacks the ITAM sequence and does not possess signal transduction capabilities. MRs’ extracellular segment comprises two parts: the proximal membrane end with eight consecutive CTLDs, responsible for ligand endocytosis and transport, and the distal membrane end with a cysteine-rich lectin domain that recognizes sulfated carbohydrate conjugates. **d** The structure of Extracellular soluble PRMs. Extracellular soluble PRMs include diverse molecular families, primarily pentraxins, collectins, and ficolins. Pentraxins are categorized into two families: short and long molecules. These molecules are predominantly synthesized in the liver in response to inflammatory signals and interleukins. The long pentraxin family, represented by PTX3, uniquely features an extended N-terminal domain. Collectins and ficolins are both oligomers composed of basic subunits, each consisting of three polypeptide chains with four domains: an N-terminal cysteine-rich domain, a collagen-like sequence, an α-helical neck region, and a functional pattern recognition domain at the C-terminus. The functional domain differs between collectins and ficolins; collectins feature a globular CRD, while ficolins possess a fibrinogen-like domain. This figure is created by BioRender (https://app.biorender.com)

TLRs are members of the type I transmembrane protein family, featuring an ectodomain adorned with LRRs that mediate PAMP recognition, transmembrane regions, and the cytoplasmic toll-interleukin-1 receptor (TIR) domains that stimulate the activation of subsequent signaling pathways^30^ (Fig. 2a). Delving into the pattern recognition mechanisms of TLRs holds immense value for elucidating the intricacies of innate immunity and certain tumorigenic processes. Thus, researchers have employed X-ray crystallography to ascertain the crystallographic structures of the extracellular domains of TLRs and their respective ligand complexes. Despite variations in the structural configurations of these ligand complexes, they all exhibit a uniform M-type crystalline architecture. This finding underscores the conserved nature of TLR-ligand interactions across different complexes.^31,32^ For example, in 2007, researchers utilized X-ray crystal diffraction to scrutinize and ascertain the structural configuration of the TLR-ligand complex, offering profound insights into the architecture of the LRR domain.^33^ The LRR domain adopts a horseshoe-like shape, with each module comprising a conserved leucine motif and a region of variability. Characterized by the “LxxLxLxxN” sequence (where L represents leucine, x denotes any amino acid, and N stands for asparagine), this motif spans 20–30 amino acids and resides on the concave surface of the horseshoe-shaped structure.^34–36^ The N- and C-termini, both adopting a horseshoe configuration, are stabilized by disulfide bridges. These bridges are formed by clusters of cysteine, serving to shield the hydrophobic core.^32,37^ Upon recognizing PAMPs and DAMPs, TLRs interact with adaptor molecules possessing a TIR domain, such as MYD88 innate immune signal transduction adaptor (MYD88) and TIR domain containing adaptor molecule 1 (TICAM1, also known as TIR domain-containing adaptor-inducing interferon-β [TRIF]).^38^ This interaction triggers downstream signaling cascades, ultimately leading to the release of inflammatory cytokines, type I interferons (IFN-Is), chemokines, and antimicrobial peptides (Fig. 2a).

A significant portion of our understanding regarding structural and recognition mechanisms originates from research into interactions between mammalian TLRs and PAMPs. Across all studied mammalian TLRs, direct contact with PAMP ligands is established via interactions between their LRR-containing ectodomains and corresponding microbial products.^16,39^ Structural data reveal that ligand binding to TLRs occurs through interactions with ectodomain dimers. These dimers can form as homodimers, as observed in TLRs 3, 4, 5, 7, 8, and 9, or as heterodimers in specific combinations, such as TLR1/2, TLR2/6, and TLR5a/5b.^28^ It has been documented that TLR1 through TLR6 exist as monomeric units in solution, dimerizing only upon ligand binding. Conversely, TLR8 and TLR9 are preformed dimers, where ligand attachment triggers conformational adjustments.^40,41^ The number of PAMPs bound by individual dimers diverges: a single double-stranded RNA (dsRNA) molecule binds to TLR3 dimers,^42^ while single di- or tri-acylated lipoproteins associate with TLR2-TLR1 or TLR2-TLR6 heterodimers, respectively.^43,44^ In contrast, TLR9 dimers engage with two cytosine-phosphate-guanine (CpG)-containing DNA molecules,^45^ and each TLR4 within a homodimer interacts directly or indirectly with two LPS molecules.^46^

The affinity of the aforementioned interactions exhibits considerable variation, with certain interactions occurring at nanomolar ligand concentrations, notably for TLRs 1, 2, 3, 5, and 9.^45,47–51^ Conversely, other interactions, particularly those involving TLRs 4, 7, and 8, require higher ligand concentrations.^52,53^ High-affinity interactions, as characterized in vitro, closely align with cellular behavior, where minimal ligand concentrations are sufficient to trigger robust inflammatory responses in macrophages. In contrast, low-affinity interactions, typically requiring micromolar ligand concentrations, often fail to accurately reflect the receptor-ligand dynamics observed within cellular environments. For TLR4-LPS interactions, detailed mechanistic studies have illuminated the discrepancies between in vitro affinity measurements and the physiological responses observed in vivo. These differences highlight the complexity of receptor activation in a cellular context, where additional factors, such as co-receptors, accessory molecules, and the spatial organization of signaling complexes, significantly influence the functional outcomes of ligand binding.

### RLRs

RLRs serve a key function as sensors of nucleic acids in the cytoplasm, capable of detecting RNA virions, RNA replication intermediates, and transcription products.^54^ In the context of innate antiviral immunity, while TLR7 and TLR9 recognize viral nucleic acids, most other cell types rely on RLRs for this purpose, thereby inducing antiviral immune responses.^55,56^ This receptor family encompasses three members: DExH-box helicase 58 (LGP2, also known as laboratory of genetics and physiology 2 [LGP2]), interferon induced with helicase C domain 1 (MDA-5, also known as melanoma differentiation-associated gene 5 [MDA-5]), and RIG-I. LGP2 differs from RIG-I and MDA-5 in that it lacks the tandem CARD domains, which presumably impairs its ability to trigger signaling^57^ (Fig. 2b). This structural difference underscores the distinct roles these receptors play in innate immunity. By recognizing and responding to viral RNA, RLRs orchestrate a robust antiviral response, highlighting their critical importance in host defense mechanisms.

RLRs are essential for pathogen detection and the initiation of immune responses, underscoring their profound physiological significance. Structurally, RLRs consist of two N-terminal CARDs, a central DEAD (Asp–Glu–Ala–Asp)-box helicase (DDX)/adenosine triphosphatase (ATPase) domain, and a C-terminal regulatory domain.^58^ In RIG-I, the C-terminus comprises the repressor domain (RD) and the C-terminal domain (CTD), which together regulate the receptor’s own state.^59^ Specifically, the RD inhibits receptor activation, while the CTD is responsible for viral RNA recognition.^60^ Upon activation, RIG-I forms multimers via its tetrameric CARD, which serves as a core in the complex with mitochondrial antiviral signaling protein (MAVS), a prion-like aggregate.^61^ A recently developed artificial stem-loop RNA binds to only one RIG-I molecule, indicating that oligomer formation between the CARDs of RIG-I and MAVS is not a prerequisite for efficient IFN induction.^62^ However, further studies are needed to clarify the role of this mechanism in various viral infections in vivo.

MDA-5 shares structural and functional similarities with RIG-I, yet it lacks the RD, thus devoid of self-inhibitory capabilities. The crystal structure of MDA-5 reveals that, in contrast to RIG-I, the helicase insertion domain (HEL2i) within its central DEAD-box helicase/ATPase domain does not interact with CARD, aligning with MDA-5’s higher basal activity.^63^ The molecular mechanism preventing constitutive activation of MDA-5 remains elusive, but it may be modulated through phosphorylation of MDA-5 and/or interactions with host regulatory molecules.^64^ Structural studies have demonstrated that, unlike RIG-I, the helicase domain and CTD of MDA-5 recognize the midsection of dsRNA and assemble into a fiber-like polymer.^65^

In contrast to other RLR family members, LGP2 lacks the CARD domain,^66^ precluding its ability to recruit homologous molecules for signal transduction. Instead, LGP2 regulates the detection of viral nucleic acids by RIG-I and MDA-5, thereby preventing RLR-mediated antiviral responses.^67^ Early in vitro studies indicated that LGP2 negatively regulates RIG-I and MDA-5 signaling.^68,69^ However, subsequent research using genetically deficient mice has revealed that LGP2 actually enhances immune responses.^70^ In the presence of LGP2, the formation of MDA-5 fibers is accelerated, with LGP2 being periodically incorporated into these structures. Adenosine triphosphate (ATP) hydrolysis leads to the dissociation of both LGP2 and MDA-5 from dsRNA, and the dissociated MDA-5 retains its conformation with an exposed CARD.^71^ These unique features of LGP2 provide insight into its differential regulation of RIG-I and MDA-5, highlighting the complexity of innate immune responses to viral infections.

### CLRs

In the early 2000s, a new family of PRRs emerged with the discovery of CLRs. The identification of CLRs was not driven by efforts to uncover inflammatory signaling mechanisms similar to those of TLRs, but rather by research aimed at elucidating the mechanisms of phagocytosis, a cellular process in which certain cells, primarily immune cells, engulf and internalize solid particles such as pathogens, dead cells, or debris.^72^ Specifically, Brown and colleagues, through an expression cloning screen designed to identify a receptor enabling macrophages to phagocytose yeast cell wall particles (zymosan), identified a protein initially known as dendritic cell (DC)-associated C-type lectin-1 (Dectin-1).^73^ Further investigation revealed that this receptor binds to β-glucan, a structural carbohydrate polymer found in fungal cell walls, thereby establishing it as a prime example of a PRR.

To date, over one thousand members within the C-type lectin family have been identified (including those from genome sequence only), such as members of the dectin family including C-type lectin domain family 4 member E (CLEC4E, also known as macrophage-inducible C-type lectin [MINCLE]), C-type lectin domain containing 9A (CLEC9A, also known as dendritic cell natural killer lectin group receptor 1 [DNGR1]), and others like oxidized low density lipoprotein receptor 1 (OLR1, also known as CLEC8A), C-type lectin domain family 12 member A (CLEC12A), and C-type lectin domain containing 7A (CLEC7A, also known as Dectin-1), implicated in recognizing PAMPs and endogenous DAMPs linked with cell death, inflammatory diseases, and antitumor immunity^74,75^ (Fig. 2c). All members of this receptor family share one or more C-type lectin-like domains (CTLDs) and were initially classified based on their capacity to Attach to carbohydrates in a manner reliant on Ca^2+^. This binding occurs via structurally conserved residues within the CTLD, notably the EPN (Glu-Pro-Asn) motif, which facilitates mannose recognition, and the QPD (Gln-Pro-Asp) motif, which enables galactose recognition.^76–79^ Subsequent analyses have uncovered numerous CTLDs that function independently of Ca^2+^ and lack the traditional motifs necessary for carbohydrate recognition. In summary, CTLDs typically possess a compact, globular structure stabilized by a double loop reinforced by highly conserved disulfide bridges. Beyond their well-known role in carbohydrate recognition, CTLDs exhibit remarkable versatility, binding a wide range of ligands, including proteins and lipids such as glycolipids. They also interact with diverse structures, including fungal melanin and inorganic molecules like uric acid (UA). Some fish antifreeze proteins utilize their CTLDs to recognize and bind to ice crystals, highlighting the functional diversity of this domain across different biological contexts.^80^

CLRs are primarily expressed on myeloid cells, including macrophages, DCs, and neutrophils, and they exist in both transmembrane and soluble forms.^78^ Dectin-1 and Dectin-2, which are DC-associated C-type lectins, serve as characteristic examples of the CLR family.^81,82^ Dectin-1, a type II transmembrane protein found in DCs, macrophages, neutrophils, and monocytes,^83^ features an extracellular CTLD and an intracellular tail linked to an immunoreceptor tyrosine-based activation motif (ITAM), indicating it signaling function. In contrast, Dectin-2 lacks the ITAM sequence and does not possess signal transduction capabilities^82^ (Fig. 2c). Dectin-2 mainly identifies α-mannan in fungal cell walls and the antigen from Schistosoma mansoni eggs.^84,85^

Mannose receptors (MRs) are membrane-bound CLRs that are single-chain molecules that span the cell membrane.^86^ The extracellular portion of the MR consists of two sections: the region near the membrane, which includes eight consecutive CTLDs that handle ligand endocytosis and transport, and the region farther from the membrane, featuring a cysteine-rich lectin domain that detects sulfated carbohydrate complexes^86^ (Fig. 2c). MR’s endogenous ligands include lysosomal hydrolase, myeloperoxidase, and pathogen-expressed mannan-rich structures.^87^ Collectins, C-type lectins with collagenous regions, are soluble, secreted pattern recognition proteins that directly attach to microbes through their CTLDs, enhancing phagocytosis. The most renowned collectin is mannose binding lectin 2 (MBL2, also known as MBL or mannan-binding protein [MBP]), a liver-produced serum protein. MBL2 is composed of amino-terminal collagen-like domains, a brief neck segment, and a carboxy-terminal CTLD, assembling into a bouquet of three to six trimers.^88^ It attaches to carbohydrates like mannose, N-acetylmannosamine, N-acetylglucosamine, glucose, and fucose found on the cell walls of various microbes.^89^

Many CLRs display distinct expression patterns in myeloid cells, making them valuable tools for identifying specific myeloid subpopulations, either individually or in combination with other cellular markers. For instance, mannose receptor C-type 1 (MRC1), when analyzed via single-cell RNA sequencing and spatial transcriptomics,^90^ functions as a marker for “immunosuppressive macrophages” characterized by the coexpression of MRC1, C-C motif chemokine ligand 18 (CCL18), and an M2-like phenotype. Cluster of differentiation 207 (CD207, also known as Langerin), on the other hand, is predominantly expressed by Langerhans cells,^91^ while CLEC9A specifically defines type 1 conventional DCs.^92^ However, none of these CLRs are entirely specific to a single-cell type, and both their expression patterns and the genes themselves can vary across species. Despite this variability, the restricted expression patterns of CLRs can be leveraged to identify and manipulate specific myeloid cell types. For instance, a genetically modified mouse model was developed by inserting the diphtheria toxin receptor into the CD207 locus, allowing for the selective depletion of epidermal Langerhans cells in experiments. In addition, antibodies targeting CD209, lymphocyte antigen 75 (LY75, also known as DEC-205), or CLEC9A can be utilized to transport antigens to DCs for cross-presentation.^93,94^ This approach holds significant potential for developing strategies to modulate immune responses in therapeutic and research applications.

### cGAS

In mammals, cGAS functions as a PRR that detects dsDNA aberrantly localized in the cytosol. It recognizes DNA in a manner that is independent of sequence, enabling it to detect DNA from diverse sources. This includes DNA from infecting microbial genomes,^95^ DNA generated through the reverse transcription of viral RNA,^96^ and even host DNA that escapes into the cytoplasm due to DNA damage or compromised nuclear or mitochondrial membranes.^97^

Given the pivotal role of the cGAS enzyme in DNA sensing, there has been substantial interest in elucidating the molecular details of its activation and functionality. Upon engaging with the sugar-phosphate backbone of dsDNA, cGAS transitions into a catalytically active state.^98^ As a nucleotidyltransferase, cGAS catalyzes the formation of phosphodiester bonds akin to DNA polymerase beta (POLB), but with a distinctive deep catalytic pocket shaped by an alpha-helical CTD that facilitates the synthesis of cyclic di-nucleotides (CDNs). cGAS possesses two primary DNA-binding regions, enabling it to form a 2:2 dimer with DNA.^99^ DNA-binding triggers conformational shifts, exposing the catalytic site for nucleotide-binding and aligning essential residues for catalysis. While cGAS can bind short (17 bp) DNA segments, these fragments generally fail to stimulate cGAS activity, with the exception of certain Y-shaped single-strand DNAs containing unpaired guanine residues, which may activate cGAS via an undetermined mechanism.^100^ For robust enzymatic activity, longer stretches of dsDNA (exceeding 50 bp) are necessary.^101^ These longer DNA molecules promote higher-order oligomerization of cGAS, which is believed to further stabilize the active dimer conformation.^102^

**Fig. 3:**
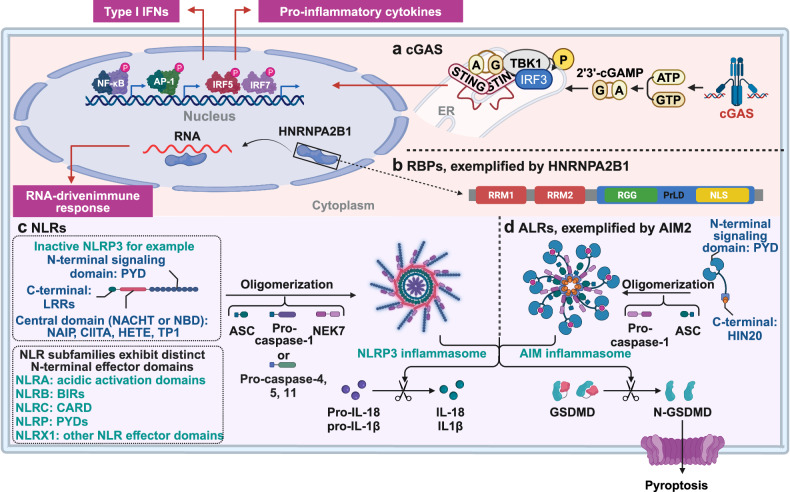
Structure of different PRRS in the cytoplasm and nucleus. **a** The structure of cGAS. Upon cytosolic DNA detection, cGAS synthesizes 2'3’-cGAMP from ATP and GTP. The produced 2'3’-cGAMP acts as a second messenger, binding to and activating the ER-resident protein STING. This interaction leads to the dimerization and autophosphorylation of STING, which then recruits and activates TBK1. Subsequently, TBK1 phosphorylates the transcription factor IRF3, promoting its dimerization, nuclear translocation, and activation of ISGs. This pathway is crucial for the induction of type I interferon responses against intracellular pathogens. **b** The functional domains of HNRNPA2/B1. The N-terminal of HNRNPA2/B1 proteins contains two RNA recognition motifs (RRM1 and RRM2), and the C-terminal is a glycine-rich low-complexity region (LC) containing an RGG box, an M9 nuclear localization signal (NLS), and a core prion-like domain (PrLD), reference from.^602^ **c** The structure of NLRs. NLRs are composed of three main domains: one is the central nucleotide-binding domain, also known as the NACHT domain (synthesized by the abbreviations of the following four kinds of NLR members: NAIP, CIITA, HETE, and TEP1; LRRs at the C-terminus, which are used to identify ligands; and the N-terminal effector domain, which is the protein interaction domain, such as CARD or PYD. Based on their distinct N-terminal effector domains, the NLRs can be categorized into five subfamilies. NLRA, NLRB, NLRC, NLRP, and NLRX1. **d** The structure of CLRs, exemplified by AIM. Structurally, AIM2 consists of a C-terminal HIN-200 domain and an N-terminal PYD. Acting as a cytosolic DNA receptor, AIM2 directly interacts with dsDNA through its HIN-200 domain. The PYD of AIM2 engages with the corresponding PYD of the inflammasome adapter protein ASC. Subsequently, the CARD of ASC connects with the CARD of procaspase-1, leading to the formation of the AIM2 inflammasome. This figure is created by BioRender (https://app.biorender.com)

Upon DNA recognition, cGAS accelerates the synthesis of the nucleotide second messenger 2'3’-cyclic GMP–AMP (cGAMP), which directly interacts with and activates the receptor protein stimulator of interferon genes (STING). This interaction triggers interferon regulatory factor 3 (IRF3)- and NF-κB-dependent transcriptional responses^103–107^ (Fig. 3a). The cGAS–STING signaling pathway is evolutionarily ancient, tracing its origins to bacteria as a potent antiviral defense mechanism.^108^ Bacteria encode a vast array of cGAS/DncV-like nucleotidyltransferase (CD-NTase) enzymes that regulate diverse anti-phage defense signaling pathways.^101^ Recently, a fully functional cGAS-like signaling pathway was uncovered in Drosophila. In this context, cGAS-like receptor 1 (cGLR1) acts as a PRR that detects dsRNA and synthesizes 3'2’-cGAMP to activate STING, thereby restricting viral replication.^109,110^ Given the extensive diversity of CD-NTase enzymes in bacterial anti-phage defense, the discovery of cGLR1 in Drosophila suggests a broader role for cGAS homologs in innate immunity across the animal. This finding underscores the evolutionary conservation and functional diversity of cGAS-mediated immune responses.

A recent study has also established cGAS-like receptors (cGLRs) as a prominent family of PRRs in animal innate immunity. This study identified over 3000 cGLRs, all possessing predicted complete catalytic active sites, spanning nearly all major animal species. Utilizing a forward biochemical screening approach, the researchers successfully reconstituted cGLR signaling from representatives across the protein family tree and discovered 16 active animal cGLRs. Remarkably, cGLRs in diverse animal genomes were found to respond to the universal PAMPs, such as dsDNA and dsRNA, synthesizing nucleotide second messenger immune signals that often contain pyrimidine bases. The variety of cGLR nucleotide second messengers and STING receptors enables animals to build complex networks for detecting pathogens. Moreover, the research revealed a conserved immune gene transcription program across evolution that is regulated by the activation of cGLR signaling in vivo. Overall, these results clarify the molecular mechanisms regulating cGLR signaling in animal innate immunity and reveal how the diversification of an ancient cGAS-like nucleotide signaling domain has driven the evolution of pathogen sensing.^111^

### NLRs

NLRs, a conserved group of innate immune receptors, were initially recognized for their role in responding to intracellular pathogens and endogenous byproducts resulting from tissue damage.^112^ Thus far, 22 NLRs with diverse operational functions have been recognized in humans.^113^ Structurally, NLRs are made up of three main domains: one is the central nucleotide-binding domain, also known as the NACHT domain (synthesized by the abbreviations of the following four kinds of NLR members: NLR family apoptosis inhibitory protein [NAIP, also known as NLRB1], class II major histocompatibility complex trans-activator [CIITA, also known as NLRA], hydroxy eicosatetraenoic [HETE], telomerase associated protein 1 [TEP1, also known as TP1]), which is a common feature of the NLR family and plays a crucial role in nucleic acid binding and the oligomerization of NLRs; LRRs at the C-terminus, which are employed to detect ligands; and the N-terminal effector domain, which serves as the protein-binding region, such as the caspase recruitment domain (CARD) or the pyrin domain (PYD)^114^ (Fig. 3c). Based on their distinct N-terminal effector domains, the NLRs can be categorized into five subfamilies: the NLR family CARD domain containing (NLRC) subfamily, characterized by CARDs; the NLR family pyrin domain containing (NLRP) subfamily, featuring PYDs; the NLRB subfamily, possessing baculovirus inhibitor of apoptosis protein repeats (BIRs); the NLRA subfamily, characterized by the presence of acidic activation domains; and the NLRX subfamily, which includes other NLR effector domains.^10^ In response to pathogens, PAMPs, DAMPs, and homeostatic disturbances, NLRs drive potent innate immune responses and assemble cell-death-inducing complexes. In addition, NLRs regulate the expression of major histocompatibility complex (MHC) class-I and -II genes for antigen presentation, establishing a critical link to the activation of adaptive immunity. Certain NLRs contribute to reproductive biology and development, while others negatively regulate NF-κB and interferon (IFN) signaling to maintain homeostasis and prevent excessive inflammation. Collectively, NLR proteins exhibit broad and diverse pro- and anti-inflammatory functions, highlighting their critical role in immune regulation.

Within the NLR family, significant research has been dedicated to NLRC1 and NLRC2, also known as nucleotide-binding oligomerization domain containing 1 (NOD1) and NOD2, respectively. These proteins belong to the NLRC subfamily, characterized by their CARD domains, and were the first mammalian NLRs to be characterized. They play pivotal roles in innate immune responses. NOD1 directly interacts with the bacterial peptidoglycan ligand l-Ala-γ-d-Glu-meso-diaminopimelic acid (Tri-DAP),^115^ whereas NOD2 engages with muramyl dipeptide.^116^ Upon recognizing their respective PAMPs, NOD1 and NOD2 oligomerize and recruit receptor-interacting serine/threonine kinase 2 (RIPK2, also known as RIP2) via their CARD domains.^117^ Although NOD1 and NOD2 lack transmembrane domains, they are actively recruited to the plasma and endosomal membranes, a process essential for effective signal transduction.^118^ In this context, palmitoylation acts as a key regulatory mechanism in this system. This post-translational modification, mediated by the palmitoyltransferase zinc finger DHHC-type palmitoyl transferase 5 (ZDHHC5), is critical for enabling NOD1 and NOD2 to mount an effective immune response against bacterial peptidoglycans.^119^ Palmitoylation allows these proteins to rapidly and reversibly alter their subcellular localization, a feature essential for their membrane recruitment and the initiation of inflammatory signal transduction.^119^ These findings underscore the critical role of post-translational modifications in regulating PRR-mediated innate immune signaling and offer valuable insights into their functional dynamics in host defense.

### ALRs

AIM2-like receptors (ALRs) represent a cytosolic class of PRRs that are capable of detecting intracellular DNA, thereby impacting immunometabolism.^120^ AIM2 remains the most extensively studied member of ALR family.^121,122^ Structurally, AIM2 comprises a C-terminal hematopoietic interferon-inducible nuclear (HIN)-200 domain and an N-terminal PYD. It directly engages double-stranded DNA (dsDNA) through its HIN-200 domain. The PYD of AIM2 interacts with the corresponding PYD of the inflammasome adapter protein PYD and CARD domain containing (PYCARD, also known as apoptosis-associated speck-like protein containing a CARD [ASC]). Subsequently, the CARD of ASC links with the CARD of procaspase-1, culminating in the assembly of the AIM2 inflammasome. This complex further triggers the activation of caspase-1, which in turn catalyzes the maturation of downstream cytokines interleukin-1β (IL-1β) and IL-18, as well as the cleavage of gasdermin D (GSDMD)^123^ (Fig. 3d).

AIM2 binds dsDNA in a manner independent of both sequence and structural parameters but requires a minimum length of 80 base pairs for efficient inflammasome activation.^124^ Both AIM2-DNA interaction and inflammasome activity are contingent upon dsDNA, and it can assemble into filamentous structures along dsDNA. The minimal oligomer assembly requires 6 protomers, whereas optimal oligomerization demands 24 protomers for full functionality. Interestingly, at elevated protein concentrations, AIM2 forms filaments even in the absence of dsDNA.^125,126^ These dsDNA-free AIM2 filaments exhibit a Brussels sprout-like structure, where the core stems are constituted by HIN domain filaments, and multiple PYD clusters form the “sprouts”.^127^ The architectural assembly of DNA-free AIM2 filaments implies an intrinsic affinity between HIN domains. Although it remains uncertain whether the same filamentous structure occurs in DNA-bound AIM2, these findings collectively endorse a model where filament assembly is orchestrated by the cooperative actions of HIN-dsDNA binding, HIN–HIN interactions, and PYD–PYD interactions. Subsequently, the PYD oligomer recruits the adapter protein ASC via AIM2^PYD^-ASC^PYD^ interactions, nucleating the formation of ASC filaments. Ultimately, ASC recruits caspase-1 through CARD-CARD interactions, thereby completing the inflammasome assembly.^125,128^

Under normal physiological conditions, AIM2 exists in an autoinhibited state through intramolecular interactions between its PYD and HIN domains. This structural arrangement prevents unintended inflammasome activation. Upon binding of dsDNA to the HIN domain, this autoinhibition is disrupted, exposing the PYD. The exposed PYD then facilitates the recruitment of the adaptor protein ASC, which interacts via its PYD to form the AIM2 inflammasome. AIM2’s inflammasome-dependent function plays a crucial role in sensing microbial DNA during infections and is implicated in tumorigenesis and a range of inflammatory and autoimmune disorders, including atherosclerosis, neuroinflammation, psoriasis, dermatitis, arthritis, systemic lupus erythematosus (SLE), liver disease,^129,130^ and colitis. Further understanding the molecular mechanisms governing AIM2 activation and its regulation could provide critical insights for therapeutic interventions targeting AIM2-driven diseases.

### Extracellular soluble PRMs

The activation of the innate immune response relies on pattern recognition molecules (PRMs) detecting PAMPs, which include cellular PRRs and extracellular soluble PRMs.^131^ These molecules belong to a class of soluble receptors that exert antibacterial effects in the serum. PRMs in the fluid phase are thought to represent evolutionary precursors to antibodies, offering immune protection and modulation through opsonization, neutralization, enhancement of phagocytosis, and activation of the complement system. In addition, they engage with cell-associated PRRs, regulating their activity to coordinate a unified innate immune response.^132^ Beyond their roles in pathogen identification and elimination, PRMs are increasingly recognized as pivotal players in tissue remodeling. In this context, they detect DAMPs and transmit biochemical signals for the clearance of cellular debris and tissue regeneration.^133^ This wide range of functions underscores the crucial role of PRMs in maintaining immune homeostasis and preserving tissue integrity.

Extracellular soluble PRMs include diverse molecular families, primarily including pentraxin, collectin, and ficolin.^134–137^ Pentraxins are characterized by their quintet aggregation and evolutionary conservation, featuring a distinct pentraxin signature of five molecules (His-x-Cys-x-Ser/Thr-Trp-x-Ser) and typical quaternary structures.^138^ They are categorized into two groups: short and long molecules. The short pentraxin family, exemplified by C-reactive protein (CRP) in humans and amyloid P component serum (APCS, also known as SAP) in mice, functions as acute-phase proteins. These molecules are predominantly synthesized in the liver in response to inflammatory signals and interleukins. CRP specifically binds to phosphocholine on pathogenic microorganisms in a Ca^2+^-dependent manner,^139^ while serum amyloid A (SAA) interacts with bacterial outer membrane protein A and TLRs.^140^ The long pentraxin family, represented by pentraxin 3 (PTX3), uniquely features an extended N-terminal domain. PTX3 production is regulated by various inflammatory factors in DCs, macrophages, smooth muscle cells, and endothelial cells (ECs).^141^

Collectins and ficolins are both oligomers made up of basic subunits, each of which contains three polypeptide chains and has four domains: an N-terminal cysteine-rich domain, a collagen-like sequence, an α-helical neck region, and a functional (pattern recognition) domain at the C-terminus.^142^ The functional domain differs between collectins and ficolins, with collectins featuring a globular carbohydrate-recognition (lectin) domain (CRD) and ficolins possessing a fibrinogen-like domain. The collagen-like region comprises repeating Gly–X–Y triplets (where X and Y are any amino acids), and their activity generally requires calcium ions.^143^ Collectins primarily include MBL2 and surfactant protein, whose CRD domains can recognize sugar structures on various pathogens, such as mannose, fucose, and glucose, across yeast, parasites, and bacteria.^144^ The biological significance is that they can selectively identify microbial carbohydrate structures that are harmful to themselves. Ficolins, an essential component of innate immunity, activate the complement system via the lectin pathway.^137^ Three human ficolins (ficolin-1, ficolin-2, and ficolin-3) and two murine ficolins (ficolin-A and ficolin-B) have been identified as key components of the innate immune system. Ficolins function as PRMs by recognizing PAMPs on microorganisms. They form complexes with MBL2, other ficolins, and collectin-associated serine proteases (MASPs), thereby activating the lectin pathway of the complement system. Their known ligands include N-acetylglucosamine and lipoteichoic acid (LTA), a predominant structural constituent of Gram-positive bacterial cell walls, highlighting their role in targeting a broad range of pathogens for immune defense.^145^

### RNA- and DNA-binding proteins recognizing viral nucleic acids

RNA-binding proteins (RBPs) are pivotal in gene expression regulation at the transcript level by recognizing specific RNA motifs and controlling the fate of multiple transcripts simultaneously, thereby serving as master regulators of gene expression and promising therapeutic targets.^146,147^ Recently, RBPs have been identified as novel sensors of viral nucleic acids. Specifically, the functions of heterogeneous nuclear ribonucleoprotein A2/B1 (HNRNPA2B1) have been elucidated through in vitro and in vivo experiments using myeloid cell-specific *Hnrnpa2b1*-knockout (KO) mice^148^ (Fig. 3b). These studies have revealed the dual role of HNRNPA2B1 in nuclear and cytoplasmic innate immune responses. Upon detecting viral DNA in the nucleus, hnRNPA2B1 translocates to the cytoplasm, where it triggers type I interferon (IFN-I) production, thereby suppressing DNA virus infection. It also facilitates the nucleocytoplasmic translocation and expression of key messenger RNAs (mRNAs) like CGAS, interferon-γ (IFN-γ) inducible protein 16 (IFI16), and STING, which amplify antiviral signaling. In its basal state, HNRNPA2B1 regulates RNA-associated processes; however, when encountering viral DNA, it transitions to mediate the nuclear innate immune response, activating cytoplasmic TANK binding kinase 1 (TBK1) and managing mRNA transport for innate immune receptors. The HNRNPA2B1 detects adenine metabolites, enhancing antimicrobial responses by demethylating the *Il1b* enhancer chromatin, thus increasing its accessibility.^149^

Moreover, recent studies have elucidated the crucial roles of HNRNPA2B1 in the regulation of neurodegenerative diseases and cancer progression. Pathogenic mutations within the prion-like domains of HNRNPA2B1 and HNRNPA1 facilitate their assembly into self-seeding fibrils and enhance their incorporation into stress granules, ultimately leading to neurodegenerative conditions such as multisystem proteinopathy and amyotrophic lateral sclerosis.^150^ N6-methyladenosine (m6A) constitutes the most prevalent internal modification of messenger RNA. HNRNPA2B1 directly binds to a subset of nuclear transcripts and induces alternative splicing effects akin to those mediated by the m6A writer methyltransferase 3, N6-adenosine-methyltransferase complex catalytic subunit (METTL3, previously known as methyltransferase like 3), which functions as a nuclear reader of the m6A mark to regulate pivotal nuclear RNA processing steps, thereby influencing RNA fate and function.^151^ Furthermore, mechanistic investigations have revealed that HNRNPA2B1 serves as a linker, bridging the activity of oligomeric microtubule-associated protein tau (oTau) with m6A-modified RNA transcripts. The knockdown of HNRNPA2B1 impedes the association of oTau or tau oligomers with m6A, prevents the reduction of protein synthesis, and attenuates oTau-induced neurodegeneration.^152^ In addition, HNRNPA2B1 promotes the progression of multiple myeloma by stabilizing interleukin enhancer-binding factor 3 mRNA through m6A recognition and regulating AKT3 expression, underscoring its potential as a therapeutic target.^153^ In summary, HNRNPA2B1 emerges as a promising therapeutic target and biomarker for future clinical applications.

The role of ribosomal protein SA (RPSA), also known as the 37-kDa laminin receptor precursor (37LRP), in antiviral immunity involves recognizing viral nucleic acids in the nucleus and enhancing the expression of pro-inflammatory cytokines.^154^ Functional screening in herpes simplex virus type 1 (HSV)-1-infected mouse peritoneal macrophages using an RNA interference library revealed that RPSA knockdown significantly reduces *Il1b* expression, underscoring its importance in viral-induced inflammatory responses.^155^ Mechanistic studies have shown that RPSA, upon infection, undergoes Tyr204 phosphorylation and recruits the SNF2-related chromatin remodeling ATPase 5 (SMARCA5) subunit of the imitation switch (ISWI) complex, thus promoting NF-κB binding to target gene promoters without altering other innate pathways. Future investigations might focus on the therapeutic potential of targeting nuclear RPSA, particularly around the Y204 residue, to modulate its function in viral infections.

### iPRRs

**Fig. 4:**
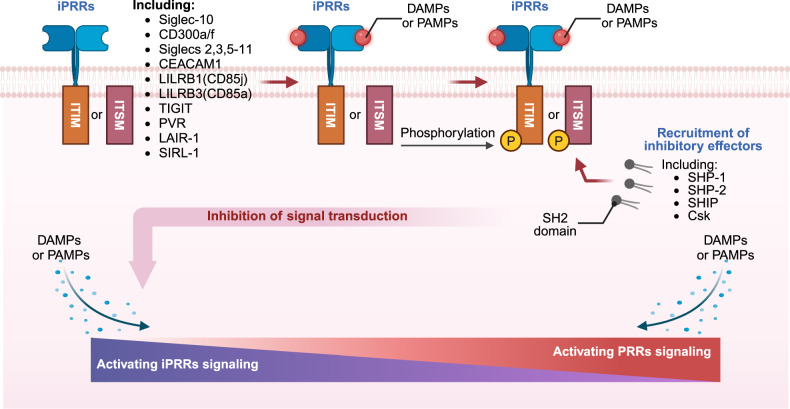
The signaling pathways mediated by iPRRs and their regulation. iPRRs predominantly transmit inhibitory signals through motifs of ITIMs or ITSM located in their cytoplasmic tails. Upon receptor ligation, these motifs undergo phosphorylation, triggering the recruitment of inhibitory effectors containing SH2 domains, such as SHP-1, SHP-2, SHIP, and Csk. The known group of iPRRs currently includes Siglec-10, CD300a/f, Siglecs 2,3,5–11, CEACAM1, LILRB1 (CD85j), LILRB3 (CD85a), TIGIT, PVR, LAIR-1, and SIRL-1. In scenarios where tolerating damage is advantageous for the host, DAMPs or PAMPs can trigger iPRRs to dampen the immune response. Conversely, when damage cannot be tolerated, DAMPs or PAMPs signal via activating PRRs to initiate a robust immune response. The relative abundance of PRRs and iPRRs, along with their corresponding ligands, dictates the intensity of the ensuing immune reaction. This figure is created by BioRender (https://app.biorender.com)

To distinguish between harmless and potentially harmful stimuli, the immune system must accurately interpret activating signals conveyed by molecular patterns. Consequently, the activation threshold of the immune system needs to be context-dependent and adaptable. iPRRs play a critical role in this context, sensing by modulating immune cell responses. For example, iPRRs raise the activation threshold to prevent excessive immune responses to benign stimuli, thereby minimizing the risk of inappropriate inflammation. Thus, iPRRs serve as regulatory counterparts to activating PRRs, ensuring a balanced and context-appropriate immune response. Most iPRRs are typically studied on hematopoietic cells, and extensive insights into their functions have been gained through the study of programmed cell death 1 (PDCD1, also known as PD-1),^156^ cytotoxic T-lymphocyte associated protein 4 (CTLA4),^157^ indoleamine 2,3-dioxygenase 1 (IDO1)^158^ and killer cell Ig-like inhibitory receptors on natural killer (NK) cells.^159^ These receptors predominantly transmit inhibitory signals through one or more immunoreceptor tyrosine-based inhibitory motifs (ITIMs) located in their cytoplasmic tails.^160^ Furthermore, numerous receptors of this kind are found on non-hematopoietic cells, particularly on epithelial and endothelial cells.^161^ From a mechanistic perspective, inhibitory receptors typically harbor an ITIM or, in certain instances, an immunoreceptor tyrosine-based switch motif (ITSM) within their cytoplasmic tails. Upon receptor ligation, these motifs undergo phosphorylation, triggering the recruitment of inhibitory effectors containing Src homology 2 (SH2) domains. These effectors include protein tyrosine phosphatase non-receptor type 6 (PTPN6, also known as SH2 domain-containing protein tyrosine phosphatase 1 [SHP-1]), PTPN11 (also known as SHP-2), inositol polyphosphate-5-phosphatase D (INPP5D, also known as SH2 domain-containing inositol 5’phosphatase 1 [SHIP1] or SHIP), and C-terminal Src kinase (CSK). Consequently, they inhibit signaling through activating receptors^162,163^ (Fig. 4).

The known group of iPRRs currently includes CD300a/f, sialic acid binding Ig like lectins (Siglecs) 2, 3, and 5–11, carcinoembryonic antigen related cell adhesion molecule 1 (CEACAM1, also known as CD66a), leukocyte immunoglobulin-like receptor B1 (LILRB1) and LILRB3, T cell immunoreceptor with Ig and ITIM domains (TIGIT), PVR cell adhesion molecule (PVR, previously known as poliovirus receptor), leukocyte-associated immunoglobulin-like receptor 1 (LAIR1), and signal inhibitory receptor on leukocytes 1 (SIRL-1)^14^ (Fig. 4). Numerous inhibitory receptors are essential for modulating inflammatory responses triggered by PAMPs and DAMPs, as they dampen activating signals and precisely regulate immune cell activation levels. CEACAM1 serves as a prominent example, exhibiting broad epithelial expression. This immunoregulatory molecule suppresses immune activation in epithelial cells by specifically inhibiting TLR2-mediated IL-8 production in airway epithelium upon engagement with its endogenous ligand CEACAM8.^164^ In addition, it can inhibit antibacterial responses triggered by TLR2 in human pulmonary epithelial cells when interacting with microbial ligands from *Moraxella catarrhalis* and *Neisseria meningitidis*.^165^ Interestingly, some activating PRRs can also exhibit inhibitory functions under specific conditions. For instance, For instance, while TLR4 signaling from the cell membrane typically elicits pro-inflammatory responses, signaling from the endosome can trigger anti-inflammatory responses.^166^ This dual functionality highlights the potential of iPRRs as therapeutic targets for the treatment or prevention of various diseases. In addition, the regulation of iPRR activity can modulate and constrain inflammatory responses, emphasizing their significance in managing inflammation-associated conditions.

## Cellular distributions of PRRs

**Fig. 5:**
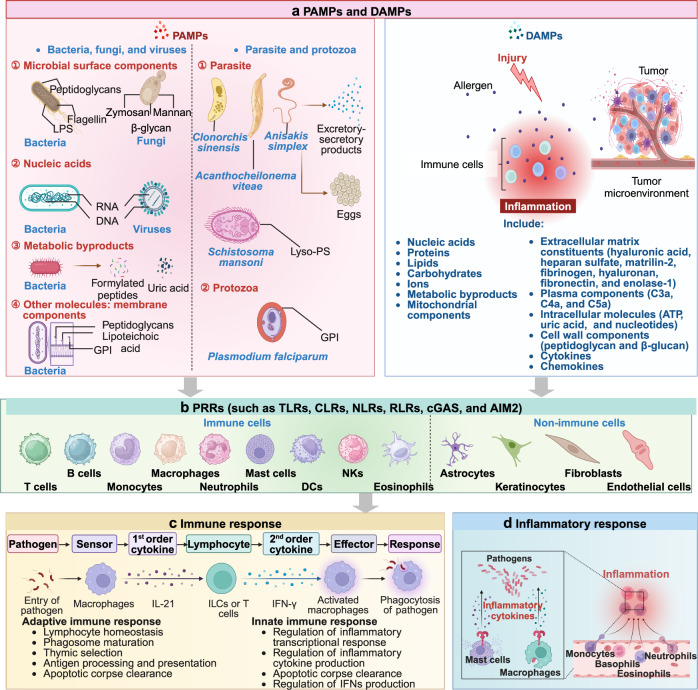
PAMPs and DAMPs play critical roles in immune response and inflammatory response. Various DAMPs or PAMPs (**a**) are recognized by PRRs present on various immune or non-immune cells (**b**). This recognition initiates a cascade of downstream signaling pathways, ultimately culminating in the release of a diverse spectrum of cytokines, chemokines, adhesion molecules, and other inflammatory mediators. In unison, these processes orchestrate the initiation, activation, and amplification of immune response (**c**) and inflammatory response (**d**), thereby facilitating a coordinated response to tissue injury or stress. This figure is created by BioRender (https://app.biorender.com)

PRRs are broadly expressed across diverse cell types, including immune cells and non-immune cells such as epithelial cells and ECs (Fig. 5). Below, we summarize their expression patterns and functions in various cell types.

### Non-immune cells

Similar to immune cells, non-hematopoietic cells are capable of recognizing microbial and endogenous threats, with their responses to these stimuli being highly context-dependent. For example, the intact intestinal epithelium expresses PRRs but must remain tolerant to commensal bacteria under normal conditions. However, in the event of epithelial damage, a rapid immune response is required to maintain tissue integrity. This highlights the ability of non-immune cells to sense environmental cues and modulate their responses via PRRs. In this discussion, we focus on the cellular distribution of PRRs in epithelial and endothelial cells. Positioned within barrier tissues, these cell types provide an excellent model for understanding the regulatory mechanisms of PRRs and their adaptive advantages in maintaining tissue homeostasis and defense.

Epithelial cells’ immune function majorly includes two primary roles: (i) maintaining tissue integrity to prevent microbial invasion, which needs cell adhesion, proliferation, and migration, and (ii) constituting the immune system’s first line of defense by secreting inflammatory mediators. PRRs potentially regulate all these functions. Intestinal epithelial cells express PRRs that specifically detect molecular patterns from both commensal and pathogenic gut microbes, a topic that has been extensively reviewed. A study conducted in mice revealed that Amuc_1100, a membrane protein derived from the commensal bacterium *Akkermansia* muciniphila, interacts with TLR2 in intestinal epithelial cells, thereby enhancing intestinal barrier function, including mucus thickness and tight junction protein (TJP) expression.^167,168^

ECs, similar to epithelial cells, exhibit context-dependent immune functions mediated by PRRs. During infections, ECs recognize microbial patterns and respond to various endogenous stimuli, including DAMPs and cytokines, primarily through PRRs, notably TLRs.^169,170^ The expression of TLRs in ECs can vary across different tissues. For instance, human umbilical cord ECs exhibit low levels of TLR5-10 but high levels of TLR1-4, whereas human aortic ECs show elevated expression of all TLRs except for TLR9 and TLR3, in contrast to human peripheral blood mononuclear cells.^171^ This variability in TLR expression may also exist among different segments of the vascular tree, with major blood vessels displaying low endothelial expression of TLR4 and TLR2.^170^ In addition to mature ECs, TLRs are identified in precursor ECs, such as endothelial colony-forming cells, which hold promise for tissue repair and postnatal vascularization strategies.^172^ Furthermore, TLRs play a crucial role in cell reprogramming and differentiation. Activation of TLR3 by its ligand, dsRNA, in combination with endothelial growth factors, induces the differentiation of human fibroblasts into ECs.^173^

Overall, PRRs in non-immune cells remain a fertile area for research, with numerous unanswered questions about their regulation, context-dependent functions, and contributions to health and disease.

### Immune cells

As the evolutionarily developed first defense, the innate immune system provides dual-level protection against pathogens. (1) Barrier defenses: Physical (skin, mucosa) and chemical barriers form the initial protection layer, supplemented by specialized structures like the blood-brain barrier. In addition, chemical defenses, including fatty acids, low pH, enzymes, and components of the complement system, contribute to pathogen exclusion. (2) Cellular defenses: The second level of innate immunity involves nonspecific immune surveillance and defense mechanisms mediated by innate immune cells in vertebrates. These innate immune cells include monocytes, neutrophils, macrophages, DCs, NK cells, mast cells, eosinophils, and basophils. Innate immune cells detect conserved pathogens and damage patterns via PRRs, unlike adaptive immune cells that use highly specific antigen receptors. Through the recognition and binding of these patterns, PRRs are crucial for initiating and modulating immune responses. They elicit immune-protective effects, including anti-infection and antitumor activities, and serve as crucial mediators in the activation of specific immune responses. This dual-level organization underscores the versatility and evolutionary significance of innate immunity in maintaining organismal health.

**Fig. 6:**
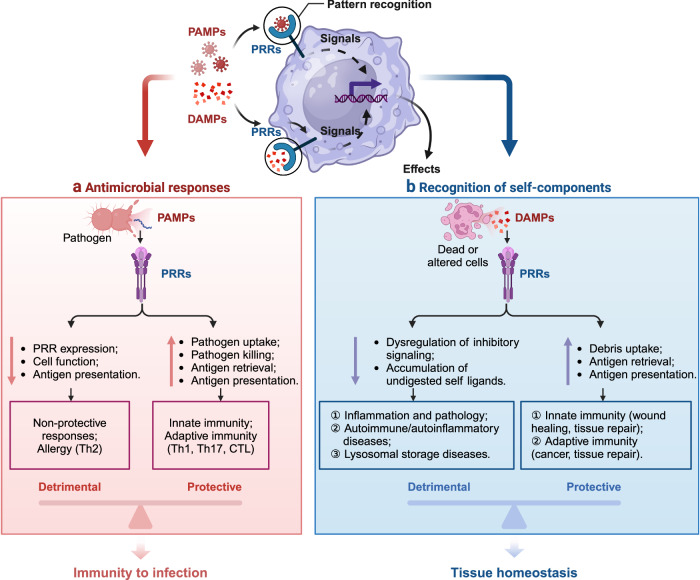
Double-edged sword effect of PRR in vivo. **a** The double effects of PRRs in antimicrobial responses. The role of PRRs in immunity to infection is complex. The functions of PRRs can be detrimental; for example, they can alter the expression of other key PRRs, inhibit antigen presentation, facilitate viral infection of myeloid cells, and promote pathological inflammation, including Th2 responses in the context of allergies. In addition, PRRs play a vital role in immune defense by recognizing PAMPs on the cell surface, inducing intracellular signaling cascades that mediate pathogen uptake and killing, production of inflammatory mediators, and the induction of several other protective cellular responses, such as the production of ROS and NET formation. Also, PRRs can promote antigen presentation and shape the development of adaptive immunity, including Th1, Th17, and CTL responses to provide protection. **b** The double effects of PRRs in recognition of self-components. PRRs serve a critical function in the immune response by enhancing the processing and presentation of antigens derived from necrotic cells, which is pivotal for the evolution of adaptive immune responses, notably those directed against neoplastic entities. Inflammation and adaptive immunity induced by self-recognition of PRRs can lead to additional beneficial outcomes, such as wound healing or tissue repair. Activation signals from PRRs and other receptors are balanced by inhibitory signals from inhibitory PRRs to prevent detrimental responses. However, dysregulated, excessive, or persistent activation signals due to dysregulation of inhibitory signaling and accumulation of undigested self-ligands can lead to immunopathology, including autoimmune and/or autoinflammatory diseases. This figure is adapted from Reis et al.^78^ with some modifications. This figure is created by BioRender (https://app.biorender.com)

The double-edged sword effect of PRRs is reflected in the antimicrobial response and recognition of self-components (Fig. 6). A recent review offers a comprehensive description of the current landscape, underscoring key findings and concepts that elucidate how the innate immune system, predominantly through the actions of PRRs expressed by DCs, governs and molds the activation of the adaptive immune system.^174^ Furthermore, PRR signaling can also occasionally operate directly within lymphocytes, impacting T-cell and B-cell activation and differentiation. While the activation of T and B lymphocytes is primarily fueled by the engagement of their specific antigen receptors (T-cell receptors (TCRs) and B-cell receptors (BCRs)), a substantial body of evidence suggests that PRR activation, particularly that of TLRs, can either directly or synergistically interact with TCRs or BCRs to modulate lymphocyte responses. Compelling evidence for direct PRR activation is derived from studies on B cells, which express multiple TLRs. For instance, in mice, stimulation with LPS, a ligand for TLR4, triggers signaling through two distinct pathways: one via the BCR, leading to the activation of spleen-associated tyrosine kinase (SYK), extracellular signal-regulated kinase (ERK), and AKT serine/threonine kinase (AKT), and the other through MYD88, culminating in NF-κB activation. These pathways jointly regulate B-cell survival, proliferation, and cytokine secretion.^175^ Both murine and human T cells express TLRs, as evidenced by messenger RNA (mRNA) expression, and both CD4^+^ and CD8^+^ T cells exhibit responses to specific TLR activations.^176^

Thus, immune cells have different PRRs to enable the immune system to detect and respond to a wide variety of pathogens and endogenous danger signals effectively. This diversity is essential for the immune system’s ability to provide broad protection while tailoring responses to specific challenges.

## PRRs and their ligands

Although their roles are multifaceted, PAMPs and DAMPs primarily act as “Signal 0,” engaging specific receptors, predominantly pattern recognition receptors (PRRs), to activate immune responses and regulate other cellular activities, such as autophagy, apoptosis, and tissue repair.^38^

### PAMPs: the exogenous signals

In 1989, Charles Janeway introduced a revolutionary theory emphasizing that the immune system’s primary function is not to respond to harmless foreign antigens but to defend the host against infectious pathogens. He hypothesized that receptors on APCs of the innate immune system recognize specific signals, which he initially termed “Signal 0 s” and are now referred to as PAMPs.

**Fig. 7:**
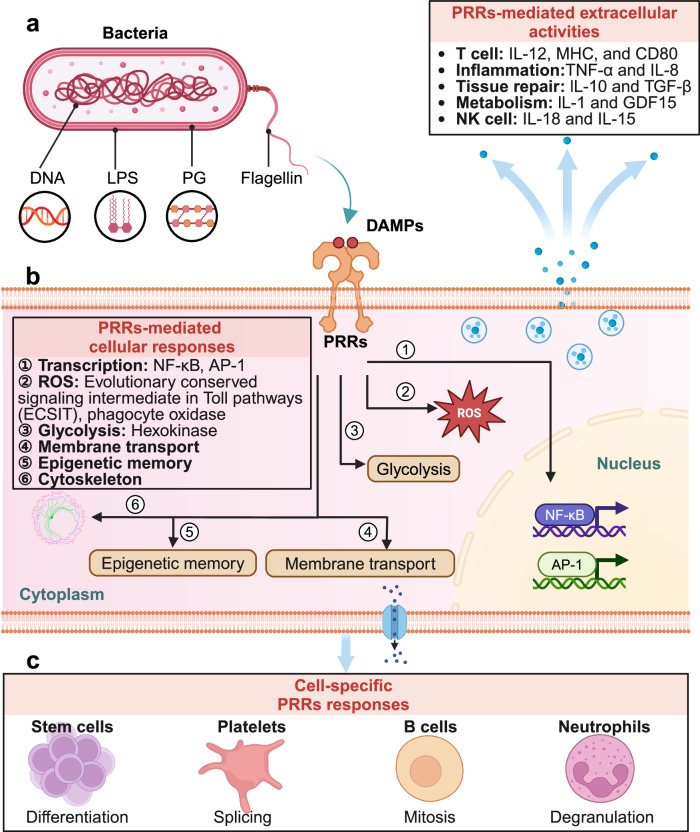
Cellular responses induced by PRRs in microbial infection. **a** PRRs-mediated extracellular activities. Cell-extrinsic responses triggered by PRRs signaling encompass a range of activities that impact the immediate (or systemic) milieu surrounding the cell responsible for the detection of PAMPs. **b** PRRs-mediated cellular responses. Cell-intrinsic responses induced by PRR signaling occur within the cell that detected a PAMP and contribute to the activities indicated in (**a**). **c** Cell-specific PRRs responses. Cell-type-specific responses induced by PRR signaling occur uniquely within the indicated cell type and are mediated by TLR–PAMP interactions. This figure is created by BioRender (https://app.biorender.com)

When questioned by Dr. Michael T. Lotze about the role of these signals in sterile inflammation, including cancer, Janeway acknowledged the importance of the issue but admitted that the connection was not yet fully understood at the time. PAMPs are unique molecular signatures of microorganisms, highly specific and evolutionarily conserved, allowing host cells to distinguish between “self” and “non-self” (a concept known as the “stranger hypothesis”). This recognition drives innate immune responses, forming the foundation for effective host defense mechanisms.^177^ These patterns encompass a wide array of microbial structures that are crucial for pathogen survival but are absent or significantly different in the host. PRRs initiate diverse cellular responses upon the detection of microbes (Fig. 7).

PAMPs can be broadly categorized into several groups: microbial surface components such as peptidoglycans and LPS from bacterial cell walls, mannans and β-glucans from fungal cell walls, and flagellin, the protein component of bacterial flagella. Nucleic acids, including bacterial and viral DNA/RNA, particularly unmethylated CpG DNA motifs, dsRNA, single-stranded RNA (ssRNA), and 5′-triphosphate RNA, are also part of PAMPs. Z-DNA represents an alternative conformation of dsDNA, differing markedly from the conventional B-DNA structure.^178^ Similarly, dsRNA can adopt a Z-conformation.^179^ Z-DNA-binding protein 1 (ZBP1), a cytosolic PRR, is indispensable for the detection of Z-nucleic acids and the initiation of subsequent immune signaling pathways. ZBP1 is central to various immune processes, including virus-induced inflammation,^180^ NLRP3 inflammasome activation,^181^ and cell death regulation.^182^ It also stabilizes Z-conformation mitochondrial DNA (mtDNA) and forms a cytosolic complex with cGAS, RIPK1, and RIPK3, which facilitates sustained signal transducer and activator of transcription 1 (STAT1) phosphorylation and IFN-I signaling.^183^ Strikingly, mice deficient in ZBP1 or IFN-I signaling exhibit resistance to Doxorubicin-induced cardiotoxicity, positioning ZBP1 as a potential therapeutic target in heart failure where mtDNA stress initiates IFN-related pathology.^183^

In addition, the Zα domain of adenosine deaminase acting on RNA-1 (ADAR1) is involved in adenosine-to-inosine (A-to-I) editing of endogenous Alu elements, preventing dsRNA formation from inverted Alu repeats.^184^ This editing is crucial for averting pathogenic IFN-I responses triggered by Z-RNA-dependent ZBP1 activation.^185^ A deficiency or malfunction of ADAR1 leads to an accumulation of unedited dsRNA, contributing to autoinflammatory conditions such as Aicardi–Goutières syndrome and dyschromatosis symmetrica hereditarian.^186^ Metabolic byproducts, such as formylated peptides, small protein fragments unique to bacteria, and metabolites like UA released during microbial growth, fall under this category. In addition, other molecules such as membrane components [peptidoglycans, lipoteichoic acid, and glycosylphosphatidylinositol (GPI)] are also considered PAMPs^187^ (Fig. 5).

With the progressively deeper understanding of PAMPs stemmed from microorganisms such as bacteria, fungi, and viruses, evidence pointing to the existence of PAMPs in parasites has started to emerge.^188^ In contrast to PAMPs from classical microbial pathogens, which primarily originate from cell walls and specific nucleic acids, parasite PAMPs exhibit a broader range of sources and more complex structures. These PAMP components are present in all stages of parasitic organisms within their hosts. Parasite PAMPs differentiate from traditional PAMPs in terms of molecular structure, receptor binding mechanisms, intracellular signal transduction pathways, and the effects they induce. Several studies have demonstrated that excretory-secretory products of parasites can elicit inflammatory responses in their hosts, including species such as *Acanthocheilonema viteae, Clonorchis sinensis, and Anisakis simplex*.^189^ Besides excretory-secretory products, adult parasites and their eggs are also significant sources of PAMPs. Research has shown that the lipid molecule lysophosphatidylserine (lyso-PS), containing a single acyl group and found in adult Schistosoma mansoni and/or its eggs, can potently activate TLR2 signaling. Lyso-PS stimulates human embryonic kidney (HEK) 293 CD14/TLR2 cells to produce substantial amounts of IL-8, while phosphatidylserine (PS) components with diacyl groups do not show such activity^190,191^ (Fig. 5). Furthermore, the GPI of protozoa can also activate TLR2 signaling and induce pro-inflammatory cytokine responses (Fig. 5). The intensity of this response is directly correlated with the lipid and sugar content of GPI. GPI components have been identified in the merozoites of Plasmodium falciparum.^192^ These components are capable of activating murine peritoneal macrophages via TLR2/TLR1 or TLR2/TLR6, thereby stimulating the release of tumor necrosis factor-α (TNF-α) and IL-6.^193^

In mammals, PAMPs are recognized both extracellularly and intracellularly^194^; however, in plants, their detection mainly takes place at the cell surface.^195^ The recognition of most PAMPs by TLRs often necessitates the assistance of molecular companions, such as lymphocyte antigen 96 (LY96, also known as MD-2), CD14, MRs, dectins, and collectin receptors. For instance, the successful binding of LPS to TLR4 needs additional molecules, such as lipopolysaccharide binding protein (LBP), CD14, and LY96.^196^ Similarly, the activation of TLR2 signaling by Gram-positive (G^+^) and Gram-negative (G^-^) bacteria, as well as their LPS, peptidoglycan, and lipoteichoic acids, also depends on LY96. Upon recognizing PAMPs, activated TLRs and other PRRs found on the cell surface, in the cytoplasm, or within intracellular vesicles signal the host to indicate a microbial infection. This triggers pro-inflammatory and antimicrobial responses by activating various intracellular signaling pathways. These pathways involve adapter molecules, kinases, and transcription factors (TFs), including NF-κB, JunD proto-oncogene, AP-1 transcription factor subunit (JUND, also known as activator protein 1 [AP-1]), and IRFs. PAMP-induced signal transduction pathways culminate in the activation of gene expression and the production of various effector molecules, including cytokines, chemokines, cell adhesion molecules, and immunoreceptors. These molecules orchestrate the innate and adaptive immune responses by detecting and responding to microorganisms. This interaction plays a pivotal role in mounting a rapid and nonspecific defense against a wide range of pathogens, including bacteria, viruses, fungi, and parasites, thereby ensuring effective host protection.

### DAMPs: the endogenous signals

In 1994, Polly Matzinger introduced a fundamental theory model proposing that the immune system prioritizes the recognition of ‘danger’ or ‘damage’ over the distinction between self and non-self.^6^ This model raises the notion that the immune system defines danger as any factor causing tissue stress or destruction. According to this framework, APCs are activated by either PAMPs from microbes or DAMPs released by stressed or damaged tissues. Matzinger’s danger model provides a compelling explanation for the robust immune responses observed in sterile inflammation, shedding light on the immune system’s ability to respond to tissue damage even in the absence of microbial pathogens.

Over the past two decades, numerous endogenous molecules have been identified as DAMPs, which are released or activated in response to cellular stress or injury and subsequently trigger inflammatory and immune responses.^197^ These molecules undergo conversion into DAMPs through mechanisms such as displacement, modification of properties, or alterations in concentration levels. With the expanding array of DAMPs, they can be categorized into various groups based on their chemical nature or subcellular localization. These categories include nucleic acids, proteins, lipids, carbohydrates, ions, metabolic byproducts, mitochondrial components, extracellular matrix (ECM) constituents (such as hyaluronic acid, heparan sulfate, matrilin-2 [MATN2], fibrinogen, hyaluronan, fibronectin, and enolase 1 [ENO1]), plasma components including complement (C3a, C4a, and C5a), intracellular molecules residing in various cellular regions such as cytosol, mitochondria, endoplasmic reticulum (ER), nucleus, and lysosome (including ATP, UA, and nucleotides), cell wall components (including peptidoglycan and β-glucan), as well as cytokines and chemokines^197^ (Fig. 5).

The localization and function of DAMPs are highly dynamic, shaped by diverse stimuli and contextual factors. As such, their release and function are tightly regulated by the cellular environment. Several comprehensive reviews, including our own and others’, have traced the evolution of the DAMP concept. These reviews provide detailed insights into the classification, biogenesis, signaling pathways, and clinical implications of DAMPs.^198–200^

## PRR signaling pathways

**Fig. 8:**
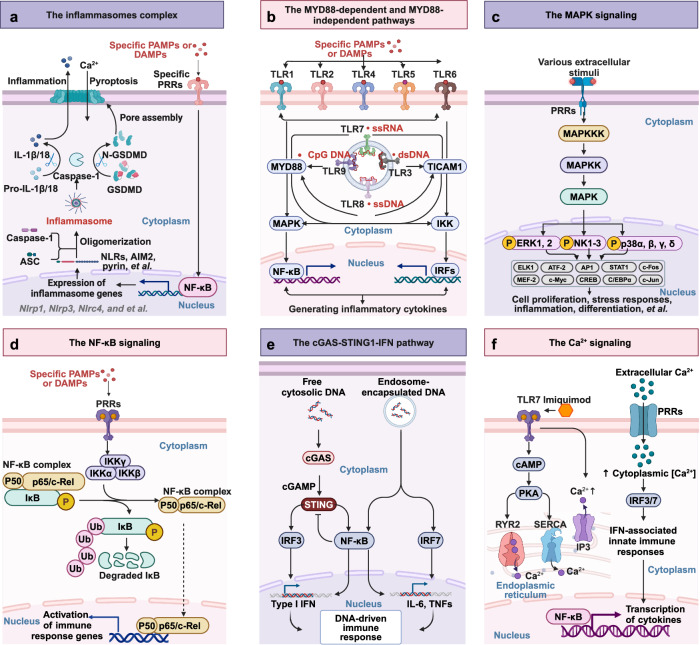
Distinct signaling pathways in the recognition of PAMPs and DAMPs by PRRs. **a** Inflammasomes complex. Recognition of PAMPs or DAMPs by PRRs stimulates the NF-κB-mediated upregulation of inactive pro-IL-1β and multiple receptor proteins, such as NLRP1, NLRP3, NLRC4, NLRC12, as well as the proteins AIM2 and pyrin. The downstream signaling involves the engagement of a receptor protein activator, which drives the assembly of the inflammasome, proximity-induced autoproteolysis of caspase-1, and the subsequent cleavage of IL-1β, IL-18, and GSDMD, ultimately leading to the induction of inflammation and pyroptosis. **b** MYD88-dependent and MYD88-independent pathways. TLRs are universally expressed across diverse immune system cells, with TLRs 1, 2, 4, 5, and 6 positioned prominently on the cell surface, while TLRs 3, 7, 8, and 9 are intimately associated with endosomal membranes. Upon recognition of various DAMPs or PAMPs by these TLRs, they initiate a complex signaling cascade, leveraging adaptor proteins MYD88 (MYD88-dependent pathways) and TRIF (MYD88-independent pathways) as crucial intermediates. These adaptors, in turn, activate downstream MAPKs and IKK, which collaborate to facilitate the production of inflammatory cytokines by activating transcription factors like AP-1 and NF-κB, respectively. **c** The mitogen-activated protein kinase (MAPK) signaling. Typical MAPKs activated by various extracellular stimuli are characterized by a three-tiered signaling cascade, starting with MAP3KK, followed by MAP2K, and culminating in the activation of MAPK. In mammalian cells, three major MAPK families have been identified: ERK1/2, JNK1/2/3, and the p38 (α, β, γ, δ) isoforms signaling cascades. The transcription factors regulated by MAPK signaling include ELK1, ATF-2, AP1, STAT1, c-Fos, MEF-2, c-Myc, CREB, C/EBPα, and c-Jun. The MAPK pathway precisely influences cell proliferation, stress responses, inflammation, differentiation, functional synchronization, transformation, and apoptosis. **d** The NF-κB signaling. Upon the recognition of PAMPs or DAMPs by PRRs, a critical signaling cascade is initiated. This leads to the activation of the IκB Kinase (IKK) complex, which is composed of IKKα, IKKβ, and NEMO (also known as IKKγ). The IKK complex then phosphorylates IκB (inhibitor of κB), a protein that keeps the NF-κB complex (made up of p50 and p65 subunits) inactive in the cytoplasm. Following phosphorylation, IκB is marked for ubiquitination (Ub) and is subsequently degraded, allowing the NF-κB complex to be released. The liberated NF-κB complex translocates to the nucleus, where it triggers the transcription of genes involved in immune responses. This signaling pathway is essential for a swift reaction to infections and inflammatory stimuli, and it also plays a pivotal role in governing cell survival and proliferation. **e** The cGAS–STING–IFN pathway. In the cytoplasm, the presence of free cytosolic DNA or endosome-encapsulated DNA triggers the activation of the cGAS enzyme. cGAS synthesizes cGAMP, which serves as a second messenger that binds to and activates STING. This activation initiates a signaling cascade that involves the transcription factors IRF3 and IRF7, leading to the production of Type I IFNs. Concurrently, STING also activates the NF-κB pathway, which promotes the expression of inflammatory cytokines such as IL-6 and TNF. The coordination of these signaling events within the nucleus results in a robust DNA-driven immune response, which is essential for the defense against intracellular pathogens and the regulation of immune homeostasis. **f** The Ca^2+^ signaling. Extracellular Ca^2+^ signaling mediated by ion channels in the cell membrane can modulate the activation of these PRR subfamilies. This modulation favors the promotion of IRF3/7 activation, initiating IFN-associated innate immune responses and enhancing NF-κB-related inflammatory reactions. Furthermore, recent evidence has revealed that TLR7 augments the cAMP-PKA pathway, ultimately leading to increased expression of SERCA and RYR2 in the sarcoplasmic reticulum, thereby disrupting Ca^2+^ signaling within cardiomyocytes. This figure is created by BioRender (https://app.biorender.com)

PRRs’ recognition of PAMPs and DAMPs causes the upregulation of distinct signaling pathways and genes in terms of transcription. This upregulation is dependent on factors such as the specific PRRs involved, cell types, genetic determinants, individual variations, and the different contexts, including the extracellular microenvironment and intracellular homeostasis. The variation in signaling cascades triggered by individual PRRs is partly due to three main classes of molecules involved in signal transduction: protein kinases, adapter proteins, and transcription factors. Although PRRs are activated by their specific ligands in different subcellular compartments through distinct mechanisms, the core signaling molecules exhibit similar structures and functions. Their signals interweave through crosstalk, often converging into several common and pivotal signaling pathways (Fig. 8). These pathways are interconnected in regulating the many physiological and pathological processes they affect. Rather, they may interact directly or indirectly with other molecules, subsequently triggering interactions with additional signaling pathways.

### Inflammasomes complex

The term “inflammasome” was first introduced by Tschopp and colleagues in 2002 to describe a high-molecular-weight complex present in the cytosol of activated immune cells, playing a key role in orchestrating the activation of inflammatory caspases^201^ (Fig. 1e). Following this pivotal report, the field has witnessed substantial growth, leading to the identification of multiple distinct inflammasomes. Each inflammasome assembles in response to specific PAMPs or DAMPs within the host cell cytosol, guided by unique PRRs. Upon recognition of an inflammatory ligand, the sensor becomes activated and undergoes oligomerization. This process subsequently recruits an adaptor protein known as apoptosis-associated speck-like protein (ASC). ASC is characterized by two death-fold domains: a PYD and a CARD. These domains enable ASC to act as a bridge, connecting the upstream inflammasome sensor to the downstream inflammatory caspases, thereby facilitating their auto-processing and initiating subsequent inflammatory responses.^202^

To date, multiple receptor proteins have been validated as integral components in the assembly of inflammasomes. These include members of the NLR family, such as NLRP1, NLRP3, NLRC4, and NLRC12, as well as the proteins AIM2 and pyrin, all encoded within the human and mouse genomes. In addition, there are potentially other less-characterized pathways involving NLRP6, NLRP7, NLRP12, RIG-I, and IFI16. These proteins have been reported to facilitate caspase-1 activation, which in turn processes pro-IL-1β/pro-IL-18 into their respective mature cytokines.^203,204^ Recently, in response to specific ligands, including PAMPs/DAMPs (such as heme) and DAMP/cytokine combinations, NLRC5 associates with NLRP12 and cell death molecules to form an NLRC5-PANoptosome complex. This complex, comprising NLRC5, ASC, NLRP12, NLRP3, RIPK3, and caspase 8, triggers inflammatory cell death (PANoptosis [pyroptosis, apoptosis, necroptosis]) and the release of cytokines and DAMPs in a manner dependent on TLR2, TLR4, and nicotinamide adenine dinucleotide (NAD^+^).^205^ In addition, the canonical caspase-1 inflammasome is complemented by noncanonical inflammasome activation, which relies on caspase-11 in mice and caspase-4 and caspase-5 in humans.^206,207^

Recent studies have not only elucidated the core components of inflammasomes but also uncovered a variety of regulatory mechanisms. These include transcriptional and post-transcriptional regulation, post-translational modifications (PTMs), and the involvement of various proteins that modulate inflammasome activity at the levels of receptors, the adaptor protein ASC, or caspases. In the following discussion, we focus on endogenous PRR-related regulatory mechanisms that directly influence ligand recognition, complex assembly, and PTMs.

The NLRP3 inflammasome is recognized as the most notable inflammasome, serving a critical function in promoting both innate and adaptive immune reactions.^208^ Its activation typically occurs in a “two-step” process. Initially, the priming signal stimulates the NF-κB-driven enhancement of inactive pro-IL-1β and NLRP3 (or, in certain instances, non-transcriptional priming).^120^ This step also includes PTMs, including ubiquitylation, phosphorylation, small ubiquitin-like modifier (SUMO) modification (SUMOylation), neddylation, acetylation, ADP-ribosylation, and nitrosylation.^209–216^ The second signal triggers NLRP3 activation, leading to the assembly of the inflammasome, proximity-induced autoproteolysis of caspase-1, and the subsequent cleavage of IL-1β, IL-18, and GSDMD. This process ultimately results in the release of mature active cytokines and the initiation of pyroptosis, an inflammatory form of programmed cell death^217^ (Fig. 8a). The role of priming—engagement of PRRs or cytokine receptors—in the activation mechanism of NLRP3 is well-established. Initially, priming was thought to solely upregulate NLRP3 expression transcriptionally. However, recent studies have revealed that it also facilitates inflammasome signaling by triggering the Lys63-specific deubiquitylation of NLRP3 by BRCA1/BRCA2-containing complex subunit 3 (BRCC3). This essential step for NLRP3 activation requires the production of reactive oxygen species and occurs solely following TLR stimulation.^209,210^ In resting macrophages, NLRP3 protein levels are low, serving as a bottleneck for inflammasome formation. To support inflammasome formation, NLRP3 must be induced to reach a threshold concentration. Cells acquire this priming signal from signaling receptors that activate NF-κB, with TLRs being the most extensively studied. TLR signaling leads to new transcription of NLRP3 and synthesis of pro-IL-1β.^218^ The transcription of NLRP3 is dependent on NF-κB activation. Simultaneous engagement of TLRs and NLRP3 can also assemble the NLRP3 inflammasome without requiring new protein synthesis. This rapid response pathway relies on interleukin-1 receptor-associated kinase 1 (IRAK1), which directly links TLR ligation to swift NLRP3 inflammasome assembly.^219^ TLRs also modulate inflammasome activation through PTMs such as ubiquitination and phosphorylation.^220,221^ NLRP3 can be phosphorylated on serine and tyrosine residues. Phosphorylation at S3 inhibits NLRP3 activation, whereas phosphorylation at S194, mediated by JUN N-terminal kinase 1 (JNK1), promotes it. In addition, NLRP3 is phosphorylated on tyrosine residues, and dephosphorylation by the protein tyrosine phosphatase protein tyrosine phosphatase non-receptor type 22 (PTPN22) enables NLRP3 activation.^222^ Furthermore, TLR priming induces NLRP3 ISGylation, a PTM where ISG15 ubiquitin-like modifier (ISG15) covalently binds to the target protein, stabilizing the NLRP3 protein.^223^

PRRs also exert an influence on noncanonical inflammasome activation.^224^ In *Tlr4*-deficient mice, the noncanonical pathway directly sets off the activation of caspase-11 in mouse cells and caspase-4 and -5 in human cells upon LPS exposure.^225,226^ TLR4 is needed for priming this response.^227^ Signaling through TLR via TICAM1 stimulates the amount of caspase-11 produced, which subsequently leads to the proteolysis of GSDMD, enabling the release of IL-1 and inducing pyroptotic cell death.^228^ Of importance, when caspase-1 is absent, caspase-11 heightens vulnerability to *Salmonella* infection.^228^ Collectively, these findings suggest that PRRs regulate the activity of NLRP3 and other inflammasomes by serving as priming, licensing, and limiting signals, thereby modulating the activation and function of these pathways.

### MYD88-dependent and TRIF-dependent pathways

Upon recognizing its specific ligand through interactions with the LRR region, a single TLR differentially recruits TIR domain-containing adapters, initiating diverse signaling cascades. The TIR domain-containing adapters include five proteins: MYD88, TIR domain-containing adapter inducing IFN-β (TRIF, also termed TICAM1), TIR domain-containing adapter protein (TIRAP, also known as Mal), TIR domain-containing adaptor molecule 2 (TICAM2, also known as TRIF-related adapter molecule [TRAM]), and sterile alpha and TIR motif containing protein (SARM, also known as sterile alpha and armadillo motif containing protein). In summary, the adapter molecules involved in TLR signaling allow it to be broadly grouped into two distinct pathways: the MYD88-dependent pathway and the MYD88-independent pathway, which is also referred to as the TRIF-dependent pathway (Fig. 8b).

MYD88 is essential for downstream signaling in numerous TLRs, significantly excluding TLR3.^229^ Structurally, MYD88 consists of both a death domain and a TIR domain. It binds to IRAK4, a serine/threonine kinase that has an N-terminal death domain. IRAK4 subsequently activates other members of the IRAK family, namely IRAK1 and IRAK2. IRAK4 is the initial enzyme recruited to the Myddosome complex, facilitated by either TICAM1/ TICAM2 or MYD88/TIRAP.^230^ TIRAP/Mal is necessary to bridge the interaction between the TLR and MYD88 in TLR2 and TLR4 signaling. In addition, TNF receptor-associated factor 6 (TRAF6), known to stimulate the NF-κB pathway, a member of the TRAF family interacts with IRAK1.^231^ TRAF proteins predominantly facilitate inflammatory responses on the cell surface and in intracellular PRR signaling pathways, specifically propelling IFN-I responses.^232^ TRAF6 has been shown to function downstream of the NF-κB, mitogen-activated protein kinase 14 (MAPK14, also known as p38 or p38 MAPK), and JNK signaling cascades.^233,234^ To dynamically study endogenous TLR-mediated, MYD88-dependent pathways, researchers leveraged engineered macrophages to carry out advanced microscopic examination and proteomic analysis of the myddosome complex. Their findings revealed that MYD88 assembles into barrel-like structures, which operate as scaffolds for the assembly of effector proteins in the majority of myddosomes. Further proteomic analysis revealed that myddosomes include proteins participating in every stage of the TLR pathway and regulate diverse effector responses. Genetic studies provided additional insights by delineating the epistatic interactions among these effector modules. Together, these findings highlight that the complex cellular responses of the TLR pathway are intricately coordinated and executed within the structural framework of myddosomes.^39^

Searches through meta databases resulted in the identification of TICAM2.^235^ Analogous to the TIRAP and MYD88 pairing, TICAM2 is essential for the engagement of the TICAM1 adaptor.^236^ Unlike TIRAP, TICAM2 features myristoylation at its N-terminus, enabling its anchoring to the endosomal membrane.^237^ Mutations that eliminate TICAM2 myristoylation impair its cytoplasmic localization, thereby blocking TICAM1-mediated signal transduction via TLR4.^237^ Furthermore, the signaling lymphocytic activation molecule family member 1 (SLAMF1) has been shown to positively modulate signaling downstream of TLR4. The interaction interface between SLAMF1 and TICAM2 has been delineated, which subsequently prevents mortality in mice subjected to LPS shock.^238^

In response to dsRNA stimulation, TLR3 recruits the adaptor protein TICAM1. Meanwhile, TLR4 activates both MYD88-dependent and TICAM1-dependent signaling pathways, with TICAM2 being a necessary component. This activation leads to the induction of IRF3 and NF-κB transcription factors, resulting in the expression of genes encoding inflammatory cytokines and IFNs.^239^ TICAM1, through a TRAF-binding motif in its N-terminal region, interacts with TRAF3 and TRAF6, initiating alternative pathways that culminate in the activation of IRF3, NF-κB, and mitogen-activated protein kinase (MAPK, also known as MPK).^240^ In addition, TICAM1 contains a C-terminal receptor-interacting protein (RIP) homotypic interaction motif and interacts with TRAF6 and RIP1, which are responsible for NF-κB activation.^241^ Evidence also suggests that TICAM1 plays a role in cell death and inflammation independently of TLR3 or TLR4 engagement. Specifically, in response to LPS or Yersinia pseudotuberculosis, TNF-induced cell death and inflammation in murine macrophages were directly mediated by the formation of pro-death and pro-inflammatory complexes involving TICAM1 and the LPS co-receptor CD14. This highlights an important function for these proteins beyond TLR-mediated immune responses.^242^ These findings underscore the complex and multifaceted roles of TICAM1 in innate immune signaling and inflammation.

### The MAPK signaling

MAPKs are a family of serine/threonine protein kinases triggered by a wide range of stimuli, including cytokines, neurotransmitters, hormones, cellular stress, and cell adhesion signals. The MAPK pathway acts as a highly sensitive sensor of extracellular and intracellular fluctuations, regulating diverse cellular processes. These effects extend beyond the cellular level, influencing organ function and even organismal homeostasis.^243^ These changes are transmitted by this signaling pathway from the cell surface to the nucleus, resulting in a diverse array of cellular responses. In mammalian cells, three major MAPK families have been identified: ERK1/2, JNK1/2/3, and the p38 (α, β, γ, δ) isoforms signaling cascades.^244^ Typical MAPKs are defined by a three-tiered signaling cascade. In this process, a stimulus induces the phosphorylation and activation of a Ser/Thr MAPK kinase kinase, which then phosphorylates and activates MAPK kinases. The last step of this cascade is the unique dual phosphorylation of a specific tripeptide motif (Thr-X-Tyr) within the MAP kinase domain activation loop by MAPK kinase, leading to a conformational change in the activation loop and enabling the kinase active site to interact with and activate MAPK^245^ (Fig. 8c).

The activation of MAPKs in innate immune cells like macrophages and DCs, which lies downstream of PRRs, has been the subject of extensive investigation. PRRs are tasked with recognizing a wide range of specific DAMPs and PAMPs from both the cell surface and endosomal membranes. For instance, in the MYD88-dependent TLR pathway, IRAK1 undergoes phosphorylation and interacts with TRAF6, leading not only to the activation of the NF-κB inhibitor (IκB) kinase (IKK) complex but also to the stimulation of MAPKs such as JNK and p38 MAPK.^246^ Engagement of the DC-asialoglycoprotein receptor (DC-ASGPR) by a specific monoclonal antibody (mAb) (clone 49C11), a CLR expressed on human DCs, rapidly triggers the activation of MAPK ERK1/2 and JNK, followed by the release of IL-10. This suggests an immunomodulatory role for MAPK signaling pathways mediated through PRRs.^247^ MAPKs also participate in the coordinated signaling regulation among different PRRs. For example, in chronic granulomatous disease with fungal infections, the Dectin-1 C-type lectin receptor, which recognizes β-glucans, and TLRs mediate cytokine responses in wild-type murine neutrophils through the activation of tyrosine phosphatase PTPN11-SYK and downstream caspase recruitment domain family member 9 (CARD9)-dependent NF-κB as well as CARD9-independent JNK-c-Jun pathways.^248^ Furthermore, the invasion of cells by bacterial components leads to NLR activation, recruitment of downstream CARD9, and subsequent activation of p38, JNK, and the MAPK pathway, thereby stimulating the release of pro-inflammatory factors.^249^

### The NF-κB signaling

In 1986, Ranjan Sen and David Baltimore discovered a nuclear factor in B lymphocytes that can bind to the κ enhancer of the immunoglobulin κ light-chain gene. This discovery was made using electrophoretic mobility shift assays with terminally tagged DNA fragments. They named this factor nuclear Factor κ-light-chain-enhancer of activated B cells (NF-κB), highlighting its role in regulating κ light-chain gene expression in B cells.^250,251^ Five members make up the mammalian NF-κB transcription factor family: nuclear factor kappa-B subunit 1 (NFKB1, also known as NF-κB1 or p105 or p50), NFKB2 (also known as NF-κB2 or p100 or p52), RELA proto-oncogene, NF-κB subunit (RELA), RELB, and REL proto-oncogene, NF-κB subunit (REL, also known as c-Rel). Owing to the shared conserved Rel homology domain, any two of these family members can form homo- or heterodimers. Under normal conditions, these dimers bind to IκB and remain sequestered in the cytoplasm in an inactive state. The most common dimerization form is RELA/NFKB1.^252^ Upon phosphorylation of IκBa, it undergoes degradation via the ubiquitin-proteasome system. This process releases the NF-κB heterodimer, enabling it to move into the nucleus and trigger the expression of pro-inflammatory cytokine genes (Fig. 8d).

There is plenty of evidence showing that NF-κB is crucial for inflammation and immune responses, which underlines its key role in various biological processes. As a central regulatory hub, NF-κB signaling intersects with various pathways essential for the activation of both immune and non-immune cells. These pathways include MAPK, cGAS–STING, phosphatidylinositol 3-kinase (PI3K)/AKT, Janus kinase (JAK)/ STAT, Wnt, transforming growth factor-beta (TGF-β), Notch, and Hedgehog signaling, as well as signal transduction mediated by PRRs in innate immunity.

For example, TLRs act as sensors for PAMPs and DAMPs, detecting potential infections and initiating immune reaction aimed at protecting the host. TLR signaling is mediated through the N-terminus of MYD88, which activates the TAK1–TAB1–TAB2/3 complex, which brings about the phosphorylation and activation of the IKK complex. This ultimately promotes the relocation of NF-κB to the nucleus, triggering the transcription of genes that promote inflammation. However, TLR engagement does not always result in NF-κB activation, as it signaling can be negatively modulated by other molecular mechanisms or pathways to prevent excessive inflammation. For instance, in macrophages, LPS stimulation can suppress NF-κB signaling through the induction of the inducible cAMP early repressor (ICER). This suppression is mediated by p38 MAPK-activated cAMP response element-binding protein (CREB), which forms a negative feedback loop to attenuate TLR-induced excessive inflammatory responses. This regulatory mechanism is essential for keeping immune homeostasis and stopping harmful inflammation.^253^

The signaling pathway from NLRs to NF-κB is believed to include the oligomerization of RIPK2, which then moves to activate the IKK complex. Specifically, upon bacterial invasion, NOD1 and NOD2 recognize the bacterial components γ-D-Glu-meso-diaminopimelic acid (iE-DAP) and muramyl dipeptide, respectively.^254^ This recognition triggers the activation of NLRs, resulting in their self-dimerization and the recruitment of downstream RIPK2 through its CARD domain.^255^ Once activated, RIPK2 assembles a complex with MAP3K7, TAB1, and the complex of NF-κB essential modulator, IKKα and IKKβ. This assembly results in the activation of IKKα/IKKβ, which subsequently promotes the transcription of NF-κB and stimulates the production and release of pro-inflammatory factors.^256^ In models of acute kidney injury induced by LPS and kidney ischemia/reperfusion (I/R) injury, peroxiredoxin 1 (PRDX1) has been found to promote the polarization of M1 macrophages. This polarization activates signaling through CLEC4E, a member of the CLRs family, and the SYK system. These signaling pathways, in turn, activate the transcription factor NF-κB, thereby enhancing the production of pro-inflammatory cytokines.^257^

The regulation of NF-κB signaling in various physiological and pathological processes is highly complex, often mediated by direct and indirect interactions with other molecules. These interactions facilitate crosstalk with multiple signaling pathways, integrating diverse cellular signals. The dual role of NF-κB signaling—as both a protective and pathological factor—highlights its context-dependent nature. A comprehensive knowledge of these regulatory mechanisms is essential for utilizing NF-κB signaling in therapeutic applications. As research advances, our growing knowledge offers promising opportunities to manipulate this pathway for gene regulation, modulation of cellular behaviors, and the development of targeted therapeutic strategies.

### The cGAS–STING–IFN pathway

This year, the prestigious Albert Lasker Award for Basic Medical Research recognizes Zhijian Chen for his groundbreaking discovery of the mechanism by which the immune system detects DNA, specifically the identification of the enzyme cGAS. cGAS uniquely binds to DNA and catalyzes the synthesis of cyclic GMP–AMP (cGAMP), a second messenger that activates STING, leading to the induction of interferon (IFN) responses via the transcription factor IRF3. The elucidation of cGAS and its downstream signaling pathways has profound implications for advancing both basic and biomedical research, with potential applications in immunology, cancer therapy, and infectious disease.^258^ Genomic or mitochondrial dsDNA can originate from viral, bacterial, or other pathogenic infections, as well as from dying, damaged cells, or cellular stress. In the cytosol, cGAS dimerizes and binds to dsDNA, leading to its enzymatic activation and the subsequent synthesis of cGAMP. This cGAMP then interacts with STING dimers located at the ER, inducing STING’s oligomerization and release from the ER membrane. STING subsequently recruits the kinase TBK1, which phosphorylates STING and helps recruit IRF3.^259^ Phosphorylated IRF3 forms dimers and moves to the nucleus, where it promotes the expression of IFN-I genes (Fig. 8e). Upon extracellular secretion, IFN-I and cytokines bind to their respective receptors on the cell membrane, which in turn activates the JAK-STAT signaling pathway.^260^

STING not only activates the IFN-I response but also stimulates both canonical and noncanonical NF-κB signaling pathways, which in turn regulate other antimicrobial responses. Interestingly, noncanonical NF-κB signaling has been shown to negatively regulate STING’s effector mechanisms, providing a crucial regulatory feedback loop. Accumulating evidence from various PRRs and pathways shows that STING-IFN signaling plays a crucial role in modulating homeostasis and inflammation. NLRC3 engages HSV-60 dsDNA primarily via its LRR domain. This interaction enhances NLRC3’s ATPase activity, a crucial step for the liberation of STING and TBK1, subsequently orchestrating the downstream response.^261^ Furthermore, the scaffolding protein IQ motif containing GTPase activating protein 1 (IQGAP1) specifically interacts with NLRC3, disrupting the NLRC3-STING complex and thereby regulating the IFN-I pathway within the cytosolic environment.^262^

For the past few decades, an expanding body of literature has implicated the microbiota in the priming of the cGAS–STING–IFN-I pathway.^263–265^ While research has primarily concentrated on the role of TLRs in microbiota-induced IFN-I system priming, findings have been inconsistent, with conflicting roles proposed for TLR4^266^ and TLR7.^265^ Employing novel genetic mouse models, a recent in-depth analysis identified essential innate immune pathways in IFN-I priming, highlighting the gut microbiota’s pivotal role. The study demonstrated that gut microbiota primes the IFN-I system through constitutive activation of the cytosolic cGAS–STING pathway, which is crucial for innate resistance to both DNA viruses, such as HSV-1 and RNA viruses, such as vesicular stomatitis virus (VSV). This microbial-dependent activation of the cGAS–STING-IFN-I axis occurs indirectly, without requiring direct host-microbe interactions. Instead, it is mediated remotely through the delivery of bacterial DNA into distant host cells via membrane vesicles, highlighting a novel mechanism of host–microbiota communication in antiviral defense.^267^

### The Ca^2+^ signaling

Calcium (Ca^2+^), as a ubiquitous second messenger, plays a pivotal role in regulating a wide array of cellular biological processes, including proliferation, survival, apoptosis, and immune response.^268^ The ion’s intricate involvement in these processes is facilitated by its import and export through various Ca^2+^ channels, pumps, and transporters, enabling it to bind to numerous cellular proteins and control diverse cellular functions—a process known as Ca^2+^ signaling.^268^ Given its modulation of multiple fundamental biological processes, Ca^2+^ signaling is an attractive target for PRRs, including TLRs,^269^ RLRs,^270^ cytosolic DNA sensors,^271^ and NLRs.^272^ These PRRs leverage Ca²⁺ signaling to establish a favorable cellular environment for managing infections by exogenous pathogenic microorganisms or responding to endogenous danger signals. Conversely, emerging evidence indicates that Ca²⁺ signaling, mediated by ion channels in the plasma membrane and organelles such as the ER and mitochondria, can regulate the activation of these PRR subfamilies. This regulatory interplay enhances the activation of IRF3 and IRF7, initiating type I IFN-associated innate immune responses, while simultaneously amplifying NF-κB-driven inflammatory pathways, thereby fine-tuning the immune response (Fig. 8f).

To date, emerging studies have elucidated that PRRs are capable of initiating Ca^2+^ signaling, which subsequently regulates PRR-dependent cellular functions in a reciprocal manner. Furthermore, recent evidence has revealed that TLR7 augments the adenosine (cAMP)-protein kinase A (PKA) pathway, ultimately leading to increased expression of sarcoplasmic/endoplasmic reticulum Ca^2+^ ATPase (SERCA) and ryanodine receptor 2 (RYR2) in the sarcoplasmic reticulum, thereby disrupting Ca^2+^ signaling within cardiomyocytes.^273^ This mechanism underscores the cardioprotective role of TLR7 activation in septic cardiomyopathy-induced systolic dysfunction. In addition, TLR7-induced elevation of intracellular Ca^2+^, particularly when stimulated by imiquimod, is linked to inositol 1,4,5-trisphosphate (IP3)-dependent Ca^2+^ release from the ER via IP3 receptors (Fig. 8f). Moreover, extracellular Ca^2+^ functions as a DAMP, prompting the release of ER Ca^2+^ and activating the NLRP3 inflammasome through the calcium sensing receptor (CASR)/G protein-coupled receptor class C group 6 member A (GPRC6A)-phospholipase C beta (PLCB, also known as PLCβ) pathway.^274,275^

Ca²⁺ signaling serves as a key regulator of cytosolic DNA sensor function. Recent research has shown that STING, a critical regulator in this process, is a Ca^2+^-binding protein. During homodimer formation, STING monomers coordinate two shared Ca²⁺ ions—a feature essential for its proper structure and function.^276^ In its resting state, STING resides in the ER, where it interacts with the Ca^2+^-sensing transmembrane protein stromal interaction molecule 1 (STIM1) and the ER Ca^2+^ pump ATPase sarcoplasmic/endoplasmic reticulum Ca^2+^ transporting 2 (ATP2A2, also known as SERCA2). This interaction helps in maintaining STING in a non-activated state.^277^ Upon Ca²⁺ binding, STING undergoes structural reorganization and relocates to perinuclear compartments, where it oligomerizes to form a scaffold for TBK1 recruitment and subsequent downstream signaling activation. In addition, STIM1 not only regulates cytosolic Ca^2+^ concentration but also activates STING in response to changes in intracellular Ca^2+^ levels. This activation drives cellular proliferation, induces phenotypic switching in vascular smooth muscle cells (VSMCs), and accelerates atherosclerotic plaque progression.^278^ In a recent study using a C57BL/6 mouse model of experimental autoimmune encephalomyelitis, neuronal STING was identified as a master regulator of inflammation-driven neurodegeneration. The results establish a novel STING-dependent pathway linking interferon signaling with glutamate-mediated Ca²⁺ dynamics to execute ferroptosis. Thus, STING emerges as a master neuronal regulator at the crossroads of inflammation and Ca²⁺ imbalance, propelling neurodegeneration.^271^ Furthermore, a STING mutant unable to bind Ca^2+^ failed to trigger an IFN response under conditions of pathogenic stimuli insufficiency, highlighting the essential role of Ca^2+^ binding in STING’s function.

## Regulation of PRRs

While innate signaling plays a vital role in host defense, maintaining a balanced PRR signaling outcome is critical for effectively eliminating invading pathogens while avoiding excessive immunopathology. Achieving this delicate equilibrium between activation and suppression of PRR-mediated innate immune responses requires a variety of regulatory mechanisms. These mechanisms fine-tune PRR-triggered responses by modulating the transcription, post-transcriptional processing, translation, degradation, or subcellular localization of the receptors themselves, as well as through the involvement of intracellular regulators and amplifiers.

A wide range of regulatory molecules—including phosphatases, kinases, ubiquitin-related proteins, transcription factors (TFs), and epigenetic modifiers—target specific steps within the PRR signaling cascade to modulate the immune response. In addition, PRRs interact dynamically with other immune pathways, engaging in both cooperative and antagonistic cross-modulation. Extracellular factors, such as metabolic shifts, microenvironmental controls, and the gut microbiota, also play critical roles in shaping these responses (Table 2).

**Table 2:** Regulatory mechanisms of PRRs

Regulators of PRR signals		Examples
Transcriptional regulation of PRRs	Transcription factors	NLRC5.^280^ SUB1^281^ WRKY33^282^ etc.
	Enhancers and silencers	NF-κB^284^ etc.
	Epigenetic modifications	DNA methylation^286^ Histone acetylation^597^ etc.
Post-transcriptional regulation of PRRs	Alternative mRNA splicing and polyadenylation	Alternative polyadenylation^290^ etc.
	mRNA stability	mRNA decay^295^ N6-methyladenosine^298^ etc.
	Noncoding RNA regulation	microRNAs^302^ LncRNAs^310^ etc.
Translation regulation	Translation initiation	Canonical cap-dependent pathway: EIF2, mTOR, and EIF4EBPs^315,318,319^ Other mechanisms: non-AUG start codon utilization, translation re-initiation, leaky scanning, and internal ribosome entry sites^320^ etc.
	Gene-specific regulation	Association of RNA-binding proteins,^321^ such as ARE-binding proteins (TIA1)^322^ etc.
	Translation elongation and termination	EEF3^326^ EEF2^327^ EIF5A/EF-P^328^ etc.
Post-translational regulation	Post-translational modification	Phosphorylation^331^ Ubiquitination^598^ Glycosylation^599^ Acetylation^600^ Methylation^601^ Palmitoylation^331^ Neddylation^334^ Lactylation^336^ etc.
	PRRs degradation	Ubiquitin-proteasome system (ubiquitination)^340^ Autophagy-lysosome pathway^343^ etc.
	PRRs localization and trafficking	TLR4^345^ TLR7^348^ TLR9^349^ cGAS–STING^357^ etc.
Regulation of machinery assembly		Inflammasome assembly^364^ etc.
Recruitment of PRRs amplifiers		CCRL2^368^ miRNAs^371^ E3 ubiquitin ligases^372^ MHC class II molecules^373^ Ausome^370^ etc.
Recruitment of PRRs inhibitors		Phosphorylation^382–384^ Deubiquitination^377^ Acetylation^379^ DeSUMOylation^381^ Nucleases^385–387^ etc.
Cross-regulation of PRRs signaling pathways		Synergy^391–393,395^ Enhancement^205,396,397^ Suppression^409,411^ etc.
Metabolic regulation		Lipid metabolism^397^ Ketogenic diet metabolism.^426^ Purine metabolism^364,428^ etc.
Microenvironmental control		Inflammation^429^ Tissue hypoxia^435,436^ etc.
Gut-microbiota		*Schaedler flora*^440^ *Citrobacter rodentium*^442^ etc.

*CCRL2* C-C chemokine receptor type 2, *EIF2* eukaryotic initiation factor 2, *EIF4EBPs* eukaryotic initiation factor 4E-binding proteins, *EEF1A/EF-Tu* eukaryotic elongation factor 1A, *EEF2/EF-G* eukaryotic elongation factor 2, *EIF5A/EF-P* eukaryotic initiation factor 5A, *LncRNAs* long noncoding RNAs, *mTOR* mechanistic target of rapamycin, *NF-κB* nuclear factor Kappa-B, *NLRC5* NLR family C member 5 (NOD-like receptor family C member 5), *TLR4* Toll-like receptor 4, *WRKY33* WRKY DNA-binding protein 33

This intricate regulatory network ensures that innate immune signaling remains dynamic and adaptable, enabling the host to respond effectively to diverse challenges while minimizing the risk of collateral damage.^279^

### Transcriptional regulation of PRRs

Transcriptional regulation, a fundamental process in molecular biology, encompasses the complex mechanisms that control the transcription of genetic information from DNA to RNA. This transcriptional reprogramming is essential for host immunity, enabling precise responses to various PAMPs and DAMPs. The precise regulation of the immune transcriptome is critical to ensuring an adequate and context-specific host response.

**Fig. 9:**
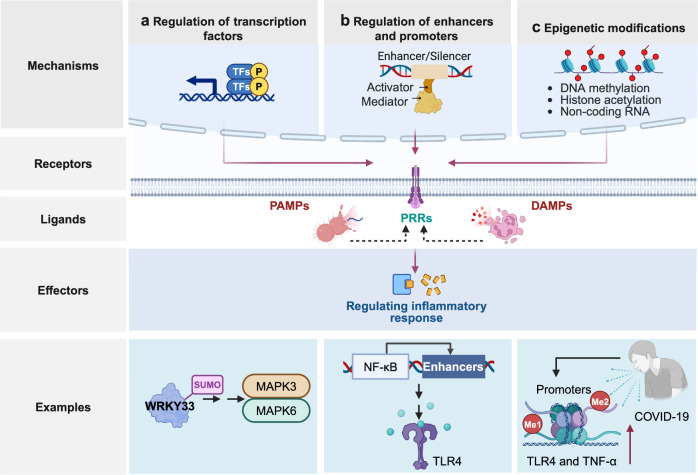
Transcriptional regulation in PRRs-mediated inflammatory response. **a** Activation and repression mediated by transcription factors. For example, the SUMOylation of the WRKY33 TF enhances its interaction with MAPK3 and MAPK6 of PRRs’ downstream signaling pathway. **b** Regulation of enhancers and promoters in PRRs. For example, NF-κB possesses the ability to activate poised enhancers and promoters among TLR4. **c** Epigenetic modifications in PRRs, including three pivotal epigenetic events—DNA methylation, histone acetylation, and noncoding RNA. For example, Patients with COVD-19 showed heightened methylation of the TLR4 and TNF-α. This figure is created by BioRender (https://app.biorender.com)

The transcription of genes encoding PRRs is governed by a multitude of interconnected regulatory mechanisms that influence transcriptional dynamics. These mechanisms include the activation or repression of gene expression by TFs, the impact of DNA-embedded enhancers and silencers, the regulation and post-translational modification of transcriptional regulators, and epigenetic modifications such as DNA methylation and histone modifications. In addition, noncoding RNAs (ncRNAs), modulation of RNA polymerase activity, alternative transcription initiation sites, and the involvement of the mediator complex play significant roles in shaping transcriptional outcomes (Fig. 9).

In the following discussion, we will explore several of these critical regulatory mechanisms and their implications for immune function.

#### Transcription factors

TFs are critical regulators of transcription, modulated at multiple levels to ensure precise control of gene expression. These regulatory mechanisms include PTMs, interactions with co-regulators and target DNA sequences, stability and turnover control, association with the Mediator complex, and regulation of RNA polymerase activity. In the context of the NLR inflammasome-associated family, evidence indicates that STAT3 is a key factor in regulating NLRC5 expression. NLRC5 expression at both mRNA and protein levels is upregulated by STAT3 activation in LPS-stimulated macrophages, identifying NLRC5 as a transcriptional target of STAT3. This finding highlights the elaborate interplay between TFs and inflammasome-associated genes in the process of immune response regulation.^280^ Beyond classical TFs, research has unveiled additional regulons and their associated TFs. For instance, a two-stage master regulator analysis, Virtual Inference of Protein-activity by Enriched Regulon (VIPER), was conducted on TLR2- and TLR4-responsive gene signatures in macrophages. Integrated analysis of coexpression networks from 371 carotid plaques identified SUB1/PC4 as a master transcriptional regulator of pro-atherogenic TLR signaling. Mechanistically, the TLR-responsive master regulator SUB1 activates IRF1 transcription, driving M1 macrophage polarization and exacerbating atherosclerotic plaque progression in vivo.^281^

PTMs are essential for regulating the activities of TFs. For example, the SUMOylation of the WRKY DNA-binding protein 33 (WRKY33) TF enhances its interaction with MAPK3 and MAPK6, subsequently boosting WRKY33’s phosphorylation through this specific pathway^282^ (Fig. 9a). Phosphorylation of WRKY33 via the calcium pathway augments its DNA-binding capability, while phosphorylation through the MAPK pathway elevates its transactivation activity.^283^ This regulation highlights the coordination of indole glucosinolates and camalexin biosynthesis by the signaling pathways of calcium-dependent protein kinase 5 (CPK5)/CPK6 and MPAK3/MAPK6.

#### Enhancers and silencers

Enhancers are regulatory DNA elements that can significantly increase gene transcription, even when located far from their associated promoters, whereas silencers exert the opposite effect by suppressing transcription. In quiescent macrophages, chromatin architecture is characterized by numerous poised enhancers—regions of DNA that are physically accessible and marked by histone modifications indicative of a transcriptionally ready but inactive state. Upon stimulation, nucleosome remodeling is induced at these latent enhancers and promoters, shaping the transcriptional response to TLR4 signaling in macrophages.

To investigate this process, researchers employed a combination of assays for transposase-accessible chromatin sequencing (ATAC-seq) and single-cell ATAC-seq to identify genomic regions undergoing remodeling. This analysis revealed that the transcription factor NF-κB binds to all high-confidence remodeling sites during the early response to the TLR4 ligand lipid A. Subsequent in vitro *and* in vivo studies confirmed the unique role of NF-κB among TLR4-activated transcription factors. NF-κB not only drives extensive nucleosome remodeling at inducible enhancers and promoters but also activates poised regulatory elements organized within open chromatin structures. These results establish NF-κB as a central mediator in orchestrating chromatin remodeling and transcriptional activation during the innate immune response^284^ (Fig. 9b).

#### Epigenetic modifications

Epigenetic modifications have the capacity to modulate the accessibility of DNA to TFs and RNA polymerase, all without altering the fundamental DNA sequence. Among these, three pivotal epigenetic events—DNA methylation, histone acetylation, and ncRNA—play essential roles in reshaping chromatin structure and dynamic repositioning of cis-regulatory elements, including promoters and enhancers, phenomena often observed in inflammatory conditions. In a COVID-19 case-control study stratified by disease severity, the author investigated epigenetic machinery gene expression and immune response gene promoter methylation patterns. The results indicated heightened methylation of the TLR4 and TNF-α promoters in patients versus controls (Fig. 9c). Conversely, patients with favorable outcomes (e.g., recovery) exhibited markedly reduced methylation of the TLR3 promoter.^285^ Furthermore, X inactive specific transcript (XIST), a long noncoding RNA (lncRNA) that is transcribed and spreads in cis to cover the entire inactive X chromosome, was found to regulate the transcription of TLR7, an X-linked gene. This regulation occurs via a mechanism involving X-inactivation, achieved through sustained deacetylation of histone H3 lysine 27 acetylation (H3K27ac) at the TLR7 promoter and modulation of RNA polymerase II elongation. This, in turn, inhibits the formation of CD11c^+^ atypical B cells.^286^

### Post-transcriptional regulation of PRRs

Although transcriptional regulation dominates innate immunity research, post-transcriptional mechanisms—including mRNA stability, splicing, and translational control—are equally vital in shaping macrophage and innate immune cell gene expression. The precise modulation of immune-related genes relies on a range of post-transcriptional regulatory mechanisms operating at multiple stages, including mRNA splicing, polyadenylation, stability, localization, and translation. These mechanisms are indispensable for fine-tuning the intensity and duration of immune responses, ensuring that they are appropriately scaled to the threat. Moreover, they are essential for the timely and efficient resolution of immune activity, preventing excessive or prolonged responses that could lead to tissue damage or chronic inflammation.

#### Alternative mRNA splicing and polyadenylation

A striking observation is that over 94% of human genes undergo alternative splicing or alternative polyadenylation.^287^ In human DCs, nearly one-fifth of the expressed genes exhibit alternative splicing in response to bacterial challenges. While many of these genes are involved in general cellular processes, some play direct roles in antimicrobial defense.^288^ Various types of alternative splicing can alter the protein-coding sequence, including mutually exclusive exon usage, exon skipping, intron retention, and alternative selection of 5’ or 3’ splice sites. In addition, alternative polyadenylation within an intron can produce mRNA encoding truncated proteins. The TLR signaling cascade exhibits exceptional susceptibility to post-transcriptional control, with >256 alternatively spliced transcripts generating functionally distinct variants of adaptor proteins, receptors, and downstream signaling components.^289^ Each TLR gene has many alternatively spliced variants, and TLR1 to TLR7 all have two to four predicted alternative polyadenylation sites.^290^ Utilizing mRNA sequencing and RNA-fluorescence in situ hybridization in the cecal ligation and puncture mouse model, differential splicing isoforms of TLR4 and other genes were confirmed in the lungs of mice with sepsis.^291^ These variant transcripts exert diverse effects on signal transduction.^292^ While most genes in the TLR pathway encode positive mediators of inflammatory signaling, several, including the gene for the MYD88 signaling adaptor, also generate alternative spliced mRNA isoforms that encode dominant-negative inhibitors of the response.^292^ The production of these negatively acting isoforms is induced by stimulation with the TLR4 agonist LPS, suggesting that this alternative pre-mRNA splicing constitutes a negative feedback loop that terminates TLR signaling and prevents chronic inflammation, as demonstrated in murine macrophages.^293^ These findings imply that the generation of truncated forms of TLR signaling pathway components creates a negative feedback mechanism that constrains excessive inflammation.

#### mRNA stability

Cellular mRNA levels are tightly regulated by the dynamic balance between mRNA production and degradation. Recent technological advancements, such as in vivo labeling of newly synthesized RNAs with modified uridines like 4-thiouridine (s(4)U) or bromodeoxyuridine (BrdU), along with isolation of chromatin-bound transcripts, have enabled precise quantification of steady-state and newly synthesized RNA levels These approaches have been particularly instrumental in studying cells stimulated with LPS or TNF, elucidating the coordinated transcriptional and post-transcriptional networks that orchestrate immune responses.^294^ This methodological progress has enabled the measurement of both transcription rates and RNA decay rates, shedding light on their respective roles in cellular responses. Analyses utilizing these techniques have revealed that the elevation of RNA levels in response to pro-inflammatory stimuli is primarily attributed to alterations in the transcription rate. Notably, the temporal control of rapid-response genes depends critically on RNA stability regulation.^295^ mRNA decay reduces mRNA abundance post-transcriptionally, balancing transcription and playing a crucial role in maintaining or altering mRNA expression levels.^295^ Exposure to LPS, TNF, and *Mycobacterium tuberculosis* can modulate the stability of many transcripts. TNF stimulation of fibroblasts causes the stabilization of 152 mRNAs and the destabilization of 58 others.^296^ The affected transcripts are enriched in inflammatory and immune signaling genes, as well as NF-κB targets.^297^ Collectively, these findings underscore the importance of regulating mRNA degradation in shaping innate immune responses.

Multiple mechanisms contribute to the regulation of mRNA stability, including adenylate/uridylate (AU)-rich elements (AREs), non-ARE-mediated pathways, nonsense-mediated mRNA decay, ncRNA-induced decay, and PTMs of RNAs, among others. As a pivotal regulatory mechanism governing gene expression, PTMs of RNAs have emerged in recent years. N6-methyladenosine (m6A) stands out as the most prevalent internal PTM in eukaryotic mRNAs, accounting for approximately 0.4% of all adenosine nucleotides in mammalian RNAs. The m6A methyltransferase complex, comprising METTL3, methyltransferase 14, N6-adenosine-methyltransferase non-catalytic subunit (METTL14), Wilms tumor 1 associated protein (WTAP), and vir-like m6A methyltransferase-associated (VIRMA, previously known as KIAA1429), plays a central role in this process. Studies utilizing Epstein–Barr virus (EBV)-infected Burkitt-like lymphoma cell (BJAB cell, also known as EBV genome-negative cell) models have demonstrated that TLR9 expression is suppressed through m6A modification. Mechanistically, Epstein–Barr nuclear antigen 1 (EBNA1) enhances the proteasomal degradation of METTL3, an m6A “writer,” leading to reduced TLR9 protein expression by destabilizing its mRNA via the lysine at position 48 (K48, also known as Lys48)-linked ubiquitin-proteasome pathway. Furthermore, YTH N6-methyladenosine RNA-binding protein F1 (YTHDF1) has been identified as an m6A “reader” for TLR9, promoting its expression by facilitating mRNA translation in an m6A-dependent manner.^298^

The influence of m6A modification goes beyond the regulation of individual gene expression, playing a crucial role in modulating PRR-associated signaling pathways. For example, the m6A methyltransferase subunit METTL14 in myeloid cells has been shown to amplify macrophage responses during acute bacterial infection in mice, leading to increased mortality due to sustained production of pro-inflammatory cytokines. Mechanistically, the depletion of METTL14 disrupts m6A methylation of the suppressor of cytokine signaling 1 (SOCS1) transcript, reducing its recognition by the m6A reader protein YTHDF1. This impairment diminishes SOCS1 expression, a key negative regulator of inflammation, resulting in the overactivation of the TLR4/NF-κB signaling pathway.^299^ These findings underscore the essential role of m6A modifications in controlling mRNA stability and modulating immune responses. Dysregulation of this pathway can exacerbate systemic inflammation, underscoring the importance of m6A-mediated control of mRNA dynamics in disorders characterized by excessive inflammatory responses.

#### Noncoding RNA regulation

**Fig. 10:**
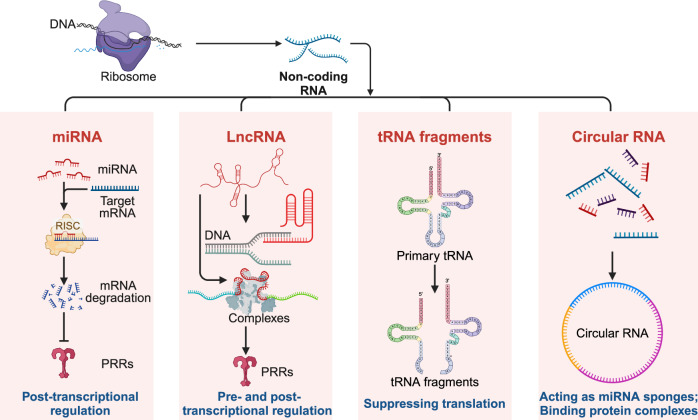
Noncoding RNA regulation in PRRs. A variety of noncoding RNA families, such as miRNAs, lncRNAs, tRNA fragments, and circRNAs, have been recognized as pivotal regulators in biological processes like transcription, splicing, and translation. These regulatory roles can significantly influence PRRs-mediated immune responses, adapted from Carpenter et al.^390^ with some modifications

NcRNAs play pivotal roles in RNA regulatory networks via interactions with RBPs and RNA-RNA interactions. Despite the fact that only 2% of the human genome encodes for proteins, over 85% is transcribed into ncRNAs, including microRNAs (miRNAs), lncRNAs, tRNA fragments, and circRNAs^294,300^ (Fig. 10).

MiRNAs, which are ssRNA molecules ranging from 21 to 23 nucleotides in length, undergo extensive processing before being incorporated into the RNA-induced silencing complex (RISC). Within RISC, miRNAs bind to the 3’ untranslated regions (UTRs) of target mRNAs through their “seed sequence” spanning nucleotides 2–8. This binding leads to the degradation or translational repression of the target mRNAs.^301^ Among the various regulatory elements within 3’ UTRs, miRNAs are critical modulators of mRNA stability and translation. Recognized for their ability to regulate antiviral immune responses, several miRNAs interact with diverse components and regulators of the RLR signaling pathway. For instance, microRNA-485 (miR-485), which is induced upon viral detection, targets the 3’ UTR of RIG-I. Ectopic expression of miR-485 reduces RIG-I levels, thereby inhibiting type I and III IFN production and facilitating viral infection.^302^ Furthermore, miR-204-3p, specifically expressed in macrophages, inhibits TLR4/c-JNK signaling and the secretion of pro-inflammatory cytokines. This process restricts fat accumulation, hepatic inflammation, and fibrogenic activation in hepatic stellate cells, ultimately alleviating steatohepatitis in mice fed a high-fat diet or a methionine- and choline-deficient diet.^303^ In addition, miR-182, induced by TGF-β signaling in tumor-associated macrophages (TAMs), directly represses TLR4 expression by dose-dependently inhibiting TLR4 3’ UTR activity. This results in suppression of NF-κB signaling and consequent M2-like polarization of tumor-associated macrophages in breast cancer mouse models, highlighting the potential of miR-182 targeting in reprogramming TAMs and limiting breast cancer progression.^304^ Together, these results establish a compelling foundation for developing RNA-based therapeutics targeting PPRs to treat infectious diseases, metabolic syndromes, and malignancies.

LncRNAs represent a class of noncoding transcripts >200 nucleotides in length, with thousands of loci annotated in the human genome. They frequently exhibit differential expression patterns during viral infections and immune stimulation.^305^ Recently, lncRNAs have emerged as versatile regulators of transcription, protein function, and RNA activity, often functioning as molecular decoys, guides, or scaffolds.^306–309^ Cytoplasmic lncRNAs play a crucial role in regulating antiviral immune signaling through their interactions with various proteins in the RLR pathway. For instance, CFAP58 divergent transcript (CFAP58-DT, also known as lncITPRIP-1), an infection-induced lncRNA, enhances IFN production and restricts hepatitis C virus replication by acting as a cofactor for MDA-5. It binds to the C-terminus of MDA-5 and facilitates its oligomerization around target RNAs.^310^ Another example is small nucleolar RNA host gene 16 (SNHG16), which can exacerbate pulmonary inflammation by sponging miRNAs and upregulating TLR4 expression through mRNA stabilization. Furthermore, intra-pulmonary delivery of SNHG16 upregulates TLR4/TRAF6 expression, leading to increased cell death processes and exacerbating symptoms in SLE patients with alveolar hemorrhage.^311^ However, lncRNAs are not universally antiviral; they can sometimes promote infection by inhibiting viral RNA sensing. In infected mice, cytosolic lnc-*Lsm3b* directly suppresses antiviral signaling by interacting with the RIG-I sensor and competing for viral RNA binding. Lnc-*Lsm3b* stabilizes the CARD-helicase interaction by binding to stem-loop structures, thereby preventing the release of RIG-I’s autoinhibition. Consistently, the loss of lnc-*Lsm3b* both in vitro and in vivo results in amplified antiviral signaling and reduced vesicular stomatitis viral load.^312^ Recent reports suggest that lncRNAs can also function as PPR ligands in immune disorders.^313^ For example, in SLE, a systemic autoimmune disease with a pronounced sex bias, the XIST lncRNA has been identified as a rich source of TLR7 ligands. XIST RNA stimulates IFN-α production by plasmacytoid dendritic cells (pDCs) in a TLR7-dependent manner, and its levels correlate positively with disease activity and the IFN signature. This highlights XIST RNA’s role as a female-specific danger signal underlying the sex bias observed in SLE.^313^

Collectively, research on the regulation of PRRs by ncRNAs is a rapidly advancing field with profound implications for understanding innate immunity and its role in various diseases. While significant progress has been made, challenges remain, including the need for more robust tools to validate the roles of ncRNAs in PRR regulation. In addition, a critical area of investigation involves elucidating how ncRNA-mediated regulation of PRRs varies across different tissues, cell types, and pathological conditions.

### Translation regulation

Following transcription, the process of translation starts. Numerous signaling events in innate immunity necessitate rapid alterations in gene expression that outpace the speed of de novo transcription or alternative pre-mRNA splicing/processing. In such instances, modulating the translation of already existing mRNAs enables swifter dynamic responses. This requires a finely tuned and precise regulation of the translation process to ensure efficacy.

#### Translation initiation

**Fig. 11:**
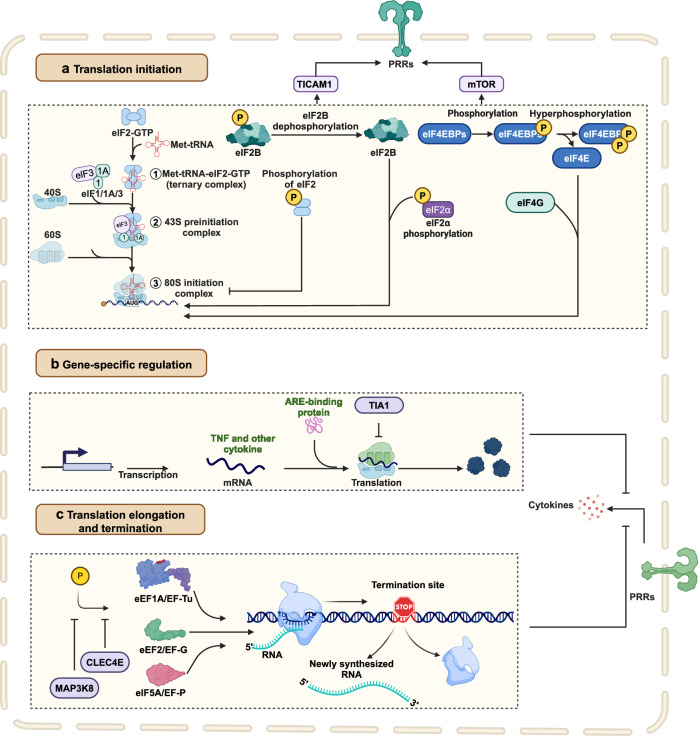
Translation regulations in PRRs. **a** Regulation of translation initiation in PRRs. For example, phosphorylation, hyperphosphorylation and dephosphorylation eukaryotic translation initiation factor induces regulation of translation, affecting most cellular PRR mRNAs’ expression, acting through the TICAM1or mTOR- mediated pathway. **b** Gene-specific regulation in PRRs. For example, TIA1, an ARE-binding protein, can repress the translation of TNF and other cytokine mRNAs following TLR stimulation by impeding their association with polyribosomes. **c** Regulation of translation elongation and termination in PRRs. For example, MAP3K8 and CLEC4E inhibit the translational machinery by dephosphorylating eukaryotic translation elongation factors, thereby suppressing PRRs-dependent cytokine production. This figure is created by BioRender (https://app.biorender.com)

Among the various translation initiation factors, eukaryotic translation initiation factor 2 (eIF2) stands out as the most extensively studied regulator in the context of innate immunity. eIF2 assembles into a ternary complex comprising the initiator methionyl-transfer RNA (Met-tRNA) and a guanosine triphosphate (GTP) molecule, which subsequently binds to the 40S ribosomal subunit. This binding is crucial for recognizing the start codon and recruiting the 60S ribosomal subunit. IF2α phosphorylation triggers widespread translational suppression, impacting both cellular and viral transcriptomes.^314^ This translation blockade provides a critical antiviral defense by inhibiting viral protein synthesis and limiting viral spread. Activation of TLR2, TLR3, or TLR4 in macrophages, bone marrow-derived macrophages, and fibroblasts triggers the dephosphorylation of eukaryotic translation initiation factor 2B (eIF2B) through the mediation of TICAM1^315–317^ (Fig. 11a). As a result, eIF2B’s guanine exchange activity is significantly increased, allowing eIF2 to recycle even when eIF2A is phosphorylated. This ensures sustained mRNA translation and enhances cell survival under prolonged stress.

The mechanistic target of rapamycin kinase (mTOR, also known as mammalian target of rapamycin) is a serine/threonine kinase that is responsive to diverse cellular stimuli, including PRR ligands. In macrophages, its activation is mediated through the MYD88-TICAM1-PI3K-AKT signaling cascade.^318^ Beyond regulating the transcription of immune-related genes, mTOR also plays a pivotal role in phosphorylating eukaryotic translation initiation factor 4E-binding proteins (eIF4EBPs, also known as 4EBPs). Upon mTOR activation, eIF4EBPs undergo hyperphosphorylation, leading to the release of eIF4E. This freed eIF4E can then bind to eIF4G and contribute to the translation process. For example, in mouse macrophages stimulated with LPS, mTOR enhances the translation of inflammatory genes mediated by TLR4, acting through the eIF4EBP1/ribosomal protein S6 kinase like 1 (RPS6KL1)/MAF bZIP transcription factor (MAF, also known as c-MAF) pathway.^319^ These findings highlight the importance of translational regulation by mTOR and eIF4EBPs in innate immunity.

While the canonical cap-dependent pathway is used for translating most mRNAs, a subset of cellular mRNAs employs alternative mechanisms for translation initiation. These mechanisms include the use of non-AUG start codons, translation re-initiation, leaky scanning, and internal ribosome entry sites (IRES), as comprehensively discussed in the publication “Post-Transcriptional Regulation of Gene Expression in Innate Immunity”.^320^ However, current research on the direct regulation of translation initiation in PRR-encoding mRNAs remains limited, underscoring the need for further investigation into this critical aspect of innate immune regulation.

#### Gene-specific regulation

Translation can be finely tuned for specific transcripts through the association of RBPs, lncRNAs, or small RNAs. These interactions can occur within the 5’UTR, coding sequence, or 3’UTR of target mRNAs and may depend on the primary sequence or specific RNA secondary structures. Among the most crucial TLR-dependent regulators of translation are ARE-binding proteins, owing to the presence of AREs in the 3’UTRs of many TLR4 mRNAs, which exhibit a strong affinity for RBPs.^321^ Besides their role in modulating mRNA stability (as discussed previously), ARE-binding proteins, which are implicated in regulating the translation of key ARE-containing mRNAs, respond to TLR activation. For example, TIA1 (also known as nucleolysin TIA-1 isoform p40), a cytotoxic granule-associated RNA-binding protein essential for translational control of TNF and other cytokines following TLR stimulation, represses translation of target mRNAs by preventing their association with polyribosomes^322^ (Fig. 11b). Furthermore, RBPs play a pivotal role in regulating PRR sensing and signaling by controlling the nuclear export of mRNAs. The DDX helicase domains embedded in RIG-I and MDA-5 regulate antiviral immunity by controlling nuclear export of host mRNAs essential for viral RNA detection pathways.^323^ For instance, DDX46 suppresses IFN-I responses during viral infection by sequestering MAVS, TRAF3, and TRAF6 transcripts in the nucleus, preventing their translation.^324^ The presence of m6A on these mRNAs promotes their nuclear export, exemplifying the modification’s role in subcellular RNA trafficking.^325^

In conclusion, the evidence identifies gene-specific translational regulation as a tunable node for modulating innate immune responses with therapeutic potential. Through simultaneous binding and competitive interactions, RBPs enable cells to integrate diverse signaling inputs, facilitating precise and targeted regulation of gene expression. This underscores the existence of a sophisticated post-transcriptional regulatory framework, wherein the combinatorial binding of RBPs to specific transcripts ultimately dictates their expression levels, ensuring context-specific immune responses.

#### Translation elongation and termination

While the initiation stage is commonly regarded as the primary site of translational regulation, control mechanisms also exist at the elongation and termination phases. In contrast to the intricate factor requirements for translation initiation, elongation proceeds with the aid of a relatively limited set of factors. These include the canonical eukaryotic translation elongation factor 1 alpha (eEF1A)/elongation factor thermo unstable (EF-Tu) and eEF2/EF-G, as well as the conserved eIF5A/EF-P, which is present in both eukaryotes and bacteria. The ATPase eEF3 appears to be specific to fungi and possibly certain other unicellular eukaryotes.^326^ Within this context, activation of TLRs in macrophages deficient in mitogen-activated protein kinase kinase kinase 8 (MAP3K8, also known as COT or Tpl-2) leads to reduced phosphorylation of eukaryotic elongation factor 2 kinase (eEF2K), suggesting a pivotal role for MAP3K8 in regulating translation elongation.^327^ In a mycobacterial granuloma model, persistent activation of TLRs and CLEC4E by mycobacterial components inhibited the general translational machinery through eIF4EBP1 dephosphorylation. Conversely, CLEC4E-induced robust nitric oxide release was mediated by enhanced translation of key enzymes via p38-dependent EIF5A hypusination, thereby inhibiting NLRP3-dependent IL-1β production (Fig. 11c). These findings emphasize the pivotal role of EIF5A in regulating the translation of specific transcripts essential for nitric oxide production and the resolution of inflammation in macrophages.^328^ However, despite these insights, the mechanisms governing elongation and termination, as well as their implications in physiological and pathological processes, remain largely unexplored.

### Post-translational regulation

#### Post-translational modification (PTM)

PTMs of PRRs and their downstream signaling molecules are critical for modulating their activity and function. These modifications involve the covalent attachment of functional groups such as phosphate, methyl, or acetyl groups. Classical PTMs include phosphorylation, ubiquitination, glycosylation (attachment of sugar molecules), acetylation, methylation, sulfation, glutamylation, and palmitoylation.

In addition to these traditional PTMs, a structurally similar but functionally distinct class of modifications involving ubiquitin-like proteins has emerged. These ubiquitin-like proteins, including SUMO, ISG15, ubiquitin D (UBD) (also known as FAT10), and NEDD8 (neuronal precursor cell-expressed developmentally downregulated 8), regulate the activity, interaction networks, or intracellular trafficking of target proteins. Unlike ubiquitination, which often promotes proteasomal degradation, these modifications influence diverse aspects of protein function, enhancing the complexity and precision of immune regulation.^329,330^ In recent years, several novel acyl modifications have been discovered, including succinylation, crotonylation, 2-hydroxyisobutyrylation, and lactylation. These PTMs have been shown to regulate PRR-dependent inflammatory signaling by targeting key components of the innate immune system, including sensors, adapters, enzymes, and TFs. These modifications can exert bidirectional modulation (positive and negative) effects on innate inflammatory signaling pathways, fine-tuning the immune response. In the subsequent section, we will explore some of the recently discovered regulatory mechanisms by which PTMs modulate eukaryotic innate immunity.

Phosphorylation stands as the most thoroughly examined type of PTM in innate immunity, regulated through the antagonistic interplay between kinases and phosphatases. The phosphorylation and dephosphorylation of proteins are pivotal in innate immune responses, as they control the activation and inactivation of numerous signaling molecules dependent on PRRs, such as TBK1, MAPKs, IKKα, IKKβ, IκBα, and IRF3. The phosphorylation of innate adaptor proteins MAVS, STING, and TICAM1 at a conserved pLxIS motif is indispensable for the recruitment of IRF3 and the subsequent production of IFN-Is, demonstrating the essential function of protein phosphorylation in activating innate immune sensing and signaling in response to viral infection. Furthermore, phosphorylation plays a balancing act in maintaining homeostasis across various branches of innate immunity. For instance, the PRR-associated kinase Botrytis-Induced Kinase 1 (BIK1) phosphorylates diacylglycerol kinase 5 (DGK5) at Ser-506, triggering a rapid burst of phosphatidic acid (a regulator of reactive oxygen species production) and activating plant immunity. Conversely, DGK5 is phosphorylated at Thr-446 by PRR-activated intracellular MAPK4, which subsequently inhibits DGK5 activity and phosphatidic acid production, leading to a reduction in plant immunity.^331^ Recently, a phospho-switch mechanism was reported to abolish pattern-triggered immunity while triggering intracellular NLR-mediated autoimmunity in plant immunity, albeit with an unclear mechanism. This mechanism enables and constrains immune activation, thereby maintaining cellular homeostasis.^332^

Other PTMs also play crucial roles in regulating PRR-triggered immune responses. S-palmitoylation, a reversible lipid modification of cysteine residues on proteins, is crucial for regulating biological processes associated with PRRs. Recent findings indicate that palmitoyl-protein thioesterase 1 (PPT1) influences TLR9 function by removing its S-palmitoylation in lysosomes, affecting TLR9 trafficking and autoimmunity in SLE.^333^ Biochemical assays and mass spectrometry have shown that TLR9 is S-palmitoylated at cysteine residues C258 and C265. The protein acyltransferase zinc finger DHHC-type palmitoyltransferase 3 (ZDHHC3, also known as DHHC3) catalyzes this modification within the Golgi apparatus, influencing TLR9’s endosomal trafficking. PPT1-mediated depalmitoylation enables TLR9 to dissociate from UNC-93 homolog B1, TLR signaling regulator (UNC93B1), boosting IFNα secretion by plasmacytoid dendritic cells (pDCs) and TNF by macrophages. Notably, administering the PPT1 inhibitor hexadecylsulfonylfluoride (HDSF) to both healthy volunteers and SLE patients reduces IFNα production ex vivo, underscoring its therapeutic potential for autoimmune diseases. S-palmitoylation of NOD1/2 has been identified as essential for their membrane recruitment and subsequent immune signaling. Specifically, ZDHHC5 has been identified as the palmitoyltransferase mediating this vital modification, and several disease-associated mutations in NOD2 have been linked to defective S-palmitoylation. Consequently, ZDHHC5-mediated S-palmitoylation of NOD1/2 is indispensable for their responsiveness to peptidoglycans and the mounting of an effective immune response.^119^

Protein neddylation, another biochemical process, involves the attachment of the ubiquitin-like molecule NEDD8 to a lysine residue within a substrate protein.^334^ Upon activation, NEDD8 is transferred to the NEDD8-conjugating E2 enzyme, ubiquitin conjugating enzyme E2 M (UBE2M). Mechanistic studies have revealed that UBE2M inhibits the degradation of RIG-I by preventing its binding to the stress-induced phosphoprotein 1 (STIP1) homology and U-box containing protein 1 (STUB1) E3 ligase, thereby activating antiviral IFN-I signaling. This, in turn, activates STAT1 to transcriptionally upregulate tripartite motif containing 21 (TRIM21), an E3 ligase that promotes UBE2M ubiquitylation and degradation, leading to the inhibition of antiviral immunity. Thus, disrupting this negative feedback loop of the IFN-I signal to maintain high levels of UBE2M enhances innate immunity against RNA viruses.^335^

In response to l-lactate, cGAS associates with the alanyl-tRNA synthetases alanyl-tRNA synthetase 1 (AARS1) and AARS2 (AARS1/2), which function as intracellular l-lactate sensors. This interaction mediates the N-terminal lactylation and inactivation of cGAS in both cells and mice. Mechanistically, through the establishment of an orthogonal genetic code expansion system for lactyl-lysine incorporation, it has been demonstrated that the N-terminal lysine residues 131 and 156 of cGAS can be directly lactylated by AARS1/2. Following lactylation, cGAS loses its ability to recognize dsDNA and inhibits liquid-liquid phase separation, resulting in a significant decrease in cGAMP and severely suppressing the activation of innate immunity mediated by the cGAS–STING pathway.^336^ Compared to the extensive research on conventional PTMs, the study of unconventional PTMs remains in its early stages, presenting significant challenges in uncovering their specific roles and interconnectivity within immune signaling pathways. Future research should focus on elucidating the precise molecular mechanisms underlying these unconventional PTMs and their functional crosstalk with conventional PTMs. Such investigations are essential for understanding the integrated regulatory networks governing immune signaling cascades and their implications for host defense and disease.^329^

#### PRRs degradation

In eukaryotic cells, protein degradation is primarily facilitated by two main pathways: the ubiquitin-proteasome system and the autophagy-lysosome pathway. These mechanisms are essential for regulating the responses and signaling pathways of PRRs through proteolytic cleavage.^337^ Ubiquitination marks certain PRRs, such as TLRs, proteasome-mediated degradation. For instance, the E3 ubiquitin ligase ring finger protein 216 (RNF216, also known as TRIAD3A) catalyzes ubiquitylation of TLR4 and TLR9 results in their subsequent degradation, thereby inhibiting their signaling pathways. This process is vital for maintaining immune homeostasis. Proteolytic cleavage is also required for the functional maturation of some PRRs. The N- and C-terminal domains of TLR7, after being cleaved in primary immune cells such as pDCs, are involved in ligand binding to the severe acute respiratory syndrome coronavirus 2 (SARS-CoV-2).^338,339^ This cleavage is essential for TLR7’s role in signaling.

Viruses have evolved strategies to exploit these degradation pathways to evade the immune response. For example, the influenza virus uses its non-structural protein 1 to interact with the E3 ligases TRIM25 and ring finger protein 135 (RNF135, also known as riplet), thereby blocking the K63-polyubiquitination of RIG-I and inhibiting IFN induction.^340^ Similarly, other viruses such as Sendai virus and vesicular stomatitis virus upregulate the lectin family protein Siglec-G, which promotes the interaction between the E3 ligase Cbl proto-oncogene (CBL, also known as c-Cbl) and RIG-I, leading to its proteasomal degradation.^341^ Furthermore, viruses can also target downstream molecules in the PRR signaling pathways for degradation. SARS-CoV, for instance, usurps the itchy E3 ubiquitin protein ligase (ITCH, also known as AIP4) to degrade MAVS, a key component in the innate immune response.^342^ Interestingly, the unique open reading frame 10 (ORF10) of SARS-CoV-2 targets MAVS for degradation through the autophagy-lysosome pathway, thereby suppressing the IFN-I signaling pathway.^343^ In summary, pathogens utilize diverse strategies to modulate innate immunity by targeting PRRs and their associated signaling pathways for degradation. These mechanisms not only illustrate the complexity of the immune system but also emphasize the sophisticated tactics pathogens employ to evade host defenses and establish infections.

#### PRRs localization and trafficking

The continual translocation of PRRs among diverse subcellular organelles and their recycling from intracellular compartments to membrane surfaces are finely tuned by an array of accessory molecules.^344^ It is vital for sustaining host defense against microbial invaders that TLR4 is trafficked from intracellular compartments, including the Golgi and endosomes, to the cell membrane. In this context, the small GTPase RAB10, member RAS oncogene family (RAB10), induced by LPS stimulation, facilitates LR4 reinsertion into the plasma membrane, thereby augmenting TLR4 signaling pathways.^345^

The RNA sensor TLR7 undertakes the challenging role of efficiently recognizing and responding to foreign genomic material while maintaining tolerance towards self-derived nucleic acids. This homeostatic equilibrium is maintained by TLR7’s selective retention in late endosomes, enabling efficient detection of phagocytosed pathogen-derived RNA while minimizing contact with endogenous nucleic acids.^346^ To achieve signaling competence within late endosomes, TLR7 relies on complex interactions involving transport mechanisms, membrane associations, and proteolytic processing within acidic endosomal compartments. Unique cysteine residues in TLR7 are critical for forming intramolecular disulfide bonds, which facilitate its proteolytic cleavage and enable effective RNA sensing.^347^ Olivia Majer and colleagues have elucidated that the late endosomal BLOC-1 (biogenesis of lysosomal organelles complex 1) related complex (BORC), in conjunction with the small guanosine triphosphatase (GTPase) ADP-ribosylation factor (ARF) like GTPase 8B (ARL8B), regulates intracellular TLR7 levels by modulating receptor turnover. UNC93B1-ARL8B binding is essential for TLR7 transport. Disrupted endosomal degradation leads to aberrant TLR7 signaling, providing a molecular basis for lupus-like autoimmunity in humans.^348^ Recently, Olivia Majer’ team also identified another novel regulatory mechanism that restricts TLR9 activation to endosomal compartments.^349^ LR9 signaling is regulated through endosome-specific dissociation from UNC93B1, a prerequisite for ligand engagement and downstream activation. Disrupting this process—via UNC93B1 mutations that prolong TLR9 binding or synthetic tethers blocking release—abolishes TLR9 responses.

The ER-associated adapter protein STING is primarily localized in the ER and acts as a central hub for various cytosolic DNA sensors. Upon binding cGAMP produced by cGAS, STING undergoes sequential trafficking from the ER to the Golgi apparatus, ultimately accumulating in perinuclear puncta.^350^ These structures facilitate the recruitment of TBK1, initiating the IFN-mediated antiviral response. After moving through the Golgi, vesicles containing STING are targeted to lysosomes, where they undergo progressive degradation.^351^ Extracellular cGAMP can be imported into adjacent cells through distinct transmembrane transporters to activate STING signaling, including solute carrier family 19 member 1 (SLC19A1),^352^ solute carrier family 46 member 2 (SLC46A2)^353^ and eucine rich repeat containing 8 (LRRC8, also known as volume regulated anion channel subunits.^354^ ATP binding cassette subfamily C member 1 (ABCC1 blood group) ABCC1), also known as the multidrug resistance protein 1 (MRP1), is an essential exporter of cGAMP, playing a pivotal role in modulating STING-dependent immunity.^355^ Recent studies have elucidated the structure of ABCC1, highlighting the molecular mechanisms of cGAMP export.^356^ The N-terminal transmembrane domain of ABCC1 promotes homodimer formation, while the ligand-bound structure reveals that cGAMP is specifically recognized by a positively charged pocket within the transporter, facilitating its export.^356^

Transporter-mediated export is not the only mechanism through which STING facilitates the release of extracellular CDNs. Plasmatic membrane-associated STING, an isoform lacking a transmembrane domain compared to its canonical counterpart, is anchored in the plasma membrane with its C-terminus exposed externally. This variant directly detects extracellular cGAMP, initiating the TBK1/IRF3/IFN signaling cascade.^357^ In addition, cGAMP synthesized by cGAS can be transmitted to adjacent cells via gap junctions, engaging plasma membrane-bound STING and triggering a similar response.^358^ Extracellular cGAMP not engaged in signaling can be degraded by the membrane-associated enzyme ectonucleotide pyrophosphatase/phosphodiesterase 1 (ENPP1). Overall, DNA stimulation prompts a coordinated spatiotemporal regulation of cGAS–STING trafficking and the assembly of signaling components, managed by precise regulatory mechanisms across various membrane compartments.

These findings underscore the critical importance of regulatory mechanisms, such as the controlled dissociation of TLRs from chaperones, the proper turnover of TLRs, and the organelle-trafficking of cGAS–STING, as checkpoints for the initiation and maintenance of PRRs signaling and immune responses.

### Regulation of machinery assembly

The regulation of machinery assembly acts as a critical mediator of the formation and stability of PRRs and signaling complexes, thereby promoting or inhibiting downstream signal transduction pathways. Microglial activation is characterized by the emergence and persistent expression of TLR4 inflammarafts—enlarged, cholesterol-enriched lipid rafts that serve as assembly platforms for TLR4 dimers and other inflammatory receptor complexes.^359^ Upon cellular activation, these TLR4 inflammarafts cluster and stabilize, facilitating the agonist-induced assembly of receptors into functional signaling complexes. For instance, in microglia stimulated by LPS, TLR4 dimer-containing lipid rafts persist for ~15 min before diminishing due to the internalization of the LPS-TLR4 complex.^360^ Recently, NAD(P)HX epimerase (NAXE, also known as apolipoprotein A-I binding protein [AIBP]), a key regulator of cellular cholesterol metabolism, has been identified as a potent modulator of TLR4 inflammarafts. By suppressing TLR4 dimer assembly and lipid raft clustering, NAXE mitigates neuropathic pain in CIPN mice, linking membrane microdomain organization to inflammatory sensitization.^361^ Furthermore, in an amyloid beta precursor protein (APP)/presenilin 1 (PSEN1, also known as PS1) mouse model of Alzheimer’s disease, Loss of NAXE exacerbates microglial TLR4 clustering into inflammarafts, which drives: (1) bioenergetic collapse, (2) oxidative damage, (3) amyloid plaque accrual, and (4) neurodegeneration.^362^

The inflammasome assembly constitutes a pivotal step in PRR signaling. This process heavily depends on death-fold domain-mediated interactions between the receptor and ASC, as well as the interaction between ASC and caspase-1. Manipulating these interactions presents an opportunity to regulate inflammasome signaling, particularly through the involvement of CARD-only proteins and PYD-only proteins (POPs), which appear in human and higher primate genomes but are undetectable in mouse or rat reference sequences.^363^ CARD-only proteins have been suggested to function as inflammasome-suppressive factors by sequestering caspase-1. However, they may also exert regulatory effects on other signaling pathways, given that CARD16 and CARD18 also interact with RIPK2. POPs, on the other hand, are relatively well-characterized. Specifically, three POPs (POP1, POP2, and POP3) interfere with inflammasome signaling at the level of PYD–PYD interactions.^364^ POP1, which binds to ASC and prevents ASC^PYD^-NLRP3^PYD^ interactions, has been hypothesized to block ASC^PYD^ nucleation at the receptor level.^365,366^ Bat ASC2 has emerged as a potent negative regulator of inflammasomes, demonstrating superior inhibitory effects compared to human ASC2.^367^ Bat ASC2 is highly expressed at both the mRNA and protein levels and demonstrates potent inhibitory activity against human and mouse inflammasomes, including NLRP3 and AIM2. Transgenic expression of bat ASC2 in mice significantly reduced the severity of peritonitis induced by gout-associated crystals and ASC particles. Furthermore, it effectively suppressed inflammasome activation triggered by SARS-CoV-2 immune complexes. These findings underscore the therapeutic potential of inflammasome negative regulators, such as bat ASC2, in mitigating inflammatory diseases.

### Recruitment of PRRs amplifiers

PRR signaling intensity is precisely controlled through a diverse array of amplifiers that facilitate the initiation and activation of inflammatory responses. These amplifiers play crucial roles in microbial defense, the persistence of chronic inflammation, and the progression of autoimmune diseases.

One such amplifier is C-C motif chemokine receptor-like 2 (CCRL2), an atypical nonsignaling receptor initially identified in LPS-stimulated macrophages. Recent studies have implicated CCRL2 in augmenting TLR4-mediated immune responses in various inflammatory contexts.^368^ Mechanistically, CCRL2 interacts with TLR4 on the cell surface, stabilizing membrane-bound TLR4 and enhancing downstream MYD88-NF-κB signaling in macrophages, thereby amplifying inflammatory responses. Additionally, innovative materials have been developed to potentiate adjuvant activity and stimulate PRR signaling pathways. For example, in a prophylactic mouse model, stearic acid-doped lipid nanoparticles (LNPs) co-loaded with ovalbumin-encoding mRNA and the TLR4 agonist monophosphoryl lipid A (MPLA) demonstrated spleen-specific mRNA expression following intravenous administration. This approach elicited heightened adjuvant activity and promoted type 1 helper T cell (Th1) immune responses through the activation of multiple TLRs.^369^ These findings underscore the potential of both endogenous amplifiers and engineered materials in modulating PRR signaling to enhance immune responses in therapeutic and prophylactic applications.

Furthermore, amplification effects can be induced by certain physical factors, leveraging advanced technological innovations. A recently reported immunomodulator, Ausome (Au + [exo]some), exemplifies this; it consists of a gold nanoparticle core functionalized with bacterial membrane constituents. The gold nanoparticle core mediates localized hyperthermia, while the bacterial membrane components coordinately activate both innate and adaptive immune pathways. Multiple pattern recognition receptors (PRRs) coordinately detect Au-somes, triggering robust pro-inflammatory cytokine production and activation of effector T cells and NK cells.^370^ Additional molecules also play positive roles in PRR-triggered innate immune responses. Extracellular miRNAs,^371^ E3 ubiquitin ligases,^372^ and MHC class II molecules^373^ have been established as essential modulators of TLR-dependent signaling cascades in host antiviral defense mechanisms. These findings elucidate the molecular basis of the efficient amplification of PRR-mediated immune activation and hold significant promise for a variety of therapeutic applications.

### Recruitment of PRRs inhibitors

Intracellular PRR signaling cascades are commonly subverted through enzymatic inhibitors that catalyze dephosphorylation or ubiquitination of key signaling intermediates, or physically block the oligomerization and activation of downstream complexes.^344^ LGP2, devoid of a signaling structural domain, is a well-characterized regulator of RIG-I and MDA-5 across multiple studies. Structural studies reveal that LGP2 suppresses RIG-I by either outcompeting it for RNA binding or physically obstructing CTD-mediated oligomerization required for MAVS activation.^69,374^ Moreover, the ubiquitination of RIG-I is inhibited by LGP2’s interaction with TRIM25, thereby negatively regulating RIG-I signaling.^375^ Conversely, LGP2 promotes MDA-5 signaling by modulating the interaction between MDA-5 and RNA, as well as filament assembly.^376^ Additional investigation is necessary to elucidate the precise regulatory role of LGP2 under specific physiological conditions.

Multiple mechanisms regulate negatively cGAS through PTM-dependent pathways. The deubiquitinating enzyme OTU deubiquitinase 3 (OTUD3) exhibits opposing effects: it enhances cGAS-mediated DNA sensing while inhibiting RLR-mediated RNA detection.^377^ Acetylation of lysine residues in the disordered N-terminal region of h-cGAS enhances its enzymatic activity,^378^ whereas acetylation at specific residues—Lys384, Lys394, and Lys414—suppresses cGAS activity.^379^ SUMOylation of cGAS at various sites exerts diverse regulatory impacts. SUMO-specific peptidase 2 (SENP2)-catalyzed deSUMOylation leads to cGAS degradation during the late stages of viral infection.^380^ Conversely, deSUMOylation mediated by SENP7 enhances cGAS activation by alleviating SUMO-induced inhibition of cytosolic DNA sensing.^381^ Furthermore, phosphorylation of h-cGAS by AKT, cyclin-dependent kinase 1 (CDK1), protein kinase, DNA-activated, catalytic subunit (PRKDC, also known as DNAPK), and aurora kinase A (AURKA) has been shown to inhibit its enzymatic activity.^382–384^

Generally, the digestion of ligands within the endosomal compartment serves as a negative regulator of TLR responses, thereby preventing the onset of autoimmune reactions and mitigating excessive activation of antiviral innate immunity. Several nucleases are known to modulate TLR activation, including ribonuclease T2 (RNASET2), deoxyribonuclease 1L3 (DNASE1L3, also known as deoxyribonuclease I like 3), deoxyribonuclease 2, lysosomal (DNASE2, also known as DNase II), phospholipase D family member 3 (PLD3, also known as phospholipase D3), and PLD4.^385–387^ Deficiencies in these nucleases can lead to the accumulation of nucleic acids in the cytoplasm upon endosomal rupture, subsequently activating the cytosolic nucleic acid-sensing pathway and inducing type I interferonopathies. These conditions can be ameliorated through the elimination of IFN-I or the inhibition of TLR trafficking.^388^ In addition, the physiological attributes of certain nucleases, which exhibit partial roles or functional redundancy, such as ribonuclease A family member 1, pancreatic (RNASE1, also known as RNase A) and deoxyribonuclease 1 (DNASE1, also known as DNase I), necessitate further investigation to elucidate their specific contributions.^389^

### Cross-regulation of PRRs signaling pathways

Due to the diversity of PAMPs and DAMPs detected by PRRs and their activation of independent yet interconnected signaling pathways, effective immune responses require coordinated PRR interactions.^390^ Xuetao Cao has detailed the cross-regulatory interactions among PRRs, organizing them into five strategies that can be either synergistic or antagonistic.^279^

**Fig. 12:**
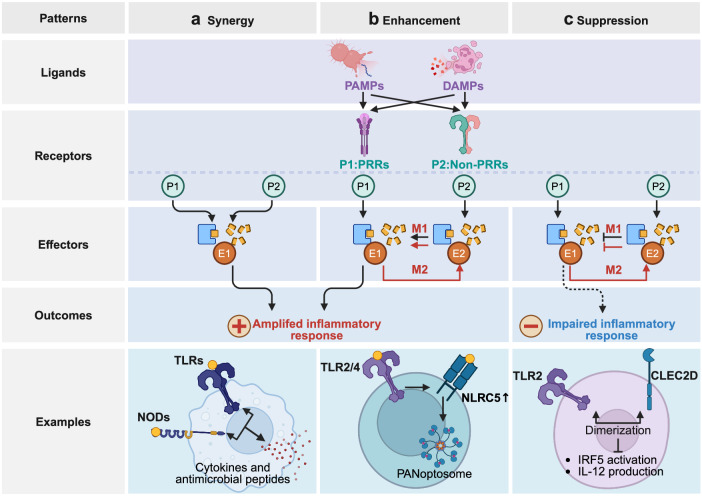
Different mechanistic models for cross-regulation of PRR signaling pathways. In principle, the cooperation of distinct PRR and non-PRRs signals cross-regulates to generate effector responses through three types of mechanisms: synergy, enhancement, and suppression. **a** Synergy. Different receptor-mediated signals in a responding cell efficiently generate an amplified inflammatory response. This phenomenon can occur between PRRs of the same class, different PRR types, distinct downstream signaling pathways of PRRs, and even between PRRs and non-PRRs. For example, NODs and TLRs synergistically induce the production of cytokines and antimicrobial peptides. **b** Enhancement. One receptor-mediated effector response (E1) is augmented by another receptor-induced effector mechanism (E2). This “enhancement” effect can be implemented through a direct mechanism (M1) or a positive feedback mechanism (M2). For example, TLR2 as well as TLR4 can upregulate NLRC5 expression and promote the assembly of the PANoptosome. **c** Suppression. A one receptor-mediated effector response (E1) is inhibited by a distinct receptor-induced effector mechanism (E2) to introduce amplified inflammatory response. This “suppressive” effect can be achieved either through a direct mechanism (M1) or via a negative feedback loop (M2). For example, C-type lectin domain family 2 member D (CLEC2D) has been shown to form dimers with TLR2, leading to inhibiting the activation of the transcription factor IRF5 and subsequent IL-12 production. This figure is created by BioRender (https://app.biorender.com)

To simplify this framework, we propose a streamlined classification into three subgroups: synergy, enhancement, and suppression (Fig. 12). Building on this model, our aim is to expand upon and refine the existing literature by exploring the molecular mechanisms underlying these interactions. This approach seeks to provide a more comprehensive understanding of how PRRs collaborate and regulate one another to fine-tune innate immune responses.

(1) Synergy (Fig. 12a). PRR-mediated signaling pathways often exhibit synergy, leading to an enhanced effector response. This phenomenon can occur between PRRs of the same class,^391^ different PRR types,^392^ distinct downstream signaling pathways of PRRs,^393^ and even between PRRs and other receptors.^394^ A basic approach for a restricted number of TLR classes to handle the vast array of PAMPs is to form combinatorial heterodimers.^395^ Both in vitro and in vivo experimental data indicate that NODs and TLRs synergistically induce the synthesis of cytokines and antimicrobial peptides. The molecular mechanisms underlying this synergy include direct interactions between downstream signaling pathways of NODs and TLRs, mutual transcriptional regulation of unique signaling components, and post-transcriptional interactions.^392^ On one hand, synergistic interactions between PRRs can contribute to excessive pro-inflammatory cytokine production, potentially necessitating the joint blockade of multiple PRRs to achieve therapeutic benefit in conditions characterized by hyperinflammation. On the other hand, leveraging synergistic combinations of low doses of PRR agonists administered prophylactically could enhance innate resistance against bacterial pathogens, offering a strategy to bolster immune defenses prior to infection.

(2) Enhancement (Fig. 12b). Beyond the synergistic interactions that intensify PRR-mediated responses, PRR-induced signals frequently augment effector responses through cross-regulation of their respective downstream signaling pathways. This “enhancement” is particularly crucial for the cooperation between PRRs and inflammasome signaling pathways. The concept of “priming,” which involves PRRs or cytokine receptors, is well-established in NLRP3 activation. Initially, priming was believed to solely upregulate NLRP3 expression transcriptionally; however, recent findings reveal that it also licenses inflammasome signaling by facilitating the deubiquitylation of NLRP3 via the Lys63-specific deubiquitinase BRCC3.^210^ TLR stimulation activates NF-κB, inducing IL-1β mRNA transcription and pro-IL-1β synthesis. Meanwhile, NLRP-mediated inflammasome assembly activates caspase-1, which cleaves pro-IL-1β to generate mature, bioactive IL-1β.^396^ Furthermore, it has been documented that TLR2 and TLR4 associate with their respective ligands, including PAMP/DAMP (such as heme) and DAMP/cytokine combinations, to upregulate NLRC5 expression and promote the assembly of the PANoptosome. This, in turn, triggers inflammatory cell death—PANoptosis—along with the release of cytokines and DAMPs.^205^

PRRs and various other systems also exhibit inter-regulatory capabilities, enhancing inflammatory responses through feedback mechanisms. For instance, in postpartum Gram-negative bacterial infections associated with Bovine endometritis, crosstalk between the TLR4 and IL-6R/STAT3 signaling pathways has been documented. This interaction signifies a positive feedback loop where IL-6R augments the release of IL-6 and IL-8 induced by TLR4 during the innate immune response.^397^ Similarly, a positive feedback model has been proposed to elucidate morphine-induced persistent sensitization. This model involves an initial surge of IL-1β triggered by morphine, followed by an exacerbated release of DAMPs, which in turn increases the activation of TLR4 and the purinergic receptor P2X 7 (P2RX7).^398^ Furthermore, opioid receptors can stimulate the production of high mobility group box 1 (HMGB1),^399^ an endogenous TLR4 agonist, facilitating intercellular communication (neuron-to-glia or glia-to-neuron).^398^ Additional signaling pathways, such as the complement system^400^ and tenascin-C signaling,^401^ also play crucial roles. While these signaling pathways independently serve as rapid innate defense mechanisms essential for initiating pro-inflammatory responses, their components can interact synergistically in positive feedback loops. This interaction reinforces the body’s first line of defense against pathogens and other injuries, enhancing overall immune resilience.

(3) Suppression (Fig. 12c). The host immune system has developed multiple mechanisms to prevent the overactivation of innate and adaptive immune responses resulting from synergistic crosstalk among different PRR signals, thereby mitigating excessive inflammation. LPS tolerance and cross-tolerance are essential for immune homeostasis, and their malfunction frequently leads to inflammatory diseases. While the mechanistic basis of LPS tolerance has been extensively studied and will not be elaborated here, it is important to note that the dimerization of certain PRRs, such as CLRs and TLRs, can influence ligand binding affinity and modulate immune responses. For example, C-type lectin domain family 2 member D (CLEC2D) has been shown to form both homodimers and heterodimers with TLR2. This interaction results in β-glucan-triggered ubiquitination and the subsequent breakdown of the adaptor protein MYD88, which in turn inhibits the activation of the transcription factor IRF5 and reduces IL-12 production. While this regulatory mechanism serves to dampen excessive inflammation, it also negatively impacts the host’s antifungal immunity, highlighting the delicate balance between immune regulation and effective pathogen defense.^402^ Dysregulated cGAS–STING pathway activation can result in autoimmune disorders or exacerbate the severity of abdominal sepsis.^403–408^ The homeostatic regulation of cGAS–STING involves toll-interacting protein (TOLLIP), an endogenous negative regulator of TLR signaling that functions as a stabilizer during the resting phase. The absence of TOLLIP leads to reduced STING levels in non-hematopoietic cells and tissues.^409^ In immune cells, TOLLIP deficiency destabilizes STING, preventing the production of cGAS–STING-dependent IFN-Is in response to cytosolic dsDNA.^410^ In addition, AIM2 activation serves as another endogenous negative regulator of cGAS–STING signaling and IFN-I production, acting through GSDMD to deplete intracellular potassium (K^+^) via membrane pore formation, inducing pyroptosis.^411^ These findings indicate that targeting tolerizing mechanisms, rather than directly suppressing inflammatory pathways, could provide promising therapeutic strategies for controlling uncontrollable infections.

A multitude of genes and processes are implicated in the feedback regulation of PRR-induced innate immune responses. These regulatory mechanisms are typically activated by ligands that primarily stimulate PRRs, functioning as intrinsic negative feedback systems to uphold homeostasis and avert excessive or perpetual activation of pro-inflammatory pathways triggered by PRRs.^412^ Through proteomic screening, the intramembrane protease rhomboid domain containing 1 (RHBDD1, also known as rhomboid-like 4 [RHBDL4]) has been pinpointed as a negative modulator of TLR4 signaling.^413^ Mechanistically, RHBDD1 promotes the degradation of transmembrane p24 trafficking protein 7 (TMED7), a critical trafficking factor for TLR4) thereby impairing the transport of TLR4 to the cell surface. TLR4 activation transcriptionally upregulates RHBDD1, establishing a negative feedback loop that limits TLR4 trafficking to the plasma membrane. This regulatory mechanism, which fine-tunes secretory cargo, mitigates overactivation of TLR4-dependent signaling, as demonstrated in a Mycobacterium tuberculosis infection model in macrophages, and alleviates septic shock in a murine model. Similarly, TRIM29, a ubiquitin E3 ligase, functions as an inducible negative regulator of the cGAS–STING-mediated innate immune response. Upon stimulation by DNA viruses or cytosolic DNA, TRIM29 expression is strongly induced in macrophages and DCs. TRIM29 subsequently catalyzes K48-linked ubiquitination of STING, targeting it for proteasomal degradation following activation. These mechanisms highlight critical pathways by which the immune system employs negative feedback to maintain balance and prevent excessive inflammatory responses.^414^ Cytokine storms arise during various systemic acute infections, including sepsis and the ongoing COVID-19 pandemic, causing severe inflammatory conditions. Endogenous negative regulators of TLR-signaling-mediated inflammatory pathways, which contribute to cytokine storms and sepsis, have been identified and extensively reviewed elsewhere. These regulators play crucial roles in terminating the immune response and maintaining immune homeostasis.^412^ Further research is imperative to elucidate how different PRRs impact distinct signaling molecules and to comprehend the physiological and pathological implications of their cellular and signaling selectivity. These studies will deepen our comprehension of innate immunity’s complex regulatory networks and could facilitate innovative therapeutic approaches for immune-related disorders.”

### Metabolic regulation

In recent years, the reciprocal interplay between PRR signaling and metabolic pathways has garnered significant interest.^415,416^ PRR activation in innate immune cells induces significant remodeling of lipid species and their spatial organization within cellular compartments. Biologically active lipid-derived mediators, in turn, play a pivotal role in both initiating and resolving inflammatory responses. Earlier research predominantly focused on exogenous saturated fatty acids, which have been implicated in directly activating TLR6 or remodeling plasma membrane lipid raft architecture.^417^ Conversely, fatty acid synthase (FASN) produces endogenous de novo fatty acids that serve as vital regulators of signal transduction, modulating TLR signaling through mechanisms distinct from established pathways.^418,419^ Research in murine sepsis models reveals that MYD88 palmitoylation dynamics depend on both endogenous fatty acid biosynthesis and CD36-dependent exogenous fatty acid incorporation. In vitro studies demonstrate that TLR4 responsiveness is attenuated through FASN inhibition, cysteine residue mutagenesis, or palmitoyl acyltransferase knockdown, revealing a previously unrecognized regulatory axis in inflammatory signaling.^229^ This finding underscores the intricate connection between lipid metabolism and innate immune signaling.^406,420,421^

The ketogenic diet has increasingly gained attention due to the putative functions of ketone bodies as metabolic mediators in the benefits associated with calorie restriction.^422–424^ Under conditions such as starvation, caloric restriction, intense exercise, or a ketogenic diet low in carbohydrates, elevated levels of ketone bodies—including β-hydroxybutyrate—suppress NLRP3 inflammasome activation. This inhibition subsequently reduces the production of pro-inflammatory cytokines IL-1β and IL-18 in response to stimuli like urate crystals, ATP, and lipotoxic fatty acids. This finding offers novel insights into the mechanisms underlying the anti-inflammatory benefits of caloric restriction.^425^ β-hydroxybutyrate mitigates osteolysis induced by CoCrMo alloy particles by modulating the NLRP3 inflammasome and osteoclast differentiation.^426^ This finding further supports the therapeutic potential of ketone bodies in inflammatory and degenerative diseases, highlighting the multifaceted benefits of the ketogenic diet.

Uric acid is a heterocyclic compound composed of carbon, nitrogen, oxygen, and hydrogen atoms. It represents the end product of purine metabolism, derived from purines found in various foods and those generated endogenously during normal cellular processes.^427^ When uric acid crystals are recognized as danger signals by immune cells, they can induce the assembly of the NLRP3 inflammasome, which mediates the cleavage and activation of pro-IL-1β and pro-IL-18 into their active cytokine forms. This process promotes inflammatory responses, highlighting the role of uric acid crystals as potent triggers of innate immune activation.^428^

### Microenvironmental control

The epigenomic and transcriptional programs, as well as the inflammatory signaling responses mediated by PRRs in both immune and non-immune cells, are tightly and dynamically regulated by the cellular microenvironment. Microenvironmental cues can significantly alter the responsiveness of macrophages to subsequent stimuli. Prior exposure to IL-4 induces epigenetic reprogramming in macrophages, leading to a substantial expansion of the RELA cistrome upon TLR activation. This reprogramming results in the activation of a unique hyperinflammatory gene expression program in both murine and human macrophages.

In the context of allergic airway inflammation, this phenomenon, termed “extended synergy,” is observed in alveolar macrophages. This mechanism enhances the macrophages’ responsiveness to TLR activation, driving exacerbated inflammatory responses and contributing to the pathogenesis of lung disorders. These findings highlight the interplay between prior cytokine exposure, epigenetic reprogramming, and PRR signaling in shaping macrophage function and disease outcomes.^429^

Tissue hypoxia is a frequent characteristic of infected and inflamed tissues, having a significant impact on the development of immune and inflammatory responses through microenvironmental regulation.^430^ A key player in this context is hypoxia inducible factor 1 subunit alpha (HIF1A, also known as HIF-1α), which plays a pivotal role in promoting angiogenesis,^431^ stimulating pro-inflammatory cytokine production,^432^ regulating myeloid cell maturation,^433^ enhancing ATP production,^434^ activating the NLRP3 inflammasome,^435^ and transcriptionally modulating TLR expression.^436^ The mechanisms underlying these effects have been most extensively studied in the case of TLR4. Under normoxic conditions, LPS-activated human monocytic cells and mature macrophages upregulate HIF1A mRNA transcription via an NF-κB-MAPK-dependent pathway. In hypoxic conditions, the additional post-translational stabilization of HIF1A, resulting from hydroxylase inhibition, leads to increased DNA-binding activity.^437^ Consequently, the combined activation of TLR by LPS and hypoxia synergistically enhances HIF-1 activity, thereby upregulating the expression of downstream innate immunity-related target genes. In conclusion, the modulation of microenvironmental factors such as hypoxia and HIF1A activity represents a potential therapeutic approach for managing inflammatory diseases. These findings underscore the importance of deciphering the intricate crosstalk between hypoxia, HIF1A, and innate immune signaling to develop targeted interventions.

### Gut-microbiota

The domain of microbiota research has seen swift development, shifting from concentrating on counting microorganisms like bacteria, viruses, and fungi at different human body locations to exploring how microbiota affects health and disease. The recognition of commensal bacteria by PRRs plays a role in maintaining host-microbe interactions and physiological homeostasis. Given their widespread expression across diverse cell types, PRRs represent a promising and innovative target for modulating host-microbe signaling. This regulation has the potential to mediate gut-organ crosstalk, offering new insights into microbiota-driven systemic effects.^438^ To investigate the interaction between gut bacteria and the pancreas in the context of PRR signaling, researchers conducted studies using non-obese diabetic (NOD) mice. They observed that specific-pathogen-free NOD mice lacking the MYD88 gene (MYD88^−/−^) did not develop type 1 diabetes mellitus, in contrast to their MYD88^+/−^ counterparts.^439^ Conversely, germ-free NOD-MYD88^−/−^ mice developed diabetes, but this was attenuated upon colonization with a defined microbial population, known as the altered *Schaedler flora*.^440^ These findings indicate that MYD88 signaling exerts its modulatory effects through both microbial-dependent and microbial-independent pathways.

Microbes are essential in the development and maturation of the immune system, providing protection to the host against bacterial and fungal pathogens. This protection is mediated through mechanisms such as competition for nutrients and attachment sites, as well as the production of antimicrobial compounds. IFN-Is are critical for antiviral immunity and are primarily induced through three families of PRRs: plasma and endosomal membrane-localized TLRs, intracellular RLRs, and cyclic cGAS.

Recent studies have highlighted microbiota-driven IFN-I priming as a result of TLR4 activation by bacterial glycolipids. However, another study demonstrated that the ablation of common adaptors for TLR signaling did not reduce basal IFN-I levels, suggesting the involvement of additional, TLR-independent pathways in maintaining baseline IFN-I production. These findings underscore the complex interplay between microbiota and innate immune signaling, advancing our understanding of host-microbe interactions in immune homeostasis.^264^ Furthermore, utilizing novel genetic mouse models, it was demonstrated that the priming of the IFN-I system by gut microbiota involves the tonic activation of the cytosolic cGAS–STING pathway. This activation is vital for innate resistance against DNA and RNA viral infections. This microbiota-induced activation of the cGAS–STING-IFN-I axis does not necessitate direct host-bacteria interactions. Instead, it occurs remotely through the delivery of bacterial DNA into distant host cells via membrane vesicles.^267^ These findings emphasize the significance of the microbiota in maintaining the immune system’s constant readiness against viruses and highlight the potential risks associated with the indiscriminate use of antibiotics during viral infections.

Dysbiosis has been documented in Alzheimer’s disease patients, hinting at a promising research direction to unravel the mechanisms and interplay between the brain and the host–microbiota. Recent findings suggest that two out of the four recognized families of PRRs, NLRs and TLRs, contributing to the pathogenesis of neurodevelopmental and neurodegenerative disorders through the gut-brain axis.^441^ Take the enteric mouse pathogen *Citrobacter rodentium* as an example; it induces an IL-17 reaction via mechanisms that rely on *NOD1* and *NOD2*.^442^ Mice lacking NOD1 and NOD2 exhibit heightened susceptibility to Listeria infection upon initial exposure to LPS or E. coli. Conversely, fecal microbiota transplantation therapy has shown remarkable efficacy in restoring the gut microbial community. This restoration subsequently inhibits the TLR4/MYD88/NF-κB signaling pathway and reduces downstream pro-inflammatory mediators in both the substantia nigra and the colon. Consequently, fecal microbiota transplantation alleviates gastrointestinal dysfunction and motor impairments in Parkinson’s disease mice.^443^ These studies indicate that gut microbiota restoration, through PRR-dependent pathways, may benefit the recipient microbiota and improve patient outcomes without complications.

## PRRs functions

### PRRs-mediated pathogen recognition

The innate immune system constitutes the first-line defense against pathogens, guarding against a wide array of pathogens, encompassing viruses, bacteria, fungi, and parasites. It acts to restrict viral invasion, translation, replication, and assembly, aids in the identification and elimination of infected cells, and orchestrates the rapid development of adaptive immunity. Various PRRs, located on the cell surface, within endosomes, and in the cytosol, respond to PAMPs. This response triggers inflammatory reactions and programmed cell death, effectively limiting pathogen infection and facilitating clearance. Nonetheless, an overly exuberant immune activation can precipitate systemic inflammation, cytokine storms, widespread cell death, tissue damage, organ failure, and ultimately, mortality.^444–446^ While the intricate roles of PRRs in pathogen recognition have been extensively reviewed, this section will provide a concise overview of key examples of PRR-mediated microbial recognition and their roles in infection processes.

CLRs play a crucial role in recognizing carbohydrate structures on various microorganisms, including viruses, bacteria, and fungi. The identification of CLEC7A as a β-glucan receptor that signals through SYK to alter the transcriptome of myeloid cells upon fungal detection has solidified CLRs’ status as bona fide PRRs.^447,448^ Dectin-1 and Dectin-2, which are CLRs coupled with ITAMs, are responsible for detecting β-glucans derived from fungi. DCs activated by either Dectin-1 or Dectin-2 can instruct T cells to elicit protective immunity against *Candida albicans*.^449^ In essence, the interaction between CLRs and PAMPs triggers intracellular signaling cascades that facilitate pathogen uptake and destruction, the production of inflammatory mediators, and the elicitation of various protective cellular responses, such as the respiratory burst and neutrophil extracellular trap formation.^80,450^ Furthermore, CLRs contribute to antigen (cross-)presentation and influence the induction and maturation of adaptive immune responses, encompassing Th1, IL-17-producing T helper (Th17), and cytotoxic T lymphocyte responses.^451–453^ However, the functions of CLRs can also have detrimental effects. For instance, they can modulate the expression of other key PRRs, impair antigen presentation, directly aid viral infection of myeloid cells, and promote pathological inflammation, including type 2 helper T cells (Th2) responses in allergic contexts.^454^ In addition, the impact of CLR-mediated pathogen recognition can be indirect, as it may involve the induction of immunomodulatory cytokines and other mediators. It is crucial to acknowledge that the outcome of an infection is influenced by the recognition of pathogens through multiple PRRs, whose intracellular signaling pathways integrate in complex and incompletely understood ways.

Recently, additional PRRs, such as cGAS, have emerged as key players in fungal recognition and the regulation of host immune responses. Research has demonstrated that the cGAS–STING pathway can detect *C. albicans* DNA associated with biofilms, which is packaged within extracellular vesicles. This detection was evidenced by the induction of IFN-stimulated genes, the production of IFN-β, and the phosphorylation of IRF3 and TBK1. This pathogen recognition mechanism is independent of the Dectin-1/CARD9 pathway and does not require TLR9. These findings provide new mechanistic understanding of cGAS in the early innate immune response triggered by this clinically significant fungal pathogen.^455^

The NLR family consists of cytoplasmic sensors of pathogens, characterized by a central nucleotide-binding domain and C-terminal LRRs.^456^ Most NLRs feature N-terminal protein-binding motifs, including CARDs, a PYD, and a baculovirus inhibitor of apoptosis protein repeat domain. NLRP3 plays a pivotal role in survival during pathogenic influenza A virus infection in murine models.^457^ Furthermore, NLRP3, alongside NLRP1, restricts the proliferation of Toxoplasma gondii and triggers IL-18 signaling to enhance host resistance against acute toxoplasmosis.^458^ Another key member, NLRC4, is essential for the innate immune response in defending against bacterial infections.^456^ Salmonella enterica serovar Typhimurium, a Gram-negative pathogen, utilizes two distinct type III secretion systems (T3SSs), known as Salmonella pathogenicity islands (SPIs) -1 and -2, to deliver virulence factors into host cells. SPI-1 T3SS enables Salmonella invasion, while SPI-2 T3SS supports its intracellular survival. In mice, cytosolic immune sensors NAIP1, NAIP2, and NAIP5/6 recognize the SPI-1 T3SS needle, inner rod, and flagellin proteins, respectively. This recognition triggers the assembly of the NAIP/NLRC4 inflammasome, leading to caspase-1 activation, secretion of IL-1 family cytokines, and pyroptosis of infected cells.^459^ NLRC4-deficient mice are more susceptible to *S. Typhimurium* infection, exhibiting higher bacterial loads in the cecum, liver, and spleen compared to wild-type mice.^460–463^ Beyond its role in Salmonella infection, NLRC4 also contributes to host defense against other enteric bacterial pathogens, such as *Citrobacter rodentium*,^464^ as well as non-enteric bacteria like *Legionella* species^465^ and *Pseudomonas aeruginosa*.^466^ Therefore, in addition to NLRP3, the interaction between NAIPs and NLRC4 is crucial for inflammasome activation in response to Gram-negative bacteria, exemplifying the complexity of the mammalian NLR network.

### PRR-dependent sensing of DAMPs

In addition to PAMPs, current understanding recognizes DAMPs can initiate immune responses by engaging an array of classical PRRs, including TLRs, NLRs, RLRs, CLRs, and diverse intracellular DNA sensors such as ALRs and cGAS.^11^ In plants, PRRs primarily localize to the plasma membrane, classified into receptor-like kinases and receptor-like proteins.^11,12^ TLR3, TLR7, and TLR9 recognize nucleic acids from damaged cells, while proteins released from cells and ECM components post-tissue injury can engage TLR2 and TLR4.^467^ A wide range of endogenous DAMPs, including HMGB1, heat shock proteins (HSPs), fibrinogen, hyaluronic acid, beta-defensins, and others, are thought to modulate TLR4 signaling either directly or indirectly.^468^ CLRs, such as OLR1, interact with multiple DAMPs such as oxidized low density lipoprotein (oxLDL),^469^ oxidized high density lipoprotein (oxHDL),^470^ apoptotic bodies,^471^ heat shock protein family D (HSPD, also known as Hsp60)^472^ and phospha-tidylserine,^473^ contributing to the pathogenesis of atherosclerosis, hypertension, and other metabolic and cardiovascular diseases.

The NLRs, take NLRP3 inflammasome as an example, in particular, is activated by diverse endogenous molecules, such as HMGB1, histones, mitochondrial DNA, mitochondrial reactive oxygen species (mtROS), cardiolipin, cold inducible RNA-binding protein (CIRBP, also known as CIRP), hyaluronic acid, monosodium urate crystals, cholesterol crystals, amyloid-β peptides and ATP.^474^ These molecules are thought to induce various intracellular damage-associated processes, such as Golgi dispersion, lysosomal rupture, and mitochondrial dysfunction, facilitating the assembly of the NLRP3 inflammasome.

In addition to their fundamental roles in virus recognition, RLRs can recognize misfolded endogenous RNAs as DAMPs, triggering signaling and potentially leading to autoimmune disorders. UPR-derived endogenous RNAs serve as pathogen-associated molecular patterns (PAMPs) that trigger RIG-I-mediated type I interferon responses in the absence of the functional SKI2 subunit of super killer complex (SKIC2, also known as ski2-like RNA helicase [SKIV2L]), which is associated with susceptibility to SLE.^475^ Furthermore, the self-RNA–RLR axis has been explored in cancer research. Recent studies demonstrated that engineered chimeric antigen receptor T (CAR-T) cells expressing RNA component of signal recognition particle 7SL1 (RN7SL1), an endogenous RNA that activates RIG-I/MDA-5 signaling, can facilitate the expansion and differentiation of CAR-T cells into effector-memory cells, thereby enhancing their antitumor efficacy.^476^ Thus, the self-RNA–RLR axis can have varying roles in tumorigenesis, acting either as pro-tumorigenic or anti-tumorigenic depending on the context.

Moreover, endogenous DNA, originating from nuclear damage or immunogenic cell death (ICD), has been demonstrated to activate cytoplasmic PRRs such as cGAS and AIM2.^477,478^ Persistent nuclear DNA damage or insufficient degradation of endogenous DNA can activate cGAS, potentially leading to autoimmune diseases including rheumatoid arthritis (RA), Aicardi–Goutières syndrome, and SLE.^479^ The translocation of a well-characterized DAMPs, HMGB1 from the nucleus to the cytoplasm can amplify cGAS-dependent STING activation.^480^ A cGAS-independent STING pathway also contributes to the recognition of DNA damage under certain conditions.^481^ Notably, endogenous DNA released during necrosis triggers AIM2-dependent inflammasome assembly, establishing a feed-forward inflammatory loop that perpetuates renal damage in chronic kidney disease.^482^

Sterile inflammation, defined by inflammatory responses initiated by DAMPs in the absence of pathogens, closely resembles pathogen-induced inflammation in its ability to activate, multiple distinct cell populations. These include non-immune cells such as epithelial, endothelial, and fibroblast cells, as well as innate and adaptive immune cells, including neutrophils, macrophages, DCs, and lymphocytes. Upon stimulation, these cells secrete pro-inflammatory cytokines and chemokines that recruit immune cells and initiate adaptive immunity.

While sterile inflammation is essential for tissue repair and regeneration, persistent or unresolved chronic inflammation can be harmful to the host and has been implicated in a range of diseases, including metabolic disorders, neurodegenerative diseases (NDDs), autoimmune conditions, and cancer. To constrain excessive activation of DAMP-sensing receptors, the immune system employs multiple regulatory mechanisms. These include PTMs, recruitment of inhibitory molecules, and degradation of receptors and adaptor proteins.

Complementary to intrinsic controls, crosstalk between PRR pathways coordinates the resolution of sterile inflammation. Together, these interconnected regulatory networks ensure a balanced and controlled inflammatory response, thereby reducing the risk of chronic inflammation and its associated pathologies.^279^

Over the past 20 years, numerous new DAMP-receptor axes have been discovered in various injury scenarios. The receptors that sense DAMPs, signaling pathways, the crosstalk between different DAMP-sensing receptors, and roles of DAMPs-PRRs in the pathogenesis of various inflammatory diseases have been extensively discussed in reviews of ours and other teams.^197–199,483^

### Inducting regulation of inflammatory mediators

The activation of PRR signaling pathways triggers the nuclear translocation of several TFs, notably NF-κB, JUND, IRFs, the RNA polymerase II transcriptional coactivator SUB1, and CCAAT enhancer-binding protein beta (CEBPB, also known as C/EBPβ).^281^ These factors synergistically regulate the transcription of target genes. In addition, chromatin remodeling plays a pivotal role in controlling the transcriptional regulation of TLR-inducible genes. Inducible nucleosome remodeling at numerous latent enhancers and several promoters shapes the transcriptional response to TLR4 signaling in macrophages. NF-κB stands out among TLR4-activated TFs due to its extensive contribution to inducible nucleosome remodeling and its capacity to activate poised enhancers and promoters within open chromatin.^284^ Furthermore, the collaboration of three variants of the switch/sucrose non-fermenting (SWI/SNF) nucleosome remodeling complex (also known as BRG1-Associated Factor [BAF] complex)—canonical BAF (cBAF), noncanonical BAF (ncBAF), and polybromo BAF (PBAF)—in macrophages is associated with de novo chromatin opening and latent enhancer activation in response to bacterial endotoxin (lipid A).^484^ However, epigenetic control of TLR-inducible genes is also crucial for the induction of tolerance to LPS, a phenomenon where cells become refractory to subsequent LPS exposure.^429,485^ Beyond cytokines, chemokines, and IFNs, PRR stimulation upregulates the expression of numerous genes in immune cells, including macrophages, DCs, and neutrophils. These genes, such as TNF, pro-IL-1β, IL-6, IL-8, IL-12, IκBζ, activating transcription factor 3 (ATF3), zinc finger CCCH-type containing 12A (ZC3H12A), and tristetraprolin, are implicated in antimicrobial defense (e.g., defensins, lipocalins), inflammatory responses, metabolic changes, and tissue repair (e.g., secretory leukocyte peptidase inhibitor [SLPI]).^486^ The proteins encoded by these genes regulate inflammatory protein expression through various mechanisms. For instance, IκBζ positively regulates the transcription of genes such as IL-6 and interleukin 12B (IL-12B, also known as IL-12p40) by interacting with NFKB1.^487,488^ Conversely, ATF3 represses target gene expression by recruiting HDACs to chromatin, thereby inducing transcriptional silencing through epigenetic modifications.^489^ ZC3H12A mediates IL-6 and IL-12B mRNA decay through its RNase activity,^490^ whereas tristetraprolin initiates TNF mRNA degradation via deadenylase recruitment and subsequent exosome-mediated decay.^491^ Despite these insights, the functions of many PRR-inducible genes remain largely unexplored. Some gene products are involved in the positive and negative regulation of inflammatory responses by modulating PRR signaling pathways. PRR-inducible genes may function as immediate regulators of innate immune signaling through feedback modulation of PRR pathways.

TLR activation triggers transcriptional upregulation of both protein-coding genes and ncRNAs, including miRNAs and lncRNAs. The 2024 Nobel Prize in Physiology or Medicine was awarded to Victor Ambros and Gary Ruvkun in recognition of their “[discovery of microRNAs and their pivotal role in post-transcriptional gene regulation].” MiRNAs regulate inflammatory responses by engaging with target mRNAs to fine-tune their expression. One of the most extensively studied miRNAs is miR-155, which is rapidly induced by TLR ligands. MiR-155 modulates both innate and adaptive immune responses by targeting and regulating multiple distinct mRNAs encoding proteins involved in inflammation and immunity. These include MYD88, C-C motif chemokine ligand 2 (CCL2/MCP1), IFN-β, Spi-1 proto-oncogene (SPI1/PU.1), TNF-α, inhibitor of nuclear factor kappa-B kinase subunit epsilon (IKBKE/IKK-i), SOCS1, miR-let-7, INPP5D, IL-1β, and others. These regulatory actions highlight the central role of miR-155 in orchestrating the immune response through intricate post-transcriptional gene regulation.^492–495^ LncRNAs can regulate target gene expression through local (cis) or non-local (trans) mechanisms. Recent research indicates that the expression of a mouse lncRNA, U90926, is highly induced in macrophages and DCs following TLR activation, mediated by p38 MAPK and MYD88. Mice lacking U90926 (U90926-KO) showed elevated serum IL-6 levels, heightened sickness responses, and increased mortality.^496^ Furthermore, a genome-wide expression analysis of TLR-activated human macrophages infected with group B Streptococcus identified another lncRNA, termed lnc-MARCKS (myristoylated alanine-rich protein kinase C substrate) or ROCKI (regulator of cytokines and inflammation). Mechanistically, ROCKI interacts with APEX1 to for a ribonucleoprotein complex at the MARCKS promoter. This complex recruits the histone deacetylase 1 (HDAC1), which removes the H3K27ac modification from the promoter, thereby suppressing MARCKS transcription and subsequently reducing Ca^2+^ signaling and inflammatory gene expression.^497^ Looking ahead, the challenges lie in deciphering the intricate mechanisms underlying these integrated networks of interactions and, crucially, assessing whether therapeutic modulation of TLR-regulated and TLR-regulating ncRNAs can benefit patients with cancer or inflammatory diseases.

### PRRs induced innate trained immunity (innate immune memory)

Over the past decade, the concept that immunological memory is exclusive to adaptive immunity has been challenged. Recent experimental evidence shows that innate immune cells can adapt following infections or vaccinations, independently of antigen specificity. This adaptation, known as trained innate immunity (TRIM) or “innate immune memory,” demonstrates that innate immune cells can exhibit memory-like properties, enhancing their responsiveness to subsequent challenges.^498^ TRIM has been observed in various mononuclear myeloid cells, including monocytes, macrophages, and DCs, as well as in lymphoid cells like NK cells and innate lymphoid cells. This phenomenon underscores a significant aspect of innate immunity, broadening our understanding of host defense mechanisms.^499^

The molecular mechanisms that allow innate immune cells to store information during TRIM primarily involve epigenetic and metabolic processes. These modifications remodel chromatin architecture and DNA methylation patterns, directly modulating transcriptional activity. Innate immune cells detect PAMPs and DAMPs through PRRs such as dectin-1 for β-glucans and NOD2 for peptidoglycans.^500^ This detection activates intracellular pathways, leading to transcription factors like JUND and C/EBP, which trigger the transcription of genes critical for antimicrobial defense, including cytokines, chemokines, and defensins. For instance, bacille calmette-guérin (BCG) vaccination boosts monocyte function in healthy volunteers through NOD2 receptor-dependent histone H3 lysine 4 trimethylation (H3K4me3), enhancing activation markers such as CD11b and TLR4.^501^ Similarly, the β-glucan component of fungal cell walls activates aerobic glycolysis via the AKT–HIF1A–mTOR pathway, a process that can be inhibited chemically to block TRIM induction.^502^ Concurrently, the tricarboxylic acid cycle is repurposed to support anabolic functions like cholesterol synthesis, providing metabolites such as mevalonate that enhance TRIM responses.^503^ While transcriptional, epigenetic, and metabolic reprogramming have been recognized as foundational to TRIM in hematopoietic stem cells (HSCs) and peripheral innate immune cells, the detailed molecular mechanisms remain partially understood and warrant further investigation.

### PRRs in autoimmune diseases

Under homeostatic conditions, PRRs maintain immune tolerance by precisely distinguishing self from PAMPs, thereby preventing autoantibody production and autoimmune pathogenesis. Extensive research has implicated PRRs in the pathogenesis of multiple distinct autoimmune diseases, including inflammatory bowel disease, RA, SLE, psoriasis, and multiple sclerosis.^11,112^ In the case of SLE, autoantibody-immune complex deposition in the kidneys leads to glomerular nephritis. TLRs, notably TLR7 and TLR9, have been shown to contribute to autoantibody production in both mouse models and patient cohorts. Normally, self-nucleic acids released from damaged cells are swiftly degraded by serum nucleases, preventing their interaction with TLR7 or TLR9.^504^ However, when these self-nucleic acids bind to cellular proteins such as HMGB1, ribonucleoproteins, antimicrobial peptides, and autoantibodies, the resulting nucleic acid-protein complexes are more easily endocytosed. This process facilitates endosomal uptake of nucleic acids, shielding them from nuclease degradation and subsequently inducing TLR7- and TLR9-mediated production of IFN-Is and TNF-α in diverse immune cells, such as B cells and pDCs.^344,505^ The IFN-Is and cytokines triggered by TLRs trigger inflammation, creating a positive feedback loop that exacerbates autoimmune disease progression. Consequently, the development of selective TLR7/8/9 inhibitors has become a focal point of research, with several compounds currently undergoing clinical trials for the treatment of autoimmune diseases.^506^

cGAS, an enzyme that detects both foreign DNA and aberrantly located self-DNA (pathogenic self-DNA) within intracellular compartments. Approximately 5% of the global population is afflicted by autoimmune diseases, many of which exhibit an IFN signature linked to the accumulation of cytoplasmic DNA. James Chen, along with Dan Stetson from the University of Washington working independently, demonstrated that the DNA accumulation observed in mice deficient in three prime repair exonuclease 1 (TREX1)—an enzyme responsible for degrading cytoplasmic DNA, including that derived from damaged organelles such as mitochondria or nuclei—mediates its pathogenic effects through cGAS. Specifically, they showed that a deficiency in cGAS rescues the lethality associated with TREX1 deficiency.^507,508^ These compelling findings indicate that the accumulation of cytoplasmic DNA in cells can activate the cGAS–STING pathway, leading to an IFN response and potentially predisposing individuals to autoimmunity, as demonstrated in these mouse models. Given the critical role of this pathway in immune regulation, it is unsurprising that the inhibition of cGAS has become a key focus in inflammatory biology and biomedical research.

Furthermore, PRRs additionally identify tumor-originated DNA, thereby initiating antitumor immune responses. Considering the dual, yin-yang nature of the immune system, there has been considerable interest in agonizing PRRs to enhance the immune response against cancer, rather than inhibiting them. Consequently, PRRs have emerged as promising targets for cancer therapy.^509^ This is particularly evident in the field of cancer immunotherapy, where the agonism of PRRs and their signaling pathways through DNA may act as an adjuvant, stimulating an antitumor response akin to checkpoint inhibition.^510^ For instance, the activation of NOP2/Sun RNA methyltransferase 2 (NSUN2) maintains global 5-methylcytosine (m5C) RNA methylation, including that of TREX2, stabilizing it and thereby restricting cytosolic dsDNA accumulation and cGAS/STING activation. This, in turn, promotes tumorigenesis and resistance to anti-programmed cell death-ligand 1 (PD-L1) immunotherapy in CT26 colon and B16 melanoma cancer cells.^511^ However, it’s important to recognize that DNA sensing can have both pro- and antitumor effects, which depend on the stage of tumorigenesis. This complex impact often varies based on the type of cancer, the stage of progression, and the specific therapy being considered. Current research in this area is extensive, and the expected outcomes are both insightful and exciting.

In summary, the dysfunctional or abnormal PRR-dependent recognition of self-nucleotides is implicated in the pathogenesis of numerous inflammatory and autoimmune diseases. This dysregulation presents an opportunity for the development of therapeutic strategies, with PRRs serving as promising targets for the design of specific agonists or antagonists.

### PRR activation in other sterile inflammation with acute and chronic disease courses

Inflammation can be sterile or infectious. In infections, exogenous PAMPs or endogenous DAMPs activate PRRs, triggering acute inflammation that can escalate to septic shock. Chronic inflammation, however, involves a persistent low-grade response, often leading to tissue damage, fibrosis, and disease progression, and is commonly associated with autoimmune disorders, cancer, and degenerative diseases. While section “PRR-mediated pathogen recognition,” discusses PRRs in infectious inflammation, this section focuses on PRR regulation in sterile inflammation.

It is well known that PRRs-dependent inflammation contributes significantly to the pathogenesis of ischemia-reperfusion injury in multiple organs. Research highlights the critical involvement of TLRs in DAMP recognition during I/R. Activation of TLRs, including, including TLR2,^512^ TLR4,^468^ TLR3,^513^ and TLR9,^514^ has been linked to enhanced inflammation and tissue injury. Mice lacking TLR2, TLR4, or MYD88 show reduced infarcted regions in response to cardiac as well as cerebral ischemic-reperfusion injury.^515–517^ For instance, biglycan, a small leucine-rich proteoglycan, interacts with TLR4 and its coreceptor CD44 to induce autophagy in macrophages, contributing to renal inflammation and damage during I/R.^518^ Genetic deletion or pharmacological inhibition of these receptors has been shown to reduce systemic inflammation and protect against tissue damage in various I/R models, including those affecting the liver, kidneys, and intestines.^519–521^

Chronic inflammation, coupled with aberrant mechanisms of tissue repair and remodeling, contributes to the pathogenesis of tissue fibrosis. This condition is marked by the abnormal and excessive deposition of ECM components, which impair the function of tissues or organs and, in severe instances, can lead to mortality.^522^ PRRs, serving as key inflammatory mediators, disrupt the intricate balance between inflammatory responses and tissue regeneration, thereby propelling the remodeling processes that typify fibrosis. Recent literature delineates the role of various PRRs, such as TLRs, CLRs, NLRs, and non-PRRs such as advanced glycosylation end product specific receptor (AGER/RAGE).^420,523–527^ Such interactions initiate immune responses and elicit profibrotic reactions in non-immune cells such as hepatocytes,^528,529^ epithelial cells,^530^ ECs,^531^ HSCs,^532^ and fibroblasts.^533^ DAMPs directly activate fibroblasts through PRRs, which in turn promote the activation of myofibroblasts, a process central to fibrosis. S100 calcium binding protein A4 (S100A4) is a critical DAMP that is released upon cell damage or stress and is significantly associated with fibrosis and mediates its effects through TLR4 or AGER.^534^ Furthermore, PRRs regulate various innate immune cells, including innate lymphoid cells, pDCs, Kupffer cells, and macrophages, to secrete cytokines and chemokines that further recruit cells, sustain inflammation, and enhance profibrotic responses.^522,535^

This inflammation activation leads to the secretion of a wide range of cytokines, such as TNF, IL-1A, IL-1B, IL-17, as well as various chemokine, including CCL5, C-X-C motif chemokine ligand 1 (CXCL1), and others. In addition, adhesion molecules, such as intercellular adhesion molecule-1 (ICAM-1), vascular cell adhesion molecule-1 (VCAM-1) and E-selectin, growth factor, antigens such as CD40 and other inflammatory associated proteins such as tissue factor, nitric oxide synthase 2 (NOS2, also known as inducible nitric oxide synthase [iNOS]), mucin 8, inhibitor-SOCS1 are also produced.

The molecular mechanisms driving the potential pro-inflammatory effects of PRR involve the activation of several signaling pathways, including adaptor proteins MYD88 and TICAM1, MAPKs and IKK, transcriptional factors NF-κB, TBK1, phosphorylation of SYK, NLRP3 inflammasome signaling, STING, p38, MAPK8, PI3K/AKT, JAK, SRC proto-oncogene, non-receptor tyrosine kinase (SRC), ETS transcription factor ELK1 (ELK1), sirtuin 1 (SIRT1), and STATs.^536^ Suppression of TLR4-MYD88 signaling pathway attenuated chronic mechanical pain caused by neuroinflammatory cascades in a rat model of endometriosis.^537^ Halting STAT1 activity mitigates HMGB1 engagement and enhances the condition of chronic kidney inflammation, thus spotlighting the STAT1-HMGB1-NF-κB pathway as an innovative therapeutic objective in managing cisplatin nephrotoxicity.^538^

Therapeutic strategies targeting PRRs or their ligands in chronic inflammation have demonstrated potential benefits. However, selectively fine-tuning PRR activity, rather than complete inhibition, may offer greater advantages. Achieving this requires a comprehensive understanding of the interactions among cells during inflammation and tissue regeneration, as well as the PRR-mediated signaling pathways. Such insights are crucial for effectively managing excessive inflammation, resolving chronic inflammation and fibrosis, and promoting tissue repair and regeneration.

### PRR regulation in carcinogenesis

Dysregulated innate and adaptive immunity is exhibited by at least one-third of cancers associated with chronic inflammation, which are central to initiating and sustaining a chronic inflammatory state within the tumor microenvironment (TME). This inflammatory environment influences nearly every stage of carcinogenesis and significantly affects cancer therapy outcomes.^539,540^ Over the past two decades, innate immune PRRs have emerged as essential regulators, modulating a wide range of cellular responses in both immune cells within the TME and directly within cancer cells, with effects that can either inhibit or promote tumor growth.

**Fig. 13:**
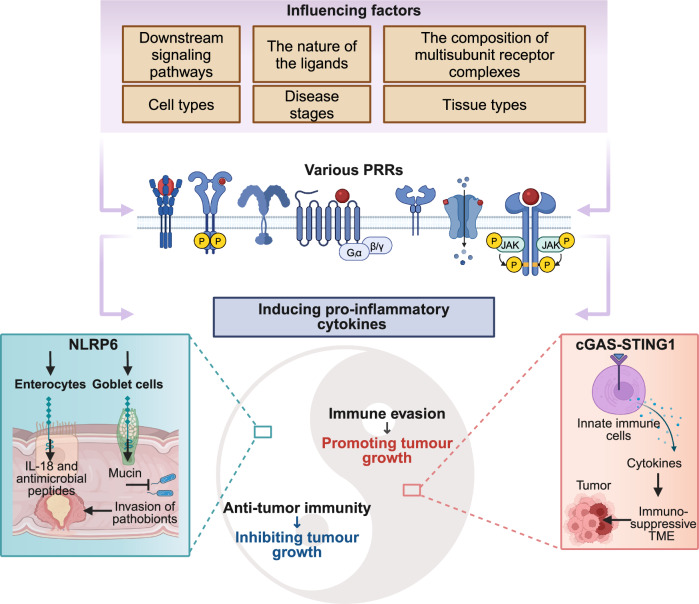
PRR regulation in carcinogenesis. The functional pleiotropy of PRRs in carcinogenesis is likely governed by cell type, the nature of the upstream activating ligand, the composition of multisubunit receptor complexes, downstream signaling pathways, disease stage, and tissue type. PRR-induced pro-inflammatory cytokines play dual roles in tumor immunity, including antitumor immunity to inhibit tumor growth and immune evasion to promote tumor growth. For example, NLRP6 regulates the secretion of IL-18 and antimicrobial peptides in enterocytes, as well as mucin production in goblet cells, which contributes to its ability to prevent the invasion of pathobionts that initiate colitis-associated colorectal cancer, thereby inhibiting tumor growth. The functional cytokine introduce by cGAS–STING pathway activates the production of anti-inflammatory cytokines, which contribute to an immunosuppressive TME. This figure is created by BioRender (https://app.biorender.com)

The functional pleiotropy of PRRs is likely governed by multiple factors, including cell type, the nature of the upstream activating ligand (e.g., microbial vs. host-derived molecules), the composition of multisubunit receptor complexes, downstream signaling pathways, disease stage, and tissue type^539^ (Fig. 13). In terms of cell type, PRRs expressed in immune cells exert potent immunomodulatory functions that influence both early and late stages of cancer. Conversely, PRRs in epithelial, endothelial, and fibroblast cells orchestrate various cancer-associated processes that are typically considered non-immune-related. For instance, in the *gp130*^*F/F*^ knock-in mouse model of spontaneous gastric inflammation-associated cancer, driven by JAK-STAT3-mediated TLR2 upregulation, TLR2 did not regulate inflammation.^541,542^ Instead, it functioned within gastric epithelial cells to enhance the expression of genes (e.g., cyclin D2 [CCND2] and B-cell lymphoma 2 related protein A1a [BCL2A1A]) and activate signaling pathways (e.g., NF-κB, PI3K-AKT, and ERK-MAPK) associated with cell proliferation and survival.^541^ The inflammation-independent role of TLR2 in promoting cancer cell proliferation in the gastrointestinal tract has been demonstrated in vivo using human tumor xenografts in immunocompromised or “humanized” mice models of gastric and pancreatic cancers.^543,544^ Furthermore, the diverse non-immune functions of TLRs in cancer are largely recapitulated by NLRs. For example, the tumor-inhibitory functions of inflammasomes containing NLRP6, one of the most abundantly expressed PRRs in the colon, include maintaining epithelial barrier integrity through intestinal epithelium self-renewal.^545^ NLRP6 also regulates the secretion of IL-18 and antimicrobial peptides in enterocytes,^546^ as well as mucin production in goblet cells,^547^ which contributes to its ability to prevent the invasion of pathobionts that initiate colitis-associated colorectal cancer^548^ (Fig. 13).

The combination of genetically defined spontaneous and induced murine tumor models with whole-organism or cell-type-specific conditional genetic approaches, such as gene knockouts, has unveiled diverse and frequently contrasting roles of PRRs both within individual cancer types and across different cancers. Utilizing AIM2^−/−^ mice in a diethylnitrosamine-induced hepatocellular carcinoma model, research has demonstrated that AIM2 inflammasome activation in Kupffer cells promotes inflammation-associated tumor initiation in the liver; conversely, AIM2 deficiency diminishes hepatocellular carcinoma development in these mice.^549^ In contrast, studies employing mouse models of sarcomas, which are highly immunogenic, and melanoma, which is relatively non-immunogenic, have shown that STING activation in innate immune cells, including myeloid cells and DCs, inhibits tumorigenesis. This occurs through the enhancement of T-cell-mediated or NK cell-mediated antitumor immunity, respectively.^550^

In oncogenesis, PRRs critically mediate both transcriptional activation and post-translational modification of inflammatory cytokines within innate immune cells. Members of the IL-6 family (such as IL-6 and IL-11), TNF, inflammasome-associated IL-1 family cytokines (like IL-1β and IL-18), and IFN-I are included. Once released from immune cells, cancer cells are directly affected by these cytokines.. For example, IL-6 stimulates proliferation, survival, invasion, and metastasis,^551^ while TNF can either trigger apoptosis and necroptosis in tumor cells or, conversely, extend their survival.^552^ The production of anti-inflammatory cytokines, such as IL-10 and TGF-β, further modulates the functional consequences of these cytokine activities, contributing to an immunosuppressive TME^553,554^ (Fig. 13). These inflammatory mediators also function in an autocrine or paracrine manner on various cells within the TME, including other immune cells, fibroblasts, ECs, and epithelial (as well as tumor) cells. They direct immunomodulatory processes that can either establish a TME that is chronically inflamed and immunosuppressive, thereby supporting tumorigenesis, or enhance adaptive immune-mediated antitumor immunity. Consequently, PRR-induced pro-inflammatory cytokines and IFNs play dual roles in cancer (Fig. 13).

Unlike the advancements seen in immunotherapy, where our comprehension of the adaptive immune system’s significance in antitumor immunity has prompted the application of immune checkpoint inhibitors across various cancers, the therapeutic potential of innate immunity in oncological contexts remains largely unexplored. Preclinical investigations have unveiled an intricate scenario, wherein particular PRRs demonstrate contrasting roles, either inhibiting or promoting tumor growth, and numerous PRRs exhibit substantial functional overlap across certain cancer types. Consequently, these observations underscore the necessity for caution when considering the clinical deployment of specific PRR agonists or antagonists in individual cancer cases. Crucially, a profound understanding of a given PRR’s activity within a specific TME is imperative to prevent unintended enhancement of tumor-promoting immunosuppression or chronic inflammation upon administration of these modulators. This knowledge will facilitate the judicious selection and application of PRR-targeted therapies, ensuring they contribute positively to cancer treatment strategies.

### The role of PRR in aging

**Fig. 14:**
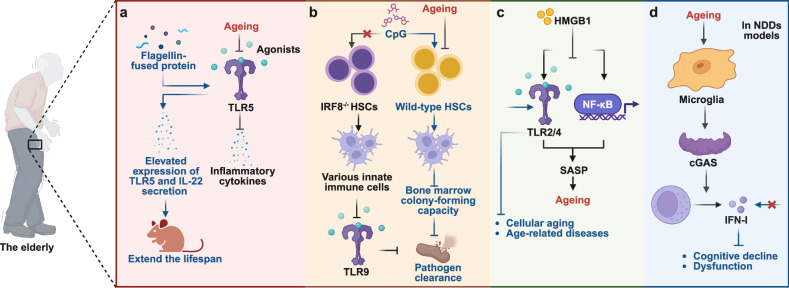
The role of PRR in ageing. **a** Stimulation of TLR5 by a flagellin-fused protein has been shown to effectively extend the lifespan and enhance the health span of mice of both genders. This effect is mediated by an increased surface expression of TLR5 and a subsequent elevation in IL-22 secretion. Additionally, aging diminishes TLR activation by agonists, leading to decreased production of inflammatory cytokines. **b** Hematopoietic stem cells (HSCs) lacking IRF8 do not respond to CpG, an agonist of TLR9 in mice. In contrast, continuous CpG stimulation not only increases the number of wild-type HSCs but also reduces their ability to form bone marrow colonies in ageing mice. **c** Extracellular HMGB1 stimulates the release of inflammatory cytokines through the TLR2/4 and NF-κB signaling pathways, thereby enhancing the production of the senescence-associated secretory phenotype (SASP). Blocking TLR2 and TLR4 can alleviate cellular aging and mitigate age-related diseases. **d** Inhibiting IFN-I response mediated by cGAS activation mitigates aging-associated cognitive decline and dysfunction in neurodegenerative diseases (NDDs) models. This figure is created by BioRender (https://app.biorender.com)

Age-related immunological changes are diverse and encompass heightened vulnerability to infections and persistent tissue inflammation.^555^ The decline in PRR signaling pathways plays a pivotal role in the aging-dependent degradation of innate immune cell function, impairing their ability to defend against infections and respond adequately to stimuli. Specifically, aging diminishes TLR activation by agonists, leading to decreased release of inflammatory cytokines such as TNF-α, IL-6, and IL-12, owing to reduced TLR expression.^556^ Targeting specific TLRs presents a potential therapeutic avenue for addressing aging-associated immune deficiencies. Among various TLRs, TLR5 stands out as its expression and signaling remain relatively intact in aged mice and elderly humans.^557^ Stimulation of TLR5 through mucosal administration of a flagellin-fused protein has been shown to effectively extend the lifespan and enhance the health span of mice of both genders. This effect is mediated by boosting intestinal mucosal integrity, achieved through increased surface expression of TLR5 on a specific subset of DCs and elevated IL-22 secretion^558^ (Fig. 14a).

HSCs are the progenitors of all cells within the hematopoietic system. As such, age-associated cellular and molecular changes in HSCs play a pivotal role in the functional decline of this system. In addition, TLRs exert regulatory functions in HSC development. In a direct sensing mechanism, HSCs themselves detect inflammatory cytokines or microbial products through cytokine receptors and PRRs, including TLRs, expressed on their surface. This response by HSCs to infection can aid in pathogen clearance. However, growing evidence indicates that it may also result in HSC exhaustion and functional compromise. For instance, IRF8^−/−^ HSCs fail to respond to CpG, a TLR9 agonist in mice, whereas prolonged CpG stimulation enhances the abundance of wild-type HSCs while diminishing their bone marrow colony-forming capacity. Mechanistically, various innate immune cells, including DCs, macrophages, and NK cells, exhibit disrupted TLR9 signaling due to developmental defects in IRF8^−/−^ mice^559^ (Fig. 14b). Given the intimate connection between HSC function and immunosenescence, therapeutic approaches targeting TLRs in the context of HSC aging hold considerable clinical promise.

PRRs also regulate cell senescence in ageing process through their binding ligands and subsequent signaling pathways. For example, DAMPs such as extracellular histones can trigger inflammation and senescence in vascular smooth muscle cells by activating the NLRP3 inflammasome and the AMP-activated protein kinase (AMPK)-forkhead box O4 (FOXO4) pathway, thus exacerbating histone-induced damage in mice.^560^ Senescent cells contribute to age-related tissue dysfunction, in part, through the establishment of a senescence-associated secretory phenotype (SASP). Extracellular HMGB1 triggers the secretion of inflammatory cytokines via the TLR2/4 and NF-κB signaling pathways, facilitating the production of secreted SASP.^561,562^ Blocking PRRs, such as TLR2 and TLR4, can alleviate cellular aging and age-related diseases,^563^ including osteoarthritis, vascular dysfunction in aged mice,^564^ and hair follicle cycle and regeneration^565^ (Fig. 14c).

Aging stands as the primary risk factor for NDDs, a class of progressive neurological disorders characterized by devastating outcomes.^566^ Reports have documented various mechanisms of innate immune activation in neurodegeneration, with a particular focus on PRRs that detect DAMPs linked to neurodegenerative processes. These PRRs, along with other DAMP-sensing receptors, are abundantly expressed in myeloid cells, notably microglia—the resident macrophages and key mediators of the innate immune response. Such receptors are also found in ECs, oligodendrocytes, astrocytes, and even neurons at certain functional levels.^567^ Among the PRRs implicated in neurodegeneration, TLRs, CLRs, NLRs, and ALRs forming inflammasomes are the most well-characterized. Furthermore, a robust IFN-I response mediated by cGAS activation has been observed in several NDDs during their progression, likely triggered by cellular stress or death.^568^ Compelling evidence suggests that inhibiting this IFN-I response mitigates aging-associated cognitive decline and dysfunction in NDD models^569,570^ (Fig. 14d). Pro-inflammatory mediators, including IL-1β, IL-6, and TNF-α, which are produced by the activation of PRRs and other DAMP-sensing receptors, have been identified in postmortem brain tissue, serum, and cerebrospinal fluid of patients with various NDDs.^571^ In the early stages of the disease, these mediators enhance and prolong inflammatory responses via non-cell-autonomous mechanisms. They activate inflammatory pathways in neighboring microglia, astrocytes, vascular, and parenchymal cells. Moreover, the chronic production and release of neuroinflammatory mediators create a vicious cycle that disrupts synaptic balance, induces cellular apoptosis, and overactivates pathways involved in the processing and aggregation of key proteins associated with pathological aging and neurodegeneration.^572^ To deepen our understanding of PRRs’ role in NDDs, we recommend reading recent reviews such as “Innate Immune Activation in Neurodegenerative Diseases”,^566^ “Targeting the NLRP3 Inflammasome for Neurodegenerative Disorders”^573^ and “The Role of Toll-like Receptors and Neuroinflammation in Parkinson’s Disease”.^574^

To sum up, PRRs detect both external and internal danger signals. They are crucial in chronic inflammation and immune dysregulation, which are main characteristics of aging and related diseases. Their capacity to trigger varied signaling pathways shows they both push forward and adjust the aging process.

## PRRs-targeting therapeutic strategies

The significant role of PRRs in innate immunity has garnered considerable attention within immunology and drug research domains.^566,575,576^ PRRs encompass a diverse array of receptor types and ligands, along with numerous signaling pathways triggered by receptor-ligand interactions. These elements serve as pivotal drug targets for addressing tumors, inflammation, autoimmune disorders, pathogenic infections, vaccine development, and other conditions, marking them as essential avenues for immunotherapy. The activation of PRRs presents a dual-edged sword: while it stimulates both innate and adaptive immune responses to combat pathogens, it also induces the production of numerous cytokines, creating an inflammatory milieu that can lead to tissue damage. Consequently, therapeutic strategies targeting PRRs primarily involve the use of ligand analogs to activate these receptors,^577^ using antagonists to block their activation, or using antibodies and small molecules to inhibit PRR signaling pathways.^578^ An overview of the expanding repertoire of PRR-directed agonists and antagonists currently in clinical stages of development is provided by Table 3. Key examples are discussed subsequently, with a particular emphasis on applications in infection, sterile inflammation, and cancer.

**Table 3:** Treatment with PRRs’ agonists and antagonists in clinical trials

Disease type	Category of PRRs	Agonists	Study type/phase	ClinicalTrials.gov identifier
Cancers				
Acute myelogenous leukemia	TLRs	GNKG168	Interventional/phase 1	NCT01743807
Acute lymphoblastic leukemia	TLR7/8	Imiquimod	Interventional/phase 2	NCT03559413
Breast cancer	TLR7	Imiquimod	Interventional/phase 2	NCT01421017 NCT00899574 NCT03982004 NCT00821964
	TLR7	852 A	Interventional/phase 2	NCT00319748
	TLR7/8	BDC-1001	Interventional/phase 2	NCT05954143
Colorectal cancer	TLR7	Imiquimod	Interventional/phase 3	NCT00785122 NCT02135419 NCT00785122 NCT02600949
	TLR9	IMO 2055	Interventional/phase 2	NCT00719199
Cutaneous T-cell lymphoma	TLR3	Poly ICLC	Interventional/phase 1	NCT02061449 NCT01976585
	TLR4	G100 agent	Interventional/phase 2	NCT03742804
	TLR7	Imiquimod	Interventional/early phase 1	NCT03116659 NCT02301494 NCT05838599,
	TLR7/8	Resiquimod	Interventional/phase 1	NCT01676831
Mantle cell lymphoma	TLR9	PF-03512676	Interventional/phase 2	NCT00490529
B-cell lymphoma	TLR9	SD-101	Interventional/phase 2	NCT02254772 NCT02927964
Chronic lymphocytic leukemia	TLR7	Imiquimod	Interventional/phase 2	NCT00596336
Endometrial cancer	TLR7	852 A	Interventional/phase 2	NCT00319748
Head and neck cancer	TLR5	Entolimod	Interventional/phase 1	NCT01728480
	TLR8	Motolimod	Interventional/phase 1	NCT03906526
Pancreatic cancer	TLR3	Rintatolimod	Interventional/phase 1	NCT05927142
	TLR9	SD-101	Interventional/phase 1	NCT04050085
Melanoma	TLR3	Poly-ICLC	Interventional/phase 2	NCT04364230
	TLR	MEL60	Interventional/phase 2	NCT02126579
	TLR4	GLA-SE	Interventional/early phase 1	NCT02320305
	TLR7/8	Resiquimod	Interventional/phase 2	NCT00960752 NCT01748747
	TLR7/8	Imiquimod	Interventional/phase 1	NCT04072900 NCT03276832 NCT00453050 NCT00651703 NCT00273910 NCT00273910 NCT01264731 NCT01191034 NCT00118313 NCT00470379 NCT00821652
	TLR9	ixatimod	Interventional/phase 2	NCT05061017
	TLR	MEL58	Interventional/phase 1	NCT01585350
Lentigo malignant	TLR7/8	Imiquimod	Interventional/phase 3 and phase 4	NCT02394132 NCT01161888 NCT01720407 NCT01088737 NCT00707174
Oral cancer	TLR7/8	Imiquimod	Interventional/early Phase 1	NCT04883645
Ovarian cancer	TLR3	Ampligen	Interventional/phase 2	NCT01312389
	TLR7	Imiquimod	Interventional/phase 2	NCT00799110
	TLR7	852 A	Interventional/phase 2	NCT00319748
	TLR8	VTX-2337	Interventional/phase 1	NCT01294293 NCT01666444 NCT02431559
Vulvar intraepithelial neoplasia	TLR7/8	Imiquimod	Interventional/phase 3	NCT01861535 NCT00941811
Cervical intraepithelial neoplasia	TLR7/8	Imiquimod	Interventional/NA	NCT03206138, NCT02864147 NCT02329171 NCT02917746 NCT02669459 NCT02130323 NCT01283763 NCT00988559 NCT00941252 NCT00788164 NCT00031759 NCT06356012 NCT03196180 NCT05405270 NCT03233412 NCT04859361
Cervical cancer	TLR7	852 A	Interventional/phase 2	NCT00319748
	TLRs	VTX-2337	Interventional/phase 1	NCT01294293
Peritoneal carcinomatosis	TLR3	Ampligen	Interventional/phase 2	NCT01312389
	TLR8	VTX-2337	Interventional/phase 1	NCT01294293 NCT01666444
Fallopian tube cancer	TLR3	Ampligen	Interventional/phase 2	NCT01312389
	TLR8	VTX-2337	Interventional/phase 2	NCT01666444 NCT01294293
Lung cancer	TLR7	BNT411	Interventional/phase 2	NCT04101357
	TLR7/8	EIK1001	Interventional/phase 2	NCT06246110
	TLR7/8	Imiquimod	Interventional/phase 2	NCT01909752
	TLR9	DV281	Interventional/phase 1	NCT03326752
Bladder cancer	TLR7/8	Imiquimod	Interventional/phase 1	NCT05375903 NCT01731652
Glioblastoma	TLR3	Poly-ICLC	Interventional/phase 2	NCT02754362
	TLR7	Imiquimod	Interventional/phase 1	NCT01403285 NCT02078648 NCT01808820
Cutaneous neurofibromas	TLR7	Imiquimod	Interventional/phase 1	NCT00865644
Recurrent gliomas	TLR7/8	Imiquimod	Interventional/early Phase 1	NCT01678352 NCT04808245
Prostate cancer	TLR7	Imiquimod	Interventional/phase 2	NCT02452307 NCT02234921
	TLR7/8	STM-416p	Interventional/phase 1	NCT06450106
	TLR9	SD-101 Vidutolimod	Interventional/phase 2	NCT03007732 NCT05445609
Soft tissue sarcomas	TLR4	GLA-SE	Interventional/phase 1	NCT02180698
	TLR7	Imiquimod	Interventional/phase 1	NCT01803152
Basal cell carcinoma	TLR7/8	Resiquimod	Interventional/phase 2	NCT01808950 NCT00079300 NCT00066872
	TLR7/8	Imiquimod	Interventional/phase 3	NCT05212246 NCT06252857 NCT00803907 NCT03534947 NCT02242929 NCT00463359 NCT00314756 NCT00204555 NCT00189306 NCT00189280 NCT00189241 NCT00129519 NCT00581425
Malignant glioma	TLR3	polyICLC (Hiltonol^®^)	Interventional/phase 2	NCT01204684
	TLR7/8	Imiquimod	Interventional/phase 1	NCT01792505 NCT06305910
Squamous cell cancer	TLR8	VTX-2337	Interventional/phase 1	NCT01334177
	TLR7/8	Imiquimod	Interventional/phase 4	NCT01926496 NCT03370406 NCT00384124
	TLR9	EMD 1201081	Interventional/phase 2	NCT01040832
Solid tumors	TLR3	Poly-ICLC	Interventional/phase 2	NCT02643303 NCT02661100
	TLR5	CBLB502	Interventional/phase 1	NCT01527136
	TLR7	Imiquimod	Interventional/phase 1	NCT03872947 NCT02276300 NCT02224599
	TLR7	DSP-0509	Interventional/phase 2	NCT03416335
	TLR8	VTX-2337	Interventional/phase 1	NCT02650635
	TLR7/8	BDB018	Interventional/phase 1	NCT04840394
	TLR7/8	Resiquimod	Interventional/phase 2	NCT00948961
	TLR7/8	BDC-1001	Interventional/phase 2	NCT04278144
	TLRs	DECOY20	Interventional/NA	NCT05651022
Infection				
COVID-19	TLR4	MPL	Interventional/early Phase 1	NCT04523246
HIV infections	TLR7	Vesatolimod	Interventional/phase 2	NCT06071767 NCT04364035
	TLR8	Selgantolimod	Interventional/phase 2	NCT05551273
	TLR7/8	3M-052-AF	Interventional/phase 1	NCT06613789 NCT06267872 NCT04177355
	TLR9 TLR7/8	TEACH (TLR9) Imiquimod (TLR7/8)	Interventional/phase 2 Interventional/phase 3	NCT02443935 NCT02059499 NCT01663558
Influenza	TLR7	Imiquimod	Interventional/phase 3	NCT02103023 NCT02960815 NCT01508884 NCT05315024 NCT04143451 NCT03472976 NCT02103023
	TLR7/8	Resiquimod	Interventional/phase 1	NCT01737580
Chronic HBV infection	TLR7/8	Imiquimod	Interventional/phase 3	NCT02621112 NCT04083157
	TLR8	TQA3810	Interventional/phase 2	NCT06566248 NCT06431438
Herpes zoster infection	TLR7/8	Imiquimod	Interventional/phase 3	NCT03073967
	TLR9	CpG 1018	Interventional/phase 1	NCT05245838
HPV infection	TLR7/8	Imiquimod	Interventional/phase 3	NCT03289260, NCT02689726 NCT03180684
Sepsis	TLR	Mw	Interventional/phase 3	NCT02025660 NCT00184990 NCT02554630
Anthrax	TLR9	CPG 7909	Interventional/phase 2	NCT05997264
Parasites				
Hookworm	TLR9	CpG 10104	Interventional/phase 1	NCT03172975 NCT02143518
Plasmodium	TLR7/8	Imiquimod	Interventional/early phase 1	NCT05468606
Respiratory diseases				
Asthma	TLR3	Poly ICLC	Interventional/NA	NCT02090374
	TLR7	GSK2245035	Interventional/phase 2	NCT01788813 NCT03707678 NCT01607372 NCT02833974 NCT01480271
Allergic rhinitis	TLR4	INI-200	Interventional/phase 1	NCT06038279
Respiratory tract infection	TLR7/8	Resiquimod	Interventional/phase 2	NCT05421325
Digestive diseases				
Ulcerative colitis	TLR9	Cobitolimod	Interventional/phase 3	NCT04985968
Cirrhosis	TLR7/8	Imiquimod	Interventional/phase 2	NCT05028322
Chronic hepatitis B	TLR7/8	Resiquimod	Interventional/phase 2	NCT00175435
	TLR7/8	Imiquimod	Interventional/phase 3	NCT03307902
	TLR8	Selgantolimod	Interventional/phase 2	NCT05551273
	TLR9	SD-101	Interventional/phase 1	NCT00823862
Chronic hepatitis C	TLR9	IMO-2125	Interventional/phase 1	NCT00728936 NCT00990938
Autoimmune diseases				
Cutaneous lupus erythematosus	TLR7/8	Imiquimod	Interventional/NA	NCT06411106
Skin diseases				
Photoaging	TLR7/8	Imiquimod	Interventional/NA	NCT01935310
Keloid	TLR7/8	Imiquimod	Interventional/phase 2	NCT03760250
Birthmarks	TLR7/8	Imiquimod	Interventional/NA	NCT00585247
Vascular malformations	TLR7/8	Imiquimod	Interventional/phase 2	NCT00979550
Alopecia areata	TLR7/8	Imiquimod	Interventional/phase 4	NCT00176943 NCT00177021
Actinic cheilitis	TLR7/8	Imiquimod	Interventional/phase 4	NCT00849992 NCT04219358 NCT02281682 NCT05740969
	TLR7/8	Resiquimod	Interventional/phase 2	NCT01583816
Aktinic keratosis	TLR7/8	Imiquimod	Interventional/phase 4	NCT04842422 NCT03914417
Plaque-type morphea	TLR7/8	Imiquimod	Interventional/phase 3	NCT00230373
Genital warts	TLR7/8	Imiquimod	Interventional/phase 3	NCT03901690 NCT02482428 NCT00189293 NCT00735462 NCT00674739 NCT00761371
Plantar warts	TLR7/8	Imiquimod	Interventional/phase 4	NCT01059110 NCT05146895
Common warts	TLR7/8	Resiquimod	Interventional/phase 2	NCT00117923 NCT00115141 NCT00114920
Cutaneous Leishmaniasis	TLR7/8	Imiquimod	Interventional/phase 2	NCT01380314 NCT00257530
Verruca plana	TLR7/8	Imiquimod	Interventional/phase 2	NCT05314127
Vulvar Paget’s disease	TLR7/8	Imiquimod	Interventional/phase 3	NCT02385188 NCT00504023
Psoriasis	TLR7/8	Imiquimod	Interventional/NA	NCT00470392
Actinic keratoses	TLR7/8	Imiquimod	Interventional/phase 4	NCT00777127 NCT01453179 NCT02362152 NCT01788007 NCT01686152 NCT01611480 NCT01538901 NCT01502020 NCT01413763 NCT01229319 NCT00948428 NCT00894647 NCT00859105 NCT00828568 NCT00814528 NCT00774787 NCT00605176 NCT00603798 NCT00335179 NCT00294320 NCT00189267 NCT00175643 NCT00116649 NCT00115154 NCT00114023 NCT00110682 NCT01203878 NCT00189254 NCT02120898 NCT04809662
Chronically photodamaged skin	TLR7/8	Imiquimod	Interventional/phase 4	NCT02889159
Neurological disorders				
Cognitive impairment	Tlr2/4	Agonists	Interventional/NA	NCT05859230
Pediatric/neonatal diseases				
Schistosomiasis	TLR4	GLA-SE	Interventional/phase 2	NCT03799510 NCT03041766
Common warts	TLR7/8	Resiquimod	Interventional/phase 2	NCT00116675 NCT00116662
Plaque morphea	TLR7/8	Imiquimod	Interventional/phase 3	NCT00147771
Ependymomas	TLR7/8	Imiquimod	Interventional/phase 1	NCT01795313
Pediatric brain tumors	TLR7/8	Imiquimod	Interventional/phase 1	NCT01902771 NCT01171469 NCT01400672
Hemangioma	TLR7/8	Imiquimod	Interventional/phase 2	NCT00601016
Healthy subjects	TLR4	GSK1795091	Interventional/phase 1	NCT02798978
	TLR7/8	Imiquimod	Interventional/phase 1	NCT03086278
	TLR7	TQ-A3334	Interventional/phase 1	NCT06160895
	TLR9	AST-008	Interventional/phase 1	NCT01567683 NCT03071679

Disease type	Category of PRRs	Antagonist	Study type/phase	ClinicalTrials.gov identifier
COVID-19	TLR4	ApTOLL	Interventional/phase 1	NCT05293236
Sjögren’s Syndrome	TLR 7 and 9	Hydroxychloroquine	Interventional/phase 3	NCT01601028
Psoriasis	TLR7/8/9	IMO-8400	Interventional/phase 2	NCT01899729
Type 2 diabetes	Eritoran	Eritoran	Interventional/phase 2	NCT02267317 NCT02321111
Acute Ischemic Stroke	TLR4	ApTOLL	Interventional/phase 2	NCT04734548
Healthy Subjects	TLR7	ApTOLL	Interventional/phase 1	NCT04742062

### PRR agonists for therapy

Agonists are divided into immunostimulants and delivery systems based on their mechanism of action. Immunostimulants act as danger signals, mimicking or functioning as PAMPs or DAMPs to engage PRRs on APCs. This triggers APC maturation, enhancing their antigen presentation, co-stimulatory signal expression, and cytokine production, which drive adaptive immune responses. Different immunostimulants target specific PRRs, inducing distinct cytokine profiles that shape the adaptive response. Understanding these mechanisms is crucial for optimizing adjuvant design and improving vaccine efficacy.^579^

Numerous immunostimulants targeting PRRs have been developed, with some already in clinical use and others still under investigation. Classical adjuvants such as aluminium salts, emulsions, and TLR agonist-based adjuvants, along with particulate systems such as liposomes, have gained widespread approval for inclusion in various vaccines. Vaccine antibody titers are effectively boosted, and cellular immune responses are enhanced by these adjuvants, as documented in a recent review.^580^ In addition to these classical adjuvants, a diverse array of immunostimulants has been evaluated, including synthetic dsRNAs, glucopyranosyl lipid A and its derivatives, imidazoquinolines, metabolic adjuvants, and manganese-based adjuvants. These immunostimulants, each with unique properties, have undergone preclinical and clinical assessments. TLR-targeting immunostimulants remain the most prevalent in both preclinical and clinical studies. Furthermore, there is a growing trend towards combining multiple PRR agonists to enhance their targeting of APCs. These combinations are often formulated into oil-in-water emulsions or incorporated into other delivery systems to optimize their efficacy (as exemplified by clinical trials NCT01585350,^581,582^ NCT02126579,^583^ and NCT01008527).

Delivery systems are carrier materials designed to encapsulate antigens, immunostimulants, drugs, and other substances, thereby enhancing their uptake and presentation by APCs and optimizing drug functionality.^584^ Essentially, these delivery systems mainly facilitate antigen presentation. Antigens are recognized, taken up, and internalized by APCs, then loaded and presented on the cell surface via MHC molecules in this process.^585^ Delivery systems can augment the antigen signals presented on APC surfaces through various mechanisms. These include prolonging antigen bioavailability, mimicking pathogen size and spatial structure to enhance APC uptake, directly targeting specific receptors such as CLRs on APCs, facilitating lymph node trafficking, and promoting antigen cross-presentation.^586^

In recent years, advancements in engineering materials science have led to the development of diverse vaccine delivery systems utilizing engineering materials. Key platforms include novel water-in-oil, caged protein nanoparticles, lipid nanoparticles (LNPs), polymer nanoparticles, nanoemulsions, virus-like particles, and inorganic nanocarriers.^576^ These delivery systems, which vary in their mechanisms of action and physicochemical properties, influence the efficacy of PRR activation. For instance, the vaccines consist of modified mRNA, which encodes the Spike protein derived from SARS-CoV-2, encapsulated within LNP formulation containing ionizable lipids and cholesterol. The LNP carrier itself demonstrated adjuvant properties in conjunction with a protein antigen. Furthermore, it was observed that the vaccines necessitated MDA-5 for their immunogenicity, surprisingly without requiring a broad array of other PRRs.^390,580^ As materials science progresses, material-based delivery systems are developing rapidly.^587^ Delivery systems are designed to protect vaccine components and direct them to APCs or specific lymphoid tissues, thereby enhancing adaptive immune responses. Compared to immunostimulants, delivery systems have advanced to clinical testing relatively less, likely due to unresolved safety concerns. More research is needed to clarify the mechanisms by which delivery systems interact with host immune cells and organs. Such studies will improve our understanding of their efficacy and potential side effects, enabling the creation of safer and more effective delivery methods.

### PRR antagonists for therapy

PRR antagonists belong to a class of immunosuppressants primarily designed to modulate receptor function and disrupt the interaction between receptors and their ligands.^588^ These agents are particularly effective in managing disorders characterized by aberrant immune system activation. The inappropriate activation of TLR7, 8, and 9 is crucial for starting and continuing inflammatory conditions. For instance, IMO-8400, a TLR7, 8, and 9 antagonists, exhibited clinical efficacy in a phase 2a randomized, placebo-controlled trial involving patients with moderate-to-severe plaque psoriasis.^589^ Preclinical assessments of MHV370, a selective oral inhibitor of TLR7/8, have been promising. In vivo studies demonstrate that both prophylactic and therapeutic administration of MHV370 effectively blocks TLR7-mediated responses, including cytokine release, B-cell stimulation, and the expression of IFN-stimulated genes. Furthermore, in the NZB/W F1 mouse model of lupus, MHV370 administration led to disease arrest.^506^ Inhibitors such as IMO-3100 and IMO-8400, which target these receptors, markedly diminish IL-23-induced IL-17A expression, highlighting their potential in modulating the inflammatory cascade.^590^ Research has shown that inhibitory oligodeoxynucleotides (ODNs) containing phosphorothioate backbone modifications possess therapeutic potential for immune-related diseases, including SLE and RA. Specifically, ODN1411 directly interacts with the extracellular domain of TLR8, competitively inhibiting its signaling pathway.^591^

The expression of specific TLRs on the cell surface, particularly those with tumor-promoting roles in certain malignancies, offers viable molecular targets for therapeutic intervention using mAbs. The administration of a humanized anti-TLR2 mAb, tomaralimab, in human tumor xenograft models of gastric and pancreatic cancers markedly inhibited the growth of tumors exhibiting high TLR2 expression and/or activation. LR2 activation promotes tumor progression and is linked to patient survival and chemotherapy response in pancreatic ductal adenocarcinoma.^543^ Although preclinical results are encouraging, clinical trials assessing TLR2 inhibitors in solid tumors are still missing. Nevertheless, phase I/II trials of an anti-TLR2 antibody in patients with low-risk myelodysplastic syndrome have shown a favorable safety profile, with no signs of drug resistance, dose-limiting toxicities, or treatment-related infections (NCT02363491 and NCT03337451). These findings suggest the potential clinical feasibility and safety of anti-TLR2 therapy as an adjuvant treatment for advanced solid tumors that screen positive for high TLR2 expression and activity. Beyond TLR2, targeting TLR4 with small-molecule inhibitors or neutralizing mAbs has also shown anticancer efficacy, especially in chemically induced models of colitis-associated colon cancer.^592^ However, the translation of TLR4 inhibitors into clinical practice for cancer treatment has not yet succeeded. In contrast to strategies that stimulate tumor-inhibitory TLRs, the tumor-promoting activities of certain TLRs in specific cancer types present opportunities to explore using their respective activating ligands or agonists as immunological adjuvants in cancer therapy.

Based on research examining the impact of NLRPs on inflammatory conditions, numerous strategies have emerged to regulate NLRPs, making them frequent targets for inhibitory interventions.^112^ Among these, NLRP3 stands as the most extensively studied and characterized member of the NLRP subfamily. Recent investigations have underscored the pivotal role of unchecked NLRP3 inflammasome activation in the development of inflammatory bowel disease, with heightened secretion of mature IL-1β and IL-18 linked to exacerbated colitis. Consequently, a plethora of NLRP3 inhibitors have been deployed as therapeutic agents for inflammatory bowel disease management. Traditional Chinese medicine has long been employed in the treatment of arthritis, spanning millennia. Taraxasterol, a bioactive constituent of Taraxacum officinale, demonstrates protective effects against IL-1β-induced human RA fibroblast-like synoviocytes and ameliorates symptoms in collagen-induced arthritis mice by modulating the NF-κB and NLRP3 inflammasome pathways.^593^ Wu et al. reported that low-methoxyl pectin attenuates NLRP3 inflammasome activation by enriching cecal bacterial diversity and enhancing short-chain fatty acid production, thereby reconfiguring the pancreatic immune milieu in type 1 diabetes.^594^ Furthermore, ginsenoside Rg1 has emerged as a promising therapeutic candidate for preventing the onset of streptozotocin-induced type 1 diabetes. It achieves this by diminishing NLRP3 activity in the liver and pancreas of mice and suppressing the production of IL-1β and IL-18.^595^ Additionally, inflammasome-related PRRs present compelling targets for oncological drug development, given their varied roles in both inhibiting and promoting tumorigenesis across multiple cancer types.^596^

The translation of PRR-targeting strategies from preclinical research to clinical practice remains a work in progress, with only a small fraction of these approaches currently adopted in clinical settings. To advance PRR-targeted therapies, several key priorities must be addressed: (1) Developing delivery systems that ensure accurate and efficient administration of PRR-targeted treatments is essential. Improved delivery mechanisms will enhance therapeutic efficacy while ensuring controlled and precise dosing. (2) A deeper understanding of the molecular mechanisms underlying PRR functionality is critical. Such knowledge will drive the development of more effective and targeted therapeutic interventions. (3) Designing highly specific and potent inhibitors for PRRs is a crucial goal. Achieving this will require a multidisciplinary approach, integrating expertise from molecular and structural biology, materials science, molecular engineering, pharmacology, and clinical research. By addressing these priorities through collaborative and innovative approaches, PRR-targeted therapies can be refined and translated into meaningful clinical applications with significant potential to improve patient outcomes.

## Conclusion and outlook

Over the past decades, research has elucidated the classification, signaling mechanisms, and diverse biological roles of PRRs, underscoring their significance in both health and disease contexts. PRRs exhibit remarkable versatility in their ability to detect a wide range of microbial and endogenous danger signals, enabling robust immune responses against infections while maintaining homeostasis during tissue repair. However, the dual roles of PRRs—as drivers of protective immunity and mediators of pathological inflammation—highlight the complexity of their regulation. Dysregulation of PRR signaling can result in either insufficient defense against pathogens or excessive inflammation, contributing to a spectrum of diseases, including autoimmune disorders, chronic inflammatory conditions, and cancer. PRR-mediated sterile inflammation has been linked to the pathogenesis of metabolic disorders, neurodegenerative diseases, and fibrotic diseases, emphasizing the need for a balanced immune response.

Recent advancements have illuminated the intricate signaling networks associated with PRRs, particularly the interplay between pathways such as NF-κB, MAPK, and MYD88-dependent cascades. These insights have expanded our understanding of PRR functions, including their roles in inflammasome activation and crosstalk with other signaling molecules. The discovery of iPRRs adds another layer of complexity, revealing mechanisms that temper immune responses to prevent overactivation. Furthermore, post-transcriptional and post-translational modifications, such as ubiquitination and phosphorylation, play crucial roles in fine-tuning PRR activity, highlighting potential regulatory nodes for treatment.

Among the most prospective research domains of PRR research lies in therapeutic applications. PRR-targeting strategies have shown potential in modulating immune responses for the treatment of infectious diseases, autoimmune disorders, and cancer. For instance, TLR agonists have been explored as vaccine adjuvants to enhance adaptive immune responses, while inhibitors targeting inflammasome components such as NLRP3 offer potential in mitigating inflammatory diseases. Nevertheless, significant challenges remain in translating these strategies from bench to bedside. The risk of systemic immune activation necessitates the development of precision delivery systems and context-specific modulators to minimize off-target effects and ensure controlled therapeutic outcomes.

As the field advances, several critical areas warrant further investigation. First, the discovery and characterization of novel PRRs and their ligands, particularly in non-immune cells, could uncover new dimensions of immune regulation and tissue homeostasis. The role of PRRs in gut-organ crosstalk mediated by the microbiota, for instance, represents a burgeoning research frontier with implications for systemic immunity and metabolic health. Additionally, to develop targeted therapies, it is essential to understand the tissue-specific functions and regulatory mechanisms of PRRs.

Second, the dynamic interaction between PRRs and their microenvironment presents another promising avenue for exploration. Factors such as hypoxia, nutrient availability, and the microbiota significantly influence PRR signaling and immune outcomes. Deciphering these interactions at a molecular level could lay the groundwork for novel strategies to modulate inflammation and promote tissue regeneration.

Third, the development of advanced delivery systems to target PRRs or their pathways with high specificity remains a priority. Nanoparticle-based platforms and lipid carriers, for example, offer exciting possibilities for delivering PRR-targeted therapies to specific tissues or cell types. However, addressing safety concerns and understanding the immunological interactions of these systems will be crucial for their clinical translation.

Finally, the integration of multidisciplinary approaches, including molecular biology, bioinformatics, and structural biology, will be indispensable for uncovering the mechanistic underpinnings of PRR signaling. Emerging technologies such as single-cell sequencing, CRISPR-based editing, and high-resolution imaging provide powerful tools to dissect PRR functions at an unprecedented level of detail. These approaches, coupled with systems biology analyses, could facilitate the development of predictive models for PRR-mediated immune responses, guiding the design of next-generation therapies.

In summary, PRRs represent a cornerstone of immune defense, with their multifaceted roles spanning infection control, inflammation regulation, and tissue homeostasis. While substantial progress has been made in understanding their biology, much remains to be explored regarding their regulation and therapeutic potential. By bridging fundamental research with translational applications, the field is poised to unlock new avenues for treating a wide array of diseases, ultimately advancing the frontier of precision immunotherapy.
